# Consequences of spatial patterns for coexistence in species-rich plant communities

**DOI:** 10.1038/s41559-021-01440-0

**Published:** 2021-05-03

**Authors:** Thorsten Wiegand, Xugao Wang, Kristina J. Anderson-Teixeira, Norman A. Bourg, Min Cao, Xiuqin Ci, Stuart J. Davies, Zhanqing Hao, Robert W. Howe, W. John Kress, Juyu Lian, Jie Li, Luxiang Lin, Yiching Lin, Keping Ma, William McShea, Xiangcheng Mi, Sheng-Hsin Su, I-Fang Sun, Amy Wolf, Wanhui Ye, Andreas Huth

**Affiliations:** 1 Department of Ecological Modelling, Helmholtz Centre for Environmental Research – UFZ, Leipzig, Germany; 2 German Centre for Integrative Biodiversity Research (iDiv) Halle-Jena-Leipzig, Leipzig, Germany; 3 CAS Key Laboratory of Forest Ecology and Management, Institute of Applied Ecology, Chinese Academy of Sciences,; 4 Conservation Ecology Center, Smithsonian Conservation Biology Institute, Front Royal, VA USA; 5 Forest Global Earth Observatory (ForestGEO), Smithsonian Tropical Research Institute, Washington, DC USA; 6 CAS Key Laboratory of Tropical Forest Ecology, Xishuangbanna Tropical Botanical Garden, Chinese Academy of Sciences,; 7 Center of Conservation Biology, Core Botanical Gardens, Chinese Academy of Sciences,; 8 Centre for Integrative Conservation, Xishuangbanna Tropical Botanical Garden, Chinese Academy of Sciences,; 9 School of Ecology and Environment, Northwestern Polytechnical University,; 10 Department of Natural and Applied Sciences, University of Wisconsin–Green Bay, Green Bay, WI USA; 11 Department of Botany, National Museum of Natural History, Smithsonian Institution, Washington, DC USA; 12 Key Laboratory of Vegetation Restoration and Management of Degraded Ecosystems, South China Botanical Garden, Chinese Academy of Sciences,; 13 Department of Life Science, Tunghai University,; 14 State Key Laboratory of Vegetation and Environmental Change, Institute of Botany, Chinese Academy of Sciences,; 15 Taiwan Forestry Research Institute,; 16 Center for Interdisciplinary Research on Ecology and Sustainability, National Dong Hwa University,; 17 Institute of Environmental Systems Research, University of Osnabrück, Osnabrück, Germany

**Keywords:** Community ecology, Ecological modelling, Forest ecology, Population dynamics, Theoretical ecology

## Abstract

Ecology cannot yet fully explain why so many tree species coexist in natural communities such as tropical forests. A major difficulty is linking individual-level processes to community dynamics. We propose a combination of tree spatial data, spatial statistics and dynamical theory to reveal the relationship between spatial patterns and population-level interaction coefficients and their consequences for multispecies dynamics and coexistence. Here we show that the emerging population-level interaction coefficients have, for a broad range of circumstances, a simpler structure than their individual-level counterparts, which allows for an analytical treatment of equilibrium and stability conditions. Mechanisms such as animal seed dispersal, which result in clustering of recruits that is decoupled from parent locations, lead to a rare-species advantage and coexistence of otherwise neutral competitors. Linking spatial statistics with theories of community dynamics offers new avenues for explaining species coexistence and calls for rethinking community ecology through a spatial lens.

## Abstract (web summary)

Tree spatial data, spatial statistics and dynamical theory reveal the relationship between spatial patterns and population-level interaction coefficients and their consequences for multispecies dynamics and coexistence.

## Main

Understanding the mechanisms that maintain high species diversity in plant communities such as tropical forests has long challenged ecologists^1^ and has stimulated major efforts in field and theoretical ecology^2–5^. However, despite a multitude of coexistence mechanisms that have been proposed^6^ and recent advances in coexistence theories^7–18^, this fundamental question has not been fully resolved^8,9,14^. For example, theoretical models indicate that stable coexistence is difficult to reach in large communities^11,13^. We argue that consideration of spatial patterns of plant individuals, such as intraspecific clustering and interspecific segregation, may allow for a better understanding of mechanisms of coexistence in species-rich communities^17^.

Although many studies suggest that spatial patterns and neighbourhood effects may play an important role in diversity maintenance^17–21^, the integration of spatial patterns into coexistence theories of species-rich communities is difficult. A major difficulty is linking spatial processes at the individual level to community dynamics. One reason for this is a scale mismatch. The analytical models that form the basis of most coexistence theories^7,8,11–16,22^ have state variables that operate at the macroscale (that is, the population or community-level abundances), use parameters that describe average ‘mesoscale’ properties of the individuals (such as population-level interaction coefficients and demographic rates) and often rely on ‘mean-field’ approximations^18,23^ where spatial patterns are neglected. However, spatial patterns and population-level interaction coefficients emerge at the mesoscale from the microscale behaviour of individuals and their interactions with other individuals and the environment. Therefore, studying the impact of spatial patterns on species coexistence requires multiscale approaches such as spatial moment equations^18,23,24^ that incorporate pattern-forming processes operating at the level of individuals and translate these into population and community dynamics.

We propose here such a multiscale approach. To this end, we first derive population-level interaction coefficients *α*_*fi*_ from individual-level interaction coefficients *β*_*fi*_ and neighbourhood crowding indices^19,21^ that are commonly used to describe interactions among tree individuals at the microscale, and then incorporate the emerging coefficients *α*_*fi*_ into analytical macroscale models. Our approach is based on separation of timescales (adiabatic approximation^25^), given that mesoscale spatial patterns usually build up quickly and approach a quasi-steady state whereas the macroscale state variables (for example, abundances) change slowly^23^. Therefore, we do not need to describe the dynamics of the quick mesoscale patterns explicitly (as, for example, is done in approaches based on moment equations^18,23,24^) but concentrate instead on spatial patterns that transport the critical information from the microscale into macroscale models. This approach requires information on mesoscale spatial patterns that can be obtained from fully mapped forest plots such as those of the Forest Global Earth Observatory (ForestGEO) network^4^.

More specifically, we (1) derive species-level interaction coefficients from individual-level interactions using empirical information on spatial patterns in nine ForestGEO megaplots^4^, (2) integrate the resulting species-level interaction coefficients into analytical macroscale multispecies models and (3) study their consequences for multispecies dynamics and coexistence.

## Results and discussion

### Species-level interaction coefficients

**Fig. 1:**
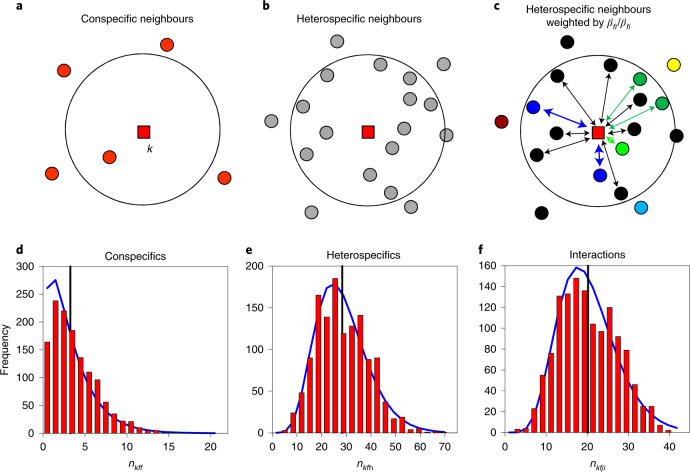
Neighbourhood crowding indices describe individual-level interactions and their intraspecific variability. **a**, The conspecific crowding index *n*_*kff*_ is the number of conspecific neighbours (filled red circles) within distance *R* (black circle) of the focal individual *k* (filled red square). **b**, The heterospecific crowding index *n*_*kf*h_ is the number of heterospecific neighbours (filled grey circles) within distance *R* of the focal individual *k*. **c**, The heterospecific interaction crowding index *n*_*kfβ*_ additionally weights heterospecifics by their relative competitive effect *β*_*fi*_/*β*_*ff*_, symbolized by the arrows. Different colours indicate different species. **d**, Distribution of the number *n*_*kff*_ of conspecific neighbours with diameter at breast height (dbh) ≥ 10 cm of the species *Castanopsis cuspidata* of the 25 ha Fushan plot. **e**, Corresponding distribution of heterospecific neighbours *n*_*kf*h_. **f**, Corresponding distribution of the crowding index *n*_*kfβ*_. In **d**–**f**, blue lines show gamma distributions with the same means and variance-to-mean ratios as the observed distributions, and the vertical black line indicates the mean value. See Supplementary Data Table1 for additional examples.

We first derive species-level interaction coefficients from individual-level neighbourhood crowding indices^19,21,26^ that quantify how the performance of a focal individual depends on interactions with its neighbours. To this end, we describe the survival rate of a focal individual *k* of species *f* in dependence on the local number of neighbours as

1a $$s_{kf} = s_f \exp \left( - \beta _{ff} \left(n_{kff} + \underbrace {\mathop {\sum}\nolimits_{i \ne f} {(\beta _{fi}/\beta _{ff})n_{kfi}} }_{n_{kf\beta }} \right) \right),$$

where *s*_*f*_ is a density-independent background survival rate of species *f*; the crowding indices *n*_*kff*_, *n*_*kfi*_ and *n*_*kf*h_ are the number of conspecifics, neighbours of species *i* and heterospecific neighbours within distance *R* of a focal individual *k*, respectively; the subscript ‘h’ indicates all heterospecifics together (that is, *n*_*kf*h_ = ∑_*i*≠*f*_ *n*_*kfi*_) and the crowding index *n*_*kfβ*_ weights each heterospecific neighbour by its relative competition strength *β*_*fi*_/*β*_*ff*_ (equation (1a)), with *β*_*fi*_ being the individual-level interaction coefficients between species *f* and *i* (Fig. 1a–c). The corresponding population-level survival rate is given by

1b $${\mathrm{surv}}_f = s_f\exp \left( - \alpha _{ff} \left(N_f(t) + \mathop {\sum}\limits_{i \ne f} {(a_{fi}/ \alpha _{ff})} N_i(t) \right) \right),$$

where *N*_*i*_(*t*) is the abundance of species *i* at time step *t* and *α*_*fi*_ is the population-level interaction coefficient between species *f* and *i*. To estimate surv_*f*_ we average the survival rates *s*_*kf*_ (equation (1a)) of all individuals *k* of species *f*:

2 $${\mathrm{surv}}_f = \frac{1}{{N_f\left( t \right)}}\mathop {\sum}\limits_{k = 1}^{N_f\left( t \right)} {s_{kf} = s_f} \mathop {\int}\limits_x {\mathop {\int}\limits_y {\exp } } \left( { - \beta _{ff}\left( {x + y} \right)} \right)p_x p_y {\mathrm{d}}x {\mathrm{d}}y$$

where *p*_*x*_ and *p*_*y*_ are the distributions of the crowding indices *x* = *n*_*kff*_ and *y* = *n*_*kfβ*_ for individuals of species *f*, respectively.

To determine the distributions *p*_*x*_ and *p*_*y*_, we analysed forest inventory data from nine 20–50 ha forest dynamics plots (Supplementary Table 1) in the ForestGEO network^4^. We used phylogenetic similarity between tree species as a surrogate for the relative competition strength *β*_*fi*_/*β*_*ff*_ because it is available for the species in the nine plots (Methods). This is an established approach in species-rich communities^19,26,27^ to approximate niche differences in the absence of other data.

**Extended Data Fig. 1:**
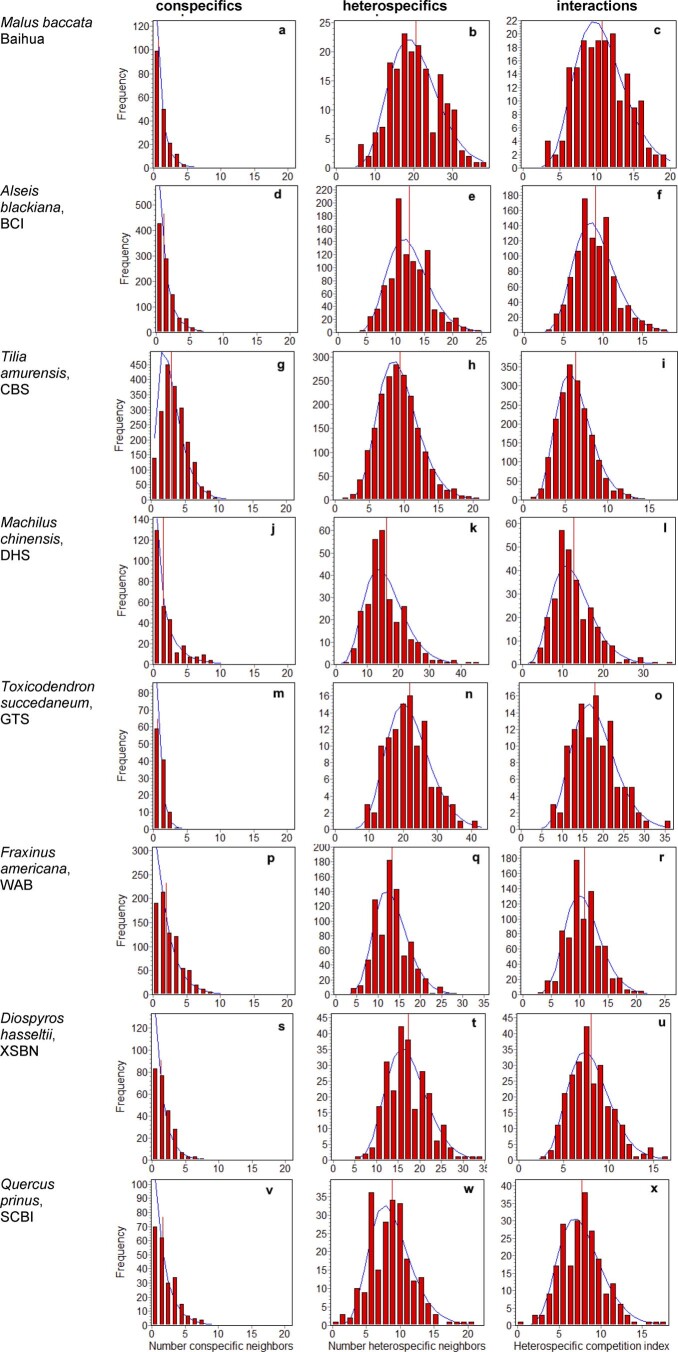
Examples for intraspecific variability in the crowding indices. **a, b, c**, *Malus baccata*, Baihua plot. **d, e, f**, *Alseis blackiana*, BCI plot. **g, h, i**, *Tilia amurensis*, CBS plot. **j, k, l**, *Machilus chinensis*, DHS plot. **m, n, o**, *Toxicodendron succedaneum*, GTS plot. **p, q, r**, *Fraxinus americana* WAB plot. **s, t, u**, *Diospyros hasseltii* XSBN Plot. **v, w, x**, *Quercus prinus*, SCBI plot. Left: number of conspecific neighbours (*n*_*kff*_), middle: number of heterospecific neighbours (*n*_*kf*h_), and right: heterospecifics neighbours weighted by their relative competitive effect *β*_*fi*_/*β*_*ff*_ (*n*_*kf*β_). We used a 10 m plant neighbourhood^19,26^ and phylogenetic similarity as surrogate for pairwise interaction strength^19,26^. Solid blue lines show gamma distributions with the same mean and variance-to-mean as the observed distributions, and the vertical red lines indicates the mean values. The 95% percentiles for the error indices quantifying the departures from a Gamma distribution for all 289 focal species were 0.051, 0.027, and 0.022 for *n*_*kff*_, *n*_*kf*h_, *n*_*kfβ*_, respectively, indicating a reasonable fit.

**Extended Data Fig. 2:**
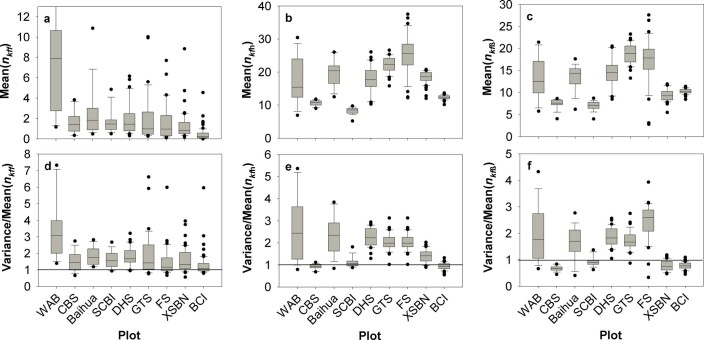
Characteristics of the neighbourhood crowding indices. Distribution of the mean and the variance-to-mean ratio of the crowding indices for the different species at each forest plot with boxplots indicating 10th, 25th, 50th, 75th, 90th percentiles and outliers. **a**. The mean $\bar n_{ff}$ of the conspecific neighbourhood crowding index *n*_*kff*_ over species. **b**. The mean $\bar n_{fh}$ of the heterospecific neighbourhood crowding index *n*_*kf*h_ over species. **c**. The mean $\bar n_{f\beta }$ of the interaction neighbourhood crowding index *n*_*kfβ*_ over species. **d**. The variance-to-mean ratio *b*_*kf*_ of the conspecific neighbourhood crowding index *n*_*kff*_ over species. **e**. The variance-to-mean ratio *b*_*k*h_ of the heterospecific neighbourhood crowding index *n*_*kf*h_ over species. **f**. The variance-to-mean ratio *b*_*kβ*_ of the interaction neighbourhood crowding index *n*_*kfβ*_ over species. The neighbourhood radius was *R* = 10 m. We used for the analysis all individuals with dbh ≥ 10 cm and included focal species with more than 50 individuals. For plot names see Supplementary Table 1.

**Extended Data Fig. 3:**
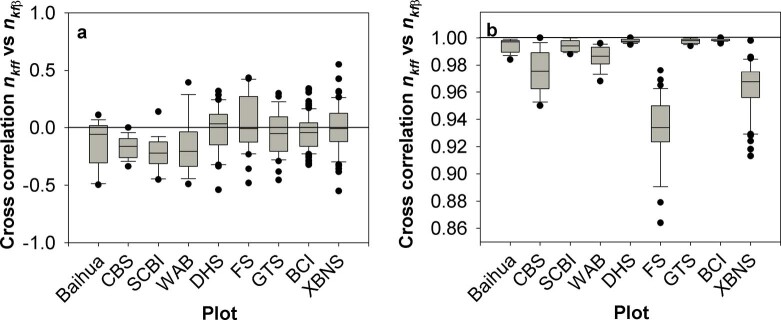
Correlation between different crowding indices. We estimated for all individuals of a species *f* the correlation between their crowding indices *n*_*kff*_ and *n*_*kfβ*_ (**a**) and *n*_*kf*h_ and *n*_*kfβ*_ (**b**). The crowding index *n*_*kff*_ counts the conspecific neighbours of individual *k* within distance *R* = 10 m, *n*_*kf*h_ counts the corresponding number of heterospecific neighbours, and *n*_*kfβ*_ weights each heterospecific neighbour by its relative competition strength *β*_*fi*_/*β*_*ff*_. The boxplots show the distribution of the Pearson correlation coefficients for each focal species, separately for the nine forest plots, indicating 10th, 25th, 50th, 75th, 90th percentiles and outliers. We used for the analysis all individuals with dbh ≥ 10 cm and included focal species with more than 50 individuals. For plot names see Supplementary Table 1.

The number of con- and heterospecific neighbours and the heterospecific interaction index *n*_*kfβ*_ vary widely among conspecifics and can be described by gamma distributions (Fig. 1 and Extended Data Figs. 1 and 2). Detailed analysis of the empirical crowding indices reveals additional relationships that are relevant for our subsequent analysis. First, we find that the crowding indices *n*_*kff*_ and *n*_*kfβ*_ are not, or are only weakly, correlated for a given species *f* (Extended Data Fig. 3a). Second, we find for trees of a given species *f* high correlations between the two crowding indices *n*_*kf*h_ and *n*_*kfβ*_ (Extended Data Fig. 3b) with a common factor *B*_*f*_ (that is, *n*_*kfβ*_ ≈ *B*_*f*_*n*_*kf*__h_). This result suggests operation of diffuse neighbourhood competition, in which the competition strength of heterospecifics is on average a factor *B*_*f*_ lower than that of conspecifics.

The integral of equation (2) can be solved analytically for independent gamma distributions *p*_*x*_ and *p*_*y*_ and yields

3 $${\mathrm{surv}}_f = s_f \exp \left( { - \beta _{ff}\left( {\gamma _{ff}\bar n_{ff} + \gamma _{f\beta }\bar n_{f\beta }} \right)} \right)$$

where $\bar n_{ff}$ and $\bar n_{f\beta }$ are the average values of the crowding indices *n*_*kff*_ and *n*_*kfβ*_, respectively, and *γ*_*ff*_ and *γ*_*fβ*_ contain the variance-to-mean ratios of the gamma distributions *p*_*x*_ and *p*_*y*_, respectively, but in our case have values close to one (Methods).

The last step in deriving pairwise population-level interaction coefficients is to relate the averages of the different crowding indices to the macroscale population abundances *N*_*f*_(*t*). We accomplish this by taking advantage of connections between crowding indices and the summary functions of spatial point process theory^21^. The mean of the crowding index *n*_*kff*_ (that is, the mean number of further conspecific neighbours within distance *R*) is proportional to Ripley’s *K*, a well-known quantity in point process theory^28,29^:

4a $$\bar n_{ff} = K_{ff}\left( R \right)N_f(t)/A$$

where *K*_*ff*_(*R*) is the univariate *K* function for species *f* and *A* is the area of the observation window. The *K* function describes the spatial pattern of conspecifics within a neighbourhood distance *R*, indicating clustering if *K*_*ff*_(*R*) > π*R*^2^, a random pattern if *K*_*ff*_(*R*) = π*R*^2^ and regularity if *K*_*ff*_(*R*) < π*R*^2^. In the following, we use the normalized *K* function $k_{ff}\left( R \right) = K_{ff}\left( R \right)/\uppi R^2$ to quantify the spatial neighbourhood patterns, and therefore $\bar n_{ff} = k_{ff}\left( R \right)\frac{{\uppi R^2}}{A}N_f(t)$.

Analogously, the mean number of heterospecific neighbours is given by

4b $$\bar n_{f{\mathrm{h}}} = k_{f{\mathrm{h}}}\left( R \right)\frac{{\uppi R^2}}{A}\mathop {\sum }\limits_{i \ne f} N_i(t),$$

where the bivariate normalized *K* function *k*_*f*h_(*R*) indicates segregation to heterospecifics (subscript ‘h’) within distance *R* if *k*_*f*h_(*R*) < 1. Independent placement occurs if *k*_*f*h_(*R*) = 1, and attraction if *k*_*f*h_(*R*) > 1.

Motivated by the finding *n*_*kfß*_ ≈ *B*_*f*_ *n*_*kf*__h_ (Extended Data Fig. 3b) we rewrite the mean crowding index $\bar n_{f\beta }$ as

5 $$\bar n_{f\beta } = \underbrace {B_f}_{\frac{{\bar n_{f\beta }}}{{\bar n_{f{\mathrm{h}}}}}}\bar n_{f{\mathrm{h}}},$$

where the point process summary function *B*_*f*_ indicates how much the competition strength of one heterospecific neighbour differs on average from that of one conspecific neighbour. The values of *B*_*f*_ depend mainly on the individual-level interaction coefficients *β*_*fi*_ but also on the spatial pattern of the different species and their relative abundances (equation (12)).

Inserting the expressions for $\bar n_{ff}$ and $\bar n_{f\beta }$ into equation (3) and comparing with equation (1b) leads to our first main result, the analytical expressions of the population-level interaction coefficients:

6a $$\alpha _{ff} = c\,\gamma _{ff}\,k_{ff}\beta _{ff} \qquad {\mathrm{intraspecific}}\,{\mathrm{interactions}}\,{\mathrm{of}}\,{\mathrm{species}}\,f$$

6b $$\alpha _{fi} = c\gamma _{f\beta }k_{f{\mathrm{h}}}\beta _{ff}B_f \, \quad {\mathrm{interspecific}}\,{\mathrm{interactions}}\,{\mathrm{with}}\,{\mathrm{species}}\,i$$

with scaling constant *c* = π*R*^2^/*A*. Notably, equation (6b) indicates that the emerging population-level interaction coefficients *α*_*fi*_ are the same for all heterospecifics (that is, *α*_*fi*_ = *α*_*f*h_ for *i* ≠ *f*). Thus, even if the individual-level interactions coefficients *β*_*fi*_ differ among species pairs, the emerging population-level interaction coefficients *α*_*fi*_ have a substantially simpler structure. This phenomenon is an example of simplicity emerging from complex species interactions^30^ and is likely to occur only in species-rich communities^9,12,31^.

**Fig. 2:**
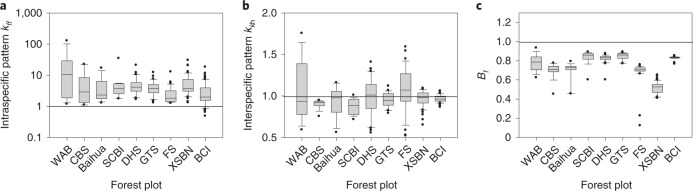
Emergent spatial patterns in the nine forest dynamics plots. **a**–**c**, Distribution of the values of the different measures of spatial patterns, taken over the focal species of the different forest plots. Boxplots show the 10th, 25th, 50th, 75th and 90th percentiles; outliers are indicated by filled black points. Intraspecific clustering is indicated by *k*_*ff*_ > 1 and interspecific segregation by *k*_*f*h_ < 1, and the less a heterospecific neighbour competes on average relative to a conspecific neighbour, the more *B*_*f*_ decreases. The neighbourhood radius used was *R* = 10 m. For the analysis, we used all individuals with dbh ≥ 10 cm and included focal species with more than 50 individuals. For forest plot names, see Supplementary Data Table 1.

**Fig. 3:**
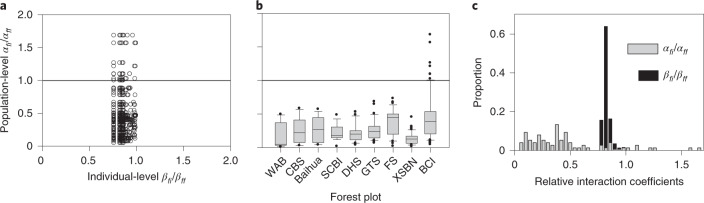
The emergent population-level interaction coefficients. **a**, Relationship between the relative population-level interaction coefficients *α*_*fi*_/*α*_*ff*_ and the corresponding relative individual-level interaction coefficients *β*_*fi*_/*β*_*ff*_, (equation (6)) for the 75 focal species of the BCI plot. The *β*_*fi*_/*β*_*ff*_ were based on phylogenetic dissimilarity. **b**, Distribution of the values of *α*_*fi*_/*α*_*ff*_, taken over the 289 focal species of the different forest plot. Boxplots show the 10th, 25th, 50th, 75th and 90th percentiles; outliers are indicated by filled black points. For full, separate distributions for tropical, subtropical and temperate forests, see Extended Data Fig. 4. **c**, Example for the distribution of the relative individual- and population-level interaction coefficients for the BCI plot. The neighbourhood radius used was *R* = 10 m. For the analysis, we used all individuals with dbh ≥ 10 cm and included focal species with more than 50 individuals. For forest plot names, see Supplementary Data Table 1.

The population-level interaction coefficients *α*_*fi*_ depend on several factors that can influence the macroscale balance between intra- and interspecific competition: (1) intraspecific clustering *k*_*ff*_ and interspecific segregation *k*_*f*h_ (Fig. 2a,b), (2) the relative competition strength *B*_*f*_ of one heterospecific neighbour (Fig. 2c) and (3) the shape of the response of survival to crowding (*γ*_*ff*_ and *γ*_*fβ*_, which contain the variance-to-mean ratios of the distribution of the crowding indices). Note that absence of spatial patterns (that is, *k*_*ff*_ = 1 and *k*_*f*h_ = 1) and a linear approximation of equation (3) lead to *γ*_*ff*_ = *γ*_*fβ*_ = 1 and direct proportionality *α*_*fi*_ = *cβ*_*fi*_ of the individual- and population-level interaction coefficients, as assumed by Lotka–Volterra models.

**Extended Data Fig. 4:**
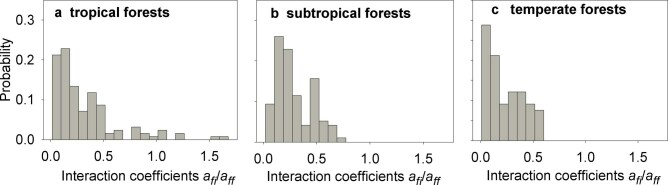
The distribution of the relative population-level interaction coefficients. **a**, The distribution of the relative population level interaction coefficients *α*_*fi*_/*α*_*ff*_ for the focal species of the tropical forests, resulting from equation 6. **b**, same as a, but for subtropical forests. **c**, same as a, but for temperate forests. Other conventions as in Fig. 3.

The information on spatial patterns extracted from the inventory data of our nine forests allows us to estimate the relative population-level interaction coefficients *α*_*fi*_/*α*_*ff*_ for all pairs of species *i* and *f* (Fig. 3 and Extended Data Fig. 4). For example, at BCI, the values of *α*_*fi*_/*α*_*ff*_ differ substantially from the corresponding individual-level coefficients *β*_*fi*_/*β*_*ff*_ (Fig. 3a,c), and for 83% of all species pairs, we find *α*_*fi*_/*α*_*ff*_ < *β*_*fi*_/*β*_*ff*_. Thus, the mesoscale spatial patterns can reduce, at the population level, the strength of heterospecific interactions relative to conspecific interactions by ‘diluting’ encounters with heterospecific neighbours relative to conspecific neighbours. Spatial patterns therefore have a strong potential to alter the outcome of deterministic individual-level interactions.

### Conditions for coexistence in the multiscale model

To study the consequences of the emerging spatial patterns for community dynamics and coexistence we insert the population-level interaction coefficients *α*_*fi*_ (equation (6)) into a simple macroscale model

7 $$\begin{array}{l}\frac{{N_f\left( {t + {\Delta}t} \right) - N_f\left( t \right)}}{{{\Delta}t}} = N_f\left( t \right)\\\left[ {\left( {r_f - 1} \right) + s_f \exp \left( - \alpha _{ff}N_f\left( t \right) - \alpha _{f{\mathrm{h}}}\mathop {\sum }\limits_{i \ne f} N_i(t)\right)} \right]\end{array}$$

In this model we assume that survival is governed by neighbourhood competition with *α*_*fi*_ = *α*_*f*h_, and the number of recruits of species *f* during a time step Δ*t* is given by *r*_*f*_*N*_*f*_(*t*), where *r*_*f*_ is the per capita reproduction rate of species *f*.

The carrying capacity of species *f* (that is, the equilibrium of equation (7) with *N*_*i*_(*t*) = 0 for all *i* ≠ *f*) is given by $K_f = - \ln \left( {\frac{{1 - r_f}}{{s_f}}} \right) {\alpha _{ff}}^{-1}$. Note that our theory also applies, after redefinition of the carrying capacity, to alternative macroscale models (Supplementary Table 3). From equation (7) we find $K_f = N_f^{\ast} + \frac{{\alpha _{f{\mathrm{h}}}}}{{\alpha _{ff}}}\mathop {\sum }\limits_{i \ne f} N_i^{\ast} = N_f^{\ast} \left( {1 - \frac{{\alpha _{f{\mathrm{h}}}}}{{\alpha _{ff}}}} \right) + \frac{{\alpha _{f{\mathrm{h}}}}}{{\alpha _{ff}}}J^{\ast}$, where $N_f^{\ast}$ is the abundance of species *f* in equilibrium and J^*^ the equilibrium community size (that is, $J^{\ast} = \sum _i N_i^{\ast}$; see also equation (14)). This leads, under the assumption that the population-level interaction coefficients *α*_*f*h_ are constant (Supplementary text), to a single equilibrium of the macroscale model for species *f*

8 $$N_f^{\ast} = \frac{{\overbrace {\alpha _{ff}K_f}^{ - \ln \left( {\frac{{1 - r_f}}{{s_f}}} \right)} - \alpha _{f{\mathrm{h}}}J^{\ast}}}{{\alpha _{ff} - \alpha _{f{\mathrm{h}}}}}$$

that is positive if denominator and numerator are both positive or both negative. However, the invasion criterion (equation (18)) is only fulfilled if both are positive. In this case, equation (8) suggests two different ways a species can go extinct. First, the denominator indicates that a species with strong clustering *k*_*ff*_ will show a small equilibrium abundance since in this case *α*_*ff*_ ≫ *α*_*f*h_ (equation (6)). Large values of *k*_*ff*_ can be expected for species of low abundance under dispersal limitation, where recruitment happens close to conspecific adults.

Second, the numerator of equation (8) indicates a positive abundance of species *f* if *α*_*ff*_*K*_*f*_ > *α*_*f*h_J^*^ and *α*_*f*h_/*α*_*ff*_ < 1. Therefore, we introduce a new feasibility index

9 $$\mu _f = \frac{{\alpha _{f{\mathrm{h}}}J^{\ast}}}{{\alpha _{ff}K_f}}$$

that indicates a positive abundance if *µ*_*f*_ < 1 given that heterospecific interactions at the population level are weaker than conspecific interactions (that is, *α*_*f*h_/*α*_*ff*_ < 1). The invasion criterion^7,8^ that tests whether a species with low abundance can invade the equilibrium community of all other species turned out to be basically the same as the feasibility condition (equation (9)) if the invading species does not show strong clustering (Methods and equation (18)). Note that we did not assume Allee effects^8^.

Further analysis that considers the dependency of J^*^ on the values of *K*_*f*_ and *α*_*f*h_/*α*_*ff*_ shows that the values of *µ*_*f*_ must be similar for all species *f* to fulfil the condition *µ*_*f*_ < 1, and that *µ*_*f*_ can show larger interspecific variability if the species richness *S* is smaller and/or if the mean of $\frac{{\alpha _{f{\mathrm{h}}}}}{{\alpha _{ff}}} \left( {1 - \frac{{\alpha _{f{\mathrm{h}}}}}{{\alpha _{ff}}}} \right)^{-1}$ is smaller (equations (14) and (15)). The feasibility index *µ*_*f*_ therefore governs species assembly by determining the subset of species of a larger species pool that can persist^13^, but any addition of a new species changes *µ*_*f*_ and may lead to reassembly of the community.

**Fig. 4:**
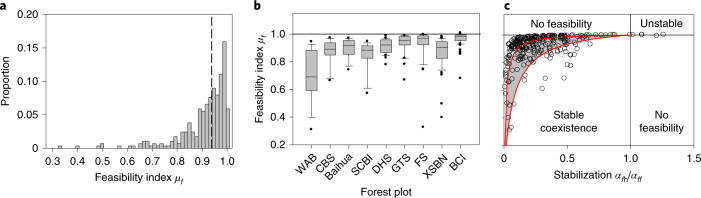
The feasibility index *µ*_*f*_ for the 289 focal species of different forest plots. **a**, The distribution of the index *µ*_*f*_ (equation (9)) for the 289 analysed tree species. The vertical dashed line indicates the median of the distribution. **b**, Distribution of *µ*_*f*_, taken over the 289 analysed species of the different forest plot. Boxplots show the 10th, 25th, 50th, 75th and 90th percentiles; outliers are represented by black points. **c**, Relationship between the feasibility index *µ*_*f*_ and the ratio *α*_*f*h_/*α*_*ff*_ of heterospecific to conspecific population-level interaction coefficients. Values of *µ*_*f*_ < 1 indicate a positive abundance. The red lines show the dependence of *µ*_*f*_ on *α*_*f*h_/*α*_*ff*_ expected for communities without interspecific variability in *µ*_*f*_ (equation (10a)) with 18 focal species (CBS plot, lower line) and 75 focal species (BCI plot, upper line). The neighbourhood radius used was *R* = 10 m. For the analysis, we used all individuals with dbh ≥ 10 cm and included tree species with more than 50 individuals. For plot names, see Supplementary Data Table 1.

Using the observed abundances in the forest plots (and assuming equilibrium) allows us to test our theory. We can estimate from the observed abundances the carrying capacities *K*_*f*_ and therefore also the indices *µ*_*f*_ for all focal species of our nine plots (Fig. 4). For 282 of our 289 focal species, we found *µ*_*f*_ < 1 and *α*_*f*h_/*α*_*ff*_ < 1, which means that the two conditions for stable coexistence are indeed satisfied for nearly all species. However, this is not a given, as shown by the seven species from BCI with *µ*_*f*_ > 1 and *α*_*f*h_/*α*_*ff*_ > 1. Thus, our theory is compatible with the observed coexistence of most species at our nine forest plots if the assumption of approximately constant population-level interaction coefficients holds.

In addition, we get information on the typical values of *µ*_*f*_ and *α*_*f*h_/*α*_*ff*_ that allows for insight into the stability of the communities. In agreement with the predictions of our theory, we find that *µ*_*f*_ tends to be smaller if *α*_*f*h_/*α*_*ff*_ is smaller (Fig. 4c). Furthermore, the values of *µ*_*f*_ were, for most species, larger than the expectation of *µ*_*f*_ for the corresponding communities without interspecific variability in *µ*_*f*_ (equation (10a)) but with the same number of species and the same mean values of *α*_*f*h_/*α*_*ff*_ (Fig. 4c), but all of the values were relatively close to the critical value of 1 (the median of all 289 species was 0.938; Fig. 4a).

### Consequences of spatial patterns for coexistence

Our theory predicts that coexistence requires, in the limit of high species richness, that species approach functional identity with respect to the feasibility index *µ*_*f*_ (Methods). This resembles neutral theory^2,32^, but our theory allows for trade-offs among demographic parameters and emerging spatial patterns to reach this equivalence (equation (9)). To study the consequences of spatial patterns for coexistence, we analysed a symmetric^33^ version of our model where all species have the same parameters and follow the same stochastic rules and where all individuals compete identically (leading to *B*_*f*_ = 1). Thus, we eliminate any potential coexistence mechanism other than that resulting from spatial patterns.

If the mesoscale patterns *k*_*ff*_ and *k*_*f*h_ converge to a stochastic equilibrium, we find that the feasibility and invasion criteria are always fulfilled if *α*_*f*h_/*α*_*ff*_ < 1 since

10a $$\mu _f = \frac{{S\frac{{\alpha _{f{\mathrm{h}}}}}{{\alpha _{ff}}}}}{{1 + \frac{{\alpha _{f{\mathrm{h}}}}}{{\alpha _{ff}}}\left( {S - 1} \right)}} < 1$$

10b $$\mu _f^i = \frac{{(S - 1)\frac{{\alpha _{f{\mathrm{h}}}}}{{\alpha _{ff}}}}}{{1 + \frac{{\alpha _{f{\mathrm{h}}}}}{{\alpha _{ff}}}\left( {S - 2} \right)}} < 1$$

Equations (10a) and (10b) follow from equations (16) and (18), respectively, if *α*_*f*h_/*α*_*ff*_ is the same for all species *f*. Thus, the spatial patterns that emerge at the mesoscale from the individual-level interactions can stabilize if *α*_*f*h_/*α*_*ff*_ < 1. The underlying mechanism is a positive fitness–density covariance^34^ (Methods).

To reveal the conditions that can lead to coexistence in a spatially explicit context, we use a spatially explicit and individual-based^35^ implementation of the symmetric version of the multiscale model (equation (7) and Methods). Models of this type are able to produce realistic spatial patterns consistent with mapped species distributions of large forest plots^17,35^. While our analytical approach in equation (7) only allows us to make simplified assumptions about the spatial component of the recruitment process, the simulation model allows us to explore the role of the spatial component of recruitment in more detail.

**Extended Data Fig. 5:**
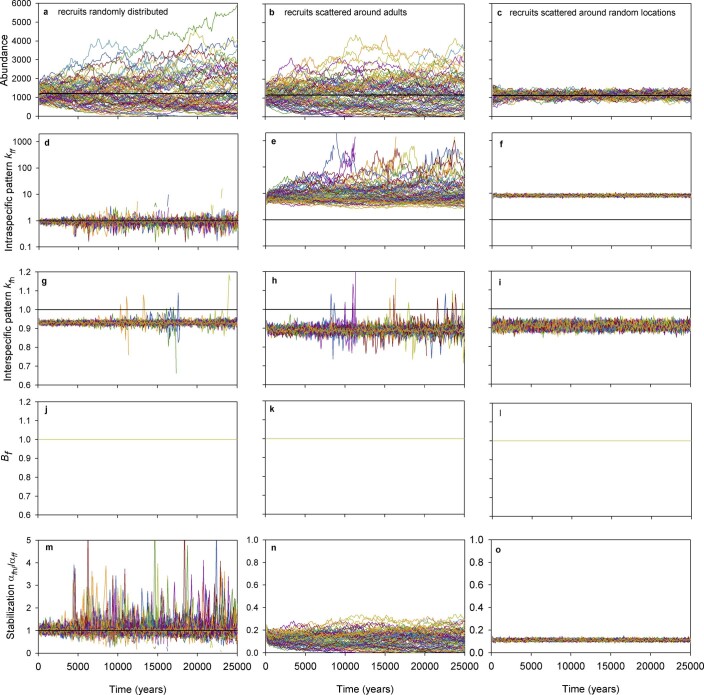
Spatial patterns can stabilize community dynamics of symmetric species. Individual-based simulations of a symmetric model with initially 80 identical species, simulated on an area of 200 ha without immigration for 5000 time steps (25,000 years). Different colours correspond to different species. **left**, Recruits were randomly distributed, the dynamics is unstable with 2 extinctions. **middle**, Recruit were mostly scattered around conspecific adults, the dynamics is unstable with 7 extinctions. **right**, recruits were mostly scattered around random cluster centre, the dynamics is stable without extinctions. **a –c**, Species abundances with the bold black line indicating the expected mean abundance J^*^/80. **d–f**, Intraspecific pattern *k*_*ff*_. **g–i**, Interspecific pattern *k*_*f*h_ of heterospecifics with respect to the foal species *f*. **j–l**, the mean relative interaction strength *B*_*f*_ of a heterospecific neighbour of an individual of species *f*. **m–o**, Stabilization, being the population-level heterospecific interaction strength relative to the corresponding conspecific interaction strength (that is, *α*_*f*h_/*α*_*ff*_). Note the different scale of the y-axes. Spatial patterns were measured at a 10 m neighbourhood.

**Extended Data Fig. 6:**
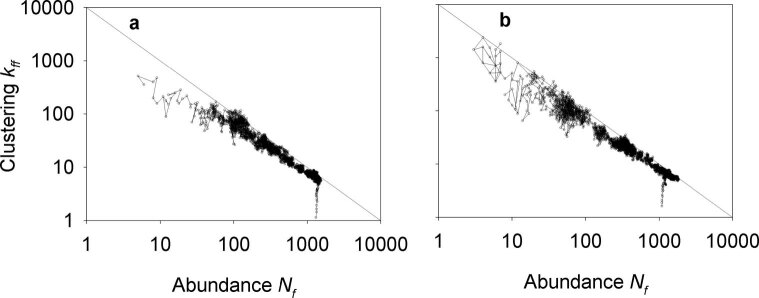
The relationship between clustering and abundance. Shown are detailed results of the simulation of Extended Data Fig. 5b for two species that went extinct. **a**, Dynamics of the relationship between abundance *N*_*f*_(*t*) and clustering *k*_*ff*_ for species 1. **b**, same for species 3. The solid line indicates the power law *k*_*ff*_ = 10,000/*N*_*f*_(*t*).

Indeed, the way recruits were placed was critical for coexistence. Randomly placed recruits produced unstable dynamics (Extended Data Fig. 5a) characterized by regularity (mean of *k*_*ff*_ = 0.92) and segregation (mean of *k*_*f*h_ = 0.92), both caused by competition^18^, and the instability was caused by con- and heterospecifics competing equally at the population level (that is, *α*_*f*h_/*α*_*ff*_ ≈ 1) (Extended Data Fig. 5d,g). When we followed the common approach of placing recruits with a kernel around conspecific adults^17,18,35–39^ to mimic dispersal limitation, we again found unstable dynamics (Extended Data Fig. 5b), despite intraspecific clustering and interspecific segregation (that is, *α*_*f*h_/*α*_*ff*_ < 1; Extended Data Fig. 5n). The reason for the instability was high clustering of rare species^24,35^ (Extended Data Fig. 6) that completely negated the potentially positive effects of *α*_*f*h_/*α*_*ff*_ < 1.

In contrast, community dynamics can be stabilized if recruits are placed in small clusters but independent of the location of conspecific adults (Extended Data Fig. 5c). With this mechanism we mimic canopy gaps^40^, animal seed dispersal^41^ or other mechanisms that can generate clustering independent of parent locations, as found at BCI^42^. Decoupling clustering from the parent locations does not lead to the negative relationship between clustering and abundance, and all measures of spatial patterns converged quickly into quasi-equilibrium (Extended Data Fig. 5f,i,o).

**Extended Data Fig. 7:**
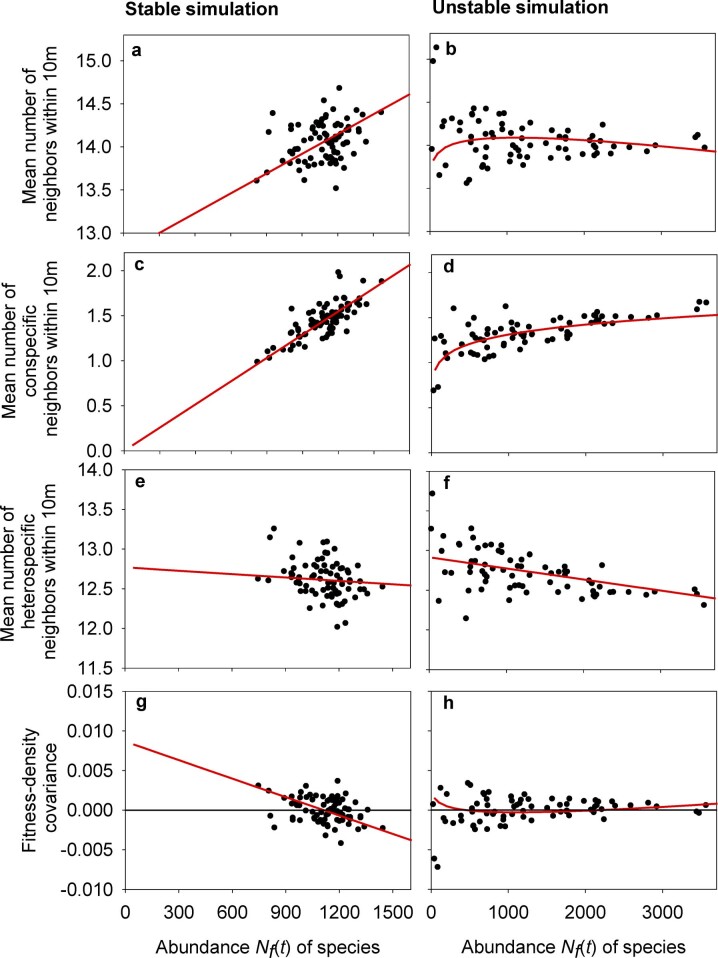
Mechanism underlying the fitness-density covariance in our simulation model. Quantities involved in the estimation of the fitness–density covariance (equation 21) for model simulations of the stable scenario (**left**) and the unstable scenario (**right)**. The data were taken from time step 5000 of the simulations shown in Extended Data Fig. 5c (stable dynamics) and of Extended Data Fig. 5b (unstable dynamics). Panels show the mean number $\bar n_{ff} + \bar n_{fh}$ of all neighbours (**a**, **b**), of conspecific neighbours $\bar n_{ff}$ (**c**, **d**), of heterospecific neighbours $\bar n_{fh}$ (**e**, **f**), and the resulting fitness-density covariance $\tilde \lambda _f - \bar \lambda _f$ (**g**, **h**), of all species plotted over their abundance. The red lines show the expected relationship based on equations (21) and (22). We fitted power law relationships *k*_*ff*_(*N*_*f*_) = *a N*_*f*_^*b*^ to the data. For stable dynamics we find *k*_*ff*_(*N*_*f*_) = 8.2, *k*_*fh*_(*N*_*f*_) = 0.905 and for unstable dynamics *k*_*ff*_(*N*_*f*_) = 3723/*N*_*ff*_^0.883^ and *k*_*fh*_ (*N*_*f*_) = 0.890.

**Extended Data Fig. 8:**
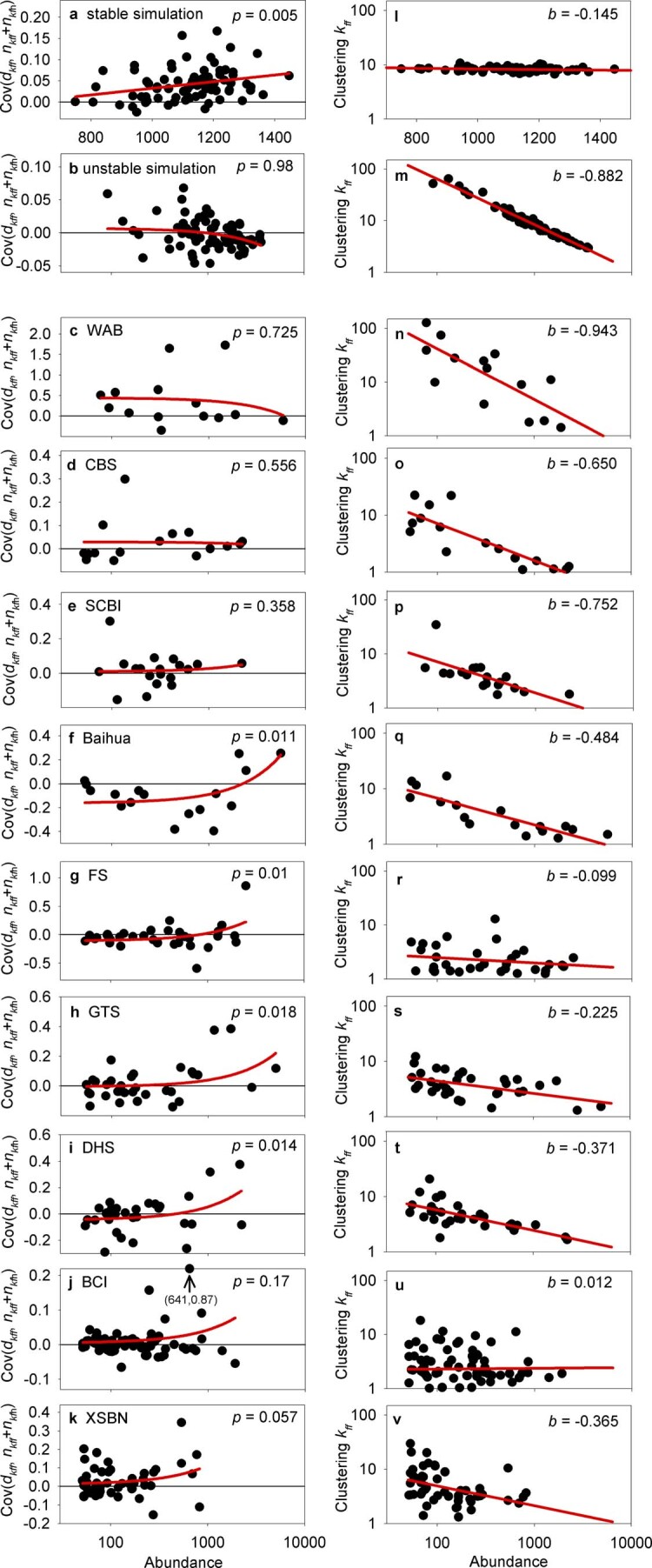
Rare species advantage and abundance −clustering relationships in model simulations and ForestGEO plots. The covariance between the local dominance *d*_*kff*_ of species *f* (that is, *d*_*kff*_ = *n*_*kff*_/(*n*_*kff*_ + *n*_*kf*h_)) and the total number of neighbours (that is, *n*_*kff*_ + *n*_*kf*h_). A positive relationship of the covariance with abundance indicates that, when a species becomes rare, areas of higher conspecific crowding have fewer competitors. Shown are results of model simulations (**a**, **b**) and the nine ForestGEO forest plots (**c**–**k**). The p-value is for the null hypothesis that the slope of the linear regression (red lines) is not positive. (**l–v**) The corresponding relationships between clustering *k*_*ff*_ and abundance *N*_*f*_. The value of *b* is the slope of the power law *k*_*ff*_(*N*_*f*_) = *a N*_*f*_^*b*^ (red line). We used for the analysis focal species *f* with more than 50 individual. For plot names see Supplementary Table 1, and for raw data and details of the linear regressions see Supplementary Data Table 1.

The simulation data reveal the spatial coexistence mechanism underlying the positive fitness–density covariance^34^ (Extended Data Figs. 7 and 8). We find that the emerging spatial patterns lead to a situation where individuals of a common species are more likely to be near more neighbours and tend therefore to experience stronger competition (Extended Data Fig. 7). While the number of heterospecific neighbours remains approximately constant, the number of conspecific neighbours decreases with decreasing abundance if clustering does not change with abundance (equation (4a)). However, if clustering increases with decreasing abundance, the rare-species advantage is weakened and the dynamics become unstable^24^.

The data of several ForestGEO forest plots were compatible with a positive fitness–density covariance (Extended Data Fig. 8f–i) as they show that, when a species becomes rare, areas of higher conspecific crowding tend to have fewer total competitors. Comparison of the results of the stable versus unstable simulation showed that even relatively weak tendencies in this relationship are sufficient to stabilize the dynamics (Extended Data Figs. 8a,b). However, this was not the case for three temperate forest plots where the power-law clustering–abundance relationship showed exponents of *b* < −0.5 (that is, species tended to have high clustering at low abundances), but the other plots showed *b* > −0.5 (Extended Data Fig. 8n–p).

The apparent contradiction with previous theoretical studies^18,24,35,37^ where intraspecific clustering and interspecific segregation could not stabilize community dynamics thus arises as a consequence of the assumption of placing recruits close to their parents. This finding has important consequences for ecological theory because it shows, in contrast to the prevalent view^36,43,44^, that spatial patterns alone can lead to coexistence of multiple species. This is even more important since the specific spatial patterns required for this coexistence mechanism also exist in real forests.

## Conclusions

Understanding the mechanisms that maintain high species diversity in communities such as tropical forests is at the core of ecological theory, but these mechanisms are not yet fully resolved. Here, we argue that spatial patterns may play an important role in species coexistence of high diversity plant communities^17^. To test this hypothesis, we introduced a multiscale framework that reveals how pattern-forming processes operating at the level of individuals translate into mesoscale spatial patterns and how those patterns influence macroscale community dynamics.

We showed that the population-level interaction coefficients *α*_*fi*_ can have, for a broad range of common circumstances, a simpler structure than the underlying individual-level interaction coefficients *β*_*fi*_. This simplicity, which emerged from spatially explicit species interactions^30^, allowed for an analytical treatment of equilibrium, feasibility and invasion conditions of the corresponding macroscale models (equations (8–10)). Inserting the emerging *α*_*fi*_ coefficients into macroscale community models (for example, equation (7); Supplementary Table 3) should, in principle, allow us to take advantage of macroscale theory^9,11–13,45^. However, our results also indicate that the population-level interaction coefficients may not be temporally constant as commonly assumed but depend on spatial patterns that may change with abundance. This is especially likely if recruitment is mainly located close to the parents.

It is also possible to expand our framework to take into account more detailed neighbourhood crowding indices that consider not only the number of neighboured trees of a given species but also their distance and size^19,21,26^. This requires redefinition of the quantities *k*_*ff*_ and *k*_*f*h_ that describe intraspecific clustering and interspecific segregation, respectively, but does not change the overall structure of our equations. A special strength of our approach is that the population-level interaction coefficients contain measures of spatial neighbourhood patterns that can be directly estimated from fully mapped forest plots^4^. Together with additional information, this may allow for estimating network structures as well as stability of the whole community.

Our analysis revealed that communities of competing species can show a stable mode where the mesoscale patterns converge quickly into quasi-equilibrium and an unstable mode where negative relationships between species clustering and abundance emerge (Extended Data Figs. 5 and 6). The two modes are governed by the way species clustering is generated: the well-known unstable mode is related to clustering of recruits around their parents^17,18,35–39^ whereas the stable mode is related to clustering in locations that are independent from the parent locations, due, for example, to animal seed dispersal^41^ or canopy gaps^40^. This result calls for a closer examination of the spatial relationship between the recruits and adults. Indeed, independent placement of recruits from conspecific large trees may not be unusual. For example, Getzin et al.^42^ found in detailed analyses of the BCI forest that recruits were for most species spatially independent of large conspecific trees. For the stable mode we could identify conditions for coexistence, and forthcoming work may extend to quantifying the ability of additional mechanisms such as niche differences^7,8^, habitat associations^46^, spatial and temporal relative nonlinearity^7,8^ and storage effects^7,8^ to alleviate the destabilizing increase of clustering if species become rare.

This study explicitly incorporates spatial patterns in theoretical models of plant communities and combines analytical theory with spatial simulations and field data analysis. Our finding that species with similar attributes may show stable coexistence has profound implications for ecological theory. Furthermore, the multiscale framework we propose here opens exciting new avenues to explain species coexistence through a spatial lens.

## Methods

### Study areas

Nine large forest dynamics plots of areas between 20 and 50 ha were used in the present study (Supplementary Table 1). The forest plots are part of the ForestGEO network^4^ and are situated in Asia and the Americas at locations ranging in latitude from 9.15° N to 45.55° N. Tree species richness among the plots ranges from 36 to 468. All free-standing individuals with diameter at breast height (dbh) ≥1 cm were mapped, size measured and identified. We focused our analysis here on individuals with dbh ≥ 10 cm (resulting in a sample size of 131,582 individuals) and focal species with more than 50 individuals (resulting in 289 species). The 10 cm size threshold excludes most of the saplings and enables comparisons with previous spatial analyses^20,35,47,48^. Shrub species were also excluded.

Some of our analyses require estimation of the ratio *β*_*fi*_/*β*_*ff*_ that describes the relative individual-level competitive effect^18^ of individuals of species *i* on an individual of the focal species *f*. We used for this purpose phylogenetic distances^49^ based on molecular data, given in Myr, that assume that functional traits are phylogenetically conserved^19,26,27^. In this case, close relatives are predicted to compete more strongly or to share more pests than distant relatives^26^. To obtain consistent measures among forest plots, phylogenetic similarities were scaled between 0 and 1, with conspecifics set to 1, and a similarity of 0 was assumed for a phylogenetic distance of 1,200 Myr, which was somewhat larger than the maximal observed distance (1,059 Myr). This was necessary to avoid discounting crowding effects from the most distantly related neighbours^26^.

### Observed spatial patterns at species-rich forests

Figure 1 and Supplementary Data Table 1 show the intraspecific variation in our three crowding indices *n*_*kff*_, *n*_*kf*h_ and *n*_*kfβ*_ that can be approximated by gamma distributions. To assess how well the gamma distribution described the observed distribution, we used an error index defined as the sum of the absolute differences of the two cumulative distributions divided by the number of bins (spanning the two distributions). The maximal value of the error index is one, and a smaller value indicates a better fit.

Equations (6, 8 and 9) relate the measures of the emerging spatial patterns (that is, *k*_*ff*_, *k*_*f*h_ and *B*_*f*_) to macroscale properties and conditions for species coexistence. Even though our multiscale model (equation (7)) is simplified, it allows for a direct comparison with the emerging patterns in our nine fully stem-mapped forest plots. We estimate the key quantities of equations (8) and (9) directly from the forest plot data (Fig. 4), with the exception of the carrying capacities *K*_*f*_, which were indirectly estimated from the observed species abundances (assuming approximate equilibrium; equation (8) and Supplementary Data Table 1). This allowed us to estimate the feasibility index *µ*_*f*_ (equation (9)). Because statistical analyses with individual-based neighbourhood models^19,26^ based on neighbourhood crowding indices have shown that the performance of trees depends on their neighbours for *R* between 10 and 15 m, we estimate all measures of spatial neighbourhood patterns with a neighbourhood radius of *R* = 10 m. Analyses with *R* = 15 or *R* = 20 gave similar results.

### The spatial multispecies model and equilibrium

We use a general macroscale model to describe the dynamics of a community of *S* species:

11 $$\frac{{N_f\left( {t + {\Delta}t} \right) - N_f\left( t \right)}}{{{\Delta}t}} = N_f\left( t \right)\left[ {\left( {r_f - 1} \right) + s_f\exp \left( { - \alpha _{ff}N_f\left( t \right) - \mathop {\sum }\limits_{i \ne f} \alpha _{fi}N_i(t)} \right)} \right]$$

where *r*_*f*_ is the mean number of recruits per adult of species *f* within time step Δ*t*, *s*_*f*_ is a density-independent background survival rate of species *f* and the *α*_*fi*_ are the population-level interaction coefficients, yielding *α*_*ff*_ = *c* *γ*_*ff*_ *k*_*ff*_ *β*_*ff*_ and *α*_*fi*_ = *c* *γ*_*fβ*_ *k*_*f*h_ *β*_*ff*_ *B*_*f*_ (equation (6)). The *β*_*fi*_ are the assumed individual-level interaction coefficients between individuals of species *i* and *f*; *k*_*ff*_ = *K*_*ff*_(*R*) / π *R*^2^ and *k*_*f*h_ *=* *K*_*f*h_(*R*) / π *R*^2^ measure intraspecific clustering and interspecific segregation, respectively, with *K*_*ff*_(*R*) being the univariate *K* function for species *f* and *K*_*f*h_(*R*) the bivariate *K* function describing the pattern of all heterospecifics ‘h’ around individuals of species *f*. *A* is the area of the observation window.

Following equation (5), *B*_*f*_ can be estimated as

12 $$B_f = \frac{{\bar n_{f\beta }}}{{\bar n_{f{\mathrm{h}}}}} = \frac{{\mathop {\sum }\nolimits_{i \ne f} \left[ {ck_{fi}N_i\left( t \right)} \right]\frac{{\beta _{fi}}}{{\beta _{ff}}}}}{{\mathop {\sum }\nolimits_{i \ne f} \left[ {ck_{fi}N_i\left( t \right)} \right]}} = \frac{{\mathop {\sum }\nolimits_{i \ne f} k_{fi}N_i\left( t \right)\frac{{\beta _{fi}}}{{\beta _{ff}}}}}{{k_{f{\mathrm{h}}}\mathop {\sum }\nolimits_{i \ne f} N_i\left( t \right)}},$$

and is the weighted average of the relative individual-level interaction coefficients *β*_*fi*_/*β*_*ff*_ between species *i* and the focal species *f*, weighted by the mean number of individuals of species *i* in the neighbourhoods of the individuals of the focal species (that is, *c* *k*_*fi*_ *N*_*i*_(*t*)). For competitive interactions, *B*_*f*_ ranges between zero and one; *B*_*f*_ = 1 indicates that heterospecific and conspecific neighbours compete equally, and smaller values of *B*_*f*_ indicate reduced competition with heterospecific neighbours. The denominator can be rewritten in terms of segregation *k*_*f*h_ to all heterospecifics and the total number of heterospecifics ∑_*i*≠*f*_ *N*_*i*_(*t*).

The analytical expression of the equilibrium (equation (8)) relies on the assumption that the values of *B*_*f*_ are approximately constant in time. This assumption may not apply in our model during the initial burn-in phase of the simulations if the *β*_*fi*_/*β*_*ff*_ show large intraspecific variability (Supplementary Text and Figs. 1–5). The underlying mechanism is the central niche effect introduced by Stump^45^ where a species has reduced average fitness if it has high niche overlap with competitors.

Finally, the factors *γ*_*ff*_ = ln(1 + *b*_*ff*_ *β*_*ff*_) (*b*_*ff*_ *β*_*ff*_)^−1^ and *γ*_*fβ*_ = ln(1+ *b*_*fβ*_ *β*_*ff*_) (*b*_*fβ*_ *β*_*ff*_)^−1^ describe the influence of the variance-to-mean ratios *b*_*ff*_ and *b*_*fβ*_ of the gamma distribution of the crowding indices *n*_*kff*_ and *n*_*kfβ*_, respectively. For high survival rates during one time step (for example, >85%), the values of *γ*_*ff*_ and *γ*_*fβ*_ are close to one; in this case the exponential function in equation (1a) can be approximated by its linear expansion and *γ*_*ff*_ = *γ*_*fβ*_ = 1.

In equilibrium we have (*N*_*f*_(*t* + Δ*t*) ‒ *N*_*f*_(*t*))/Δ*t* = 0, which leads, with equation (7), to:

13 $$N_f^{\ast} = \left( {K_f - \frac{{\alpha _{f{\mathrm{h}}}}}{{\alpha _{ff}}}J^{\ast} } \right) \left( {1 - \frac{{\alpha _{f{\mathrm{h}}}}}{{\alpha _{ff}}}} \right)^{-1}$$

with $K_f = - {\mathrm{ln}}\left( {\frac{{1 - r_f}}{{s_f}}} \right) \left( \alpha _{ff} \right)^{-1}$ and the total number of individuals being $J^{\ast} = \sum _iN_i^{\ast}$. Rewriting equation (13) yields $\frac{K_f}{J^{\ast}} = \left( \frac{N_f^{\ast}}{J^{\ast}} \right) \left(1- \frac{\alpha_{f{\mathrm{h}}}}{\alpha_{ff}}\right) + \frac{\alpha_{f{\mathrm{h}}}}{\alpha_{ff}}$. For *α*_*f*h_/*α*_*ff*_ < 1 we therefore find *K*_*f*_ < J^*^, which indicates that a multispecies forest would host more individuals than a monoculture. To estimate J^*^ we sum equation (13) over all species *i* and find $J^{\ast} = \mathop {\sum }\limits_i \frac{{K_i}}{{1 - \alpha _{i{\mathrm{h}}}/\alpha _{ii}}} - J^{\ast} \mathop {\sum }\limits_i \frac{{\alpha _{i{\mathrm{h}}}/\alpha _{ii}}}{{1 - \alpha _{i{\mathrm{h}}}/\alpha _{ii}}}$. Therefore, we obtain

14 $$J^{\ast} = \mathop {\sum }\limits_{i = 1}^S \frac{{K_i}}{{1 - \alpha _{i{\mathrm{h}}}/\alpha _{ii}}} \left( {1 + \mathop {\sum }\limits_{i = 1}^S \frac{{\alpha _{ih}/\alpha _{ii}}}{{1 - \alpha _{ih}/\alpha _{ii}}}} \right)^{-1} = \frac{{Sm_K}}{{1 + Sm_\alpha }}$$

with $m_K = \frac{1}{S}\mathop {\sum }\limits_i^{\,} \frac{{K_i}}{{1 - \alpha _{i{\mathrm{h}}}/\alpha _{ii}}}$ and $m_\alpha = \frac{1}{S}\mathop {\sum }\limits_i^{\,} \frac{{\alpha _{i{\mathrm{h}}}/\alpha _{ii}}}{{1 - \alpha _{i{\mathrm{h}}}/\alpha _{ii}}}$ being averages over the *S* species of the community.

All species have positive abundances at equilibrium if the two conditions *µ*_*f*_ = *α*_*f*h_J^*^/*α*_*ff*_*K*_*f*_ < 1 and *α*_*f*h_/*α*_*ff*_ < 1 are met (see equation (9)). We now show that the chance that these conditions are satisfied for all species is larger if the values of *µ*_*f*_ show little intraspecific variability. To understand this, we assume that the *µ*_*f*_ can be approximated by their mean $\bar \mu$. In this case $J^{\ast} /\bar \mu$ is also approximately constant and we can replace *K*_*i*_ in equation (14) by $(J^{\ast} /\bar \mu ) (\alpha _{i{\mathrm{h}}}/\alpha _{ii})^{-1}$ and obtain

15 $$J^{\ast} = \mathop {\sum }\limits_{i = 1}^S \frac{{\left( {\frac{{J^{\ast} }}{{\bar \mu }}} \right)\frac{{\alpha _{i{\mathrm{h}}}}}{{\alpha _{ii}}}}}{{1 - \frac{{\alpha _{i{\mathrm{h}}}}}{{\alpha _{ii}}}}} \left( {1 + \mathop {\sum }\limits_{i = 1}^S \frac{{\frac{{\alpha _{i{\mathrm{h}}}}}{{\alpha _{ii}}}}}{{1 - \frac{{\alpha _{i{\mathrm{h}}}}}{{\alpha _{ii}}}}}} \right)^{-1} = \frac{{J^{\ast} }}{{\bar \mu }}\frac{{Sm_\alpha }}{{1 + Sm_\alpha }}$$

where *S* is the number of species in the community, and therefore

16 $$\bar \mu = \frac{{Sm_\alpha }}{{1 + Sm_\alpha }} < 1$$

Thus, in the case of a perfect interspecific balance in *µ*_*f*_ we always have a feasible equilibrium if *α*_*f*h_/*α*_*f*h_ < 1, and species can go extinct only if the intraspecific variability in *µ*_*f*_ becomes too large. The smaller the mean value of *µ*_*f*_, the more variability in *µ*_*f*_ is allowed. Equation (16) shows that $\bar \mu$ is smaller if the number *S* of species in the community is smaller and/or if the mean value of *m*_*α*_ is smaller.

Equation (16) also suggests that communities with more species need to show stronger species equivalence in *µ*_*f*_ because the term *S* *m*_*α*_(1 + *S* *m*_*α*_)^−1^ approaches a value of one for a large number of species *S*. This finding mirrors the results of analyses of Lotka–Volterra models with random interaction matrices^11^ that showed that the larger the number of species *S*, the more difficult it becomes to generate a feasible community.

### Invasion criterion

Using the population-level interaction coefficients (equation (6)) in the macroscale model, we now derive conditions for coexistence based on the invasion criterion^7,8^ for a species *m*. The growth rate of an invading species *m* with low density *M*(*t*) into the equilibrium community of all other *S* – 1 species *N*_*i*_(*t*) should be positive; thus, with equation (7), we have

17 $$\left( {r_m - 1} \right) + s_m \exp\left( { - \alpha _{mm}M\left( t \right) - \alpha _{m{\mathrm{h}}}\mathop {\sum }\limits_{i = 1}^{S - 1} N_i^{\ast} } \right) > 0.$$

Considering that $J_m^{\ast} = \mathop {\sum}\nolimits_{i = 1}^{S - 1} {N_i^{\ast}}$ and $\alpha_{mm} M(t) \ll \alpha_{f{\mathrm{h}}} J_m^{\ast}$ (that is, the invading species *m* is at low abundance and does not show strong clustering) we find $- \ln \left( \frac{1-r_m}{s_m} \right) > \alpha_{m{\mathrm{h}}} J_m^ \ast$, and by dividing by *α*_*mm*_ we obtain the invasion condition

18 $$\mu _m^i = \frac{{\alpha _{m{\mathrm{h}}}J_m^{\ast} }}{{\alpha _{mm}K_m}} < 1$$

which is basically identical to the condition for feasibility (equation (9)), but here the community size $J_m^ \ast$ of the reduced community appears instead of the equilibrium community size J^*^ of all species, including species *m*. Thus, a new species *m* is more likely to invade if it has a high value of the carrying capacity *K*_*m*_ and if it more strongly reduces heterospecific interactions relative to conspecific interaction (that is, *α*_*m*h_/*α*_*mm*_ is smaller). However, if the species is too efficient (that is, has too large a capacity *K*_*m*_ and/or too low an *α*_*m*h_/*α*_*mm*_) it may increase the value of J^*^ too much (equation (14)), thereby causing the extinction of the weakest species with the highest values of *µ*_*f*_ (that is, a too-low value of *K*_*m*_ and a too-high value of *α*_*f*h_/*α*_*ff*_). Equation (18) also suggest that an equilibrium with *µ*_*f*_ > 1 and *α*_*m*h_/*α*_*mm*_ > 1 will be unstable.

### Fitness–density covariance

To place our new spatial coexistence mechanism in the context of existing coexistence theory, we apply scale transition theory^34^ to our model version where spatial effects are the only potential coexistence mechanism (that is, all species have the same parameters and all individuals compete equally; *β*_*fi*_/*β*_*ff*_ = 1, *B*_*f*_ = 1).

Following equation (1a), the expected fitness of an individual *k* of a focal species *f* (that is, its expected contribution to the population after some defined interval of time Δ*t*) in the macroscale model (equation (7)) is

19 $$\lambda _{kf} = r_f + s_{f}f\left( {W_k} \right) = r_f + s_f \exp\left( { - \beta _{ff}\left( {n_{kff} + \mathop {\sum }\limits_{i \ne f} n_{kfi}} \right)} \right)$$

where *W*_*k*_ = *n*_*kff*_ + ∑_*i≠f*_*n*_*kfi*_ is the fitness factor of individual *k*, *f*(*W*_*k*_) = exp(−*β*_*ff*_  *W*_*k*_) is the fitness function and *n*_*kff*_ and ∑_*i≠f*_ *n*_*kfi*_ are the number of conspecific and heterospecific neighbours, respectively, of individual *k* within distance *R*. The spatial average of the fitness factor over the entire plot is

20 $$\bar W_k = cN_f\left( t \right) + c\mathop {\sum }\limits_{i \ne f} N_i(t) = cJ(t)$$

where *c* = π*R*^2^ / *A*, and *J*(*t*) = ∑_*i*_*N*_*i*_(*t*) is the total number of individuals in the plot. Given that *J*(*t*) converges very quickly into equilibrium J^*^ (Extended Data Fig. 5 and Supplementary Figs. 1 and 2), we find for the spatial average fitness $\bar \lambda _f = 1$.

The average individual fitness $\tilde \lambda _f(t)$ of a focal species *f* is the average of *λ*_*k,f*_ over all individuals *k* of species *f* and can be estimated for the macroscale model (equations (6 and 7)) as $\tilde \lambda _f\left( t \right) = N_f\left( {t + {\Delta}t} \right)/N_f\left( t \right)$. A key ingredient of scale transition theory^34^ is that the fitness–density covariance is given by ${\mathrm{cov}} = \tilde \lambda _f\left( t \right) - \bar \lambda _f$. With equation (3) and *γ*_*ff*_ ≈ *γ*_*fβ*_ ≈ 1 and $\bar n_{f\beta } = \bar n_{f{\mathrm{h}}}$ we find

21 $$\tilde \lambda _f\left( t \right) - \bar \lambda _f = (r_f - 1) + s_f \exp( - \beta _{ff}(\bar n_{ff} + \bar n_{f{\mathrm{h}}}))$$

where the mean of the crowding indices is given by $\bar n_{ff}(N_f) = ck_{ff}(N_f)N_f$ and $\bar n_{f{\mathrm{h}}}(N_f) = ck_{f{\mathrm{h}}}(J^{\ast} - N_f)$ (equation (4)). Therefore, if clustering *k*_*ff*_ and segregation *k*_*f*h_ are independent from abundance *N*_*f*_, more abundant species have more neighbours, since

22 $$\bar n_{ff} + \bar n_{f{\mathrm{h}}} = c(k_{ff} - k_{f{\mathrm{h}}})N_f + ck_{f{\mathrm{h}}}J^{\ast}$$

Thus, a positive fitness–density covariance in our model means that individuals of a common species are more likely to be near more trees in total.

Extended Data Fig. 7 shows the quantities $\bar n_{ff} + \bar n_{f{\mathrm{h}}}$, $\bar n_{ff}$, $\bar n_{f{\mathrm{h}}}$ and $\tilde \lambda _f - \bar \lambda _f$ plotted over abundance *N*_*t*_ for data generated by our spatially explicit simulation model for the scenarios of stable and unstable dynamics (Extended Data Fig. 5b,c). Indeed, the stable simulations show a positive fitness–density covariance, however, there is no such trend for the dynamics of the unstable community (Extended Data Fig. 7g,h).

Spatial patterns will act as positive fitness–density covariance if, when a species becomes rare, areas of high conspecific crowding have fewer competitors. We tested this for the data generated by our simulation model and for the nine forest plots (Extended Data Fig. 8). We could estimate for each focal species *f* the covariance between the number of conspecific neighbours (that is, *n*_*kff*_) and the total number of neighbours (that is, *n*_*kff*_ + *n*_*kf*h_) and demand that the covariance should be mostly positive and larger for more abundant species. However, since the quantity *n*_*kff*_ appears in this test on both sides, a positive covariance can be expected. To compensate for this artefact, we instead used the covariance between the local dominance of conspecifics in the neighbourhood of individuals *k* (that is, *d*_*kff*_ = *n*_*kff*_ (*n*_*kff*_ + *n*_*kf*h_)^−1^) and total number of neighbours (that is, *n*_*kff*_ + *n*_*kf*h_) (Extended Data Fig. 8).

### Spatially explicit simulation model

The model is a spatially explicit and stochastic implementation of the spatial multispecies model (equation (7)), similar to that of May et al.^35,37^ and Detto and Muller-Landau^17^, and simulates the dynamics of a community of *S* tree species in a given plot of a homogeneous environment (for example, 50 ha) in 5 yr time steps adapted to the ForestGEO census interval (Extended Data Fig. 5 and Supplementary Figs. 1 and 2). Only reproductive (adult) trees are considered, but size differences between them are not considered. During a given time step the model first simulates stochastic recruitment of reproductive trees and placement of recruits, and second, stochastic survival of adults that depends on the neighbourhood crowding indices for conspecifics (*n*_*kff*_) and heterospecifics (*n*_*kfβ*_) (but excluding recruits). In the next time step, the recruits count as reproductive adults and are subject to mortality. No immigration from a metacommunity is considered. To avoid edge effects, torus geometry is assumed.

The survival probability of an adult *k* of species *f* is given by $s_f \exp \left(-\beta_{ff} \left( n_{kff}+n_{kf\beta}\right)\right)$ (equation (1a)). The two neighbourhood indices *n*_*kff*_ and *n*_*kfβ*_ describe the competitive neighbourhood of the focal individual *k* and sum up all conspecific and heterospecific neighbours, respectively, within distance *R,* but weight them with the relative individual-level interaction coefficients *β*_*fi*_/*β*_*ff*_ (refs. ^19,21,26^).

Each individual produces on average *r*_*f*_ recruits, and their locations are determined by a type of Thomas process^28^ to obtain clustering. To this end, the spatial position of the recruits is determined by two independent mechanisms. First, a proportion 1 – *p*_d_ of recruits is placed stochastically around randomly selected conspecific adults by using a two-dimensional kernel function (here a Gaussian with variance *σ*^2^). This is the most common way to generate species clustering in spatially explicit models^17,18,35–39^. Specifically, we first randomly select one parent for each of these recruits among the conspecific adults and then determine the position of the recruit by sampling from the kernel. Second, the remaining proportion *p*_d_ of recruits is distributed in the same way around randomly placed cluster centres that are located independently of conspecific adults. This mode mimics spatial clustering of recruits independent of the parent locations^42^ in a simple way, such as contagious seed dispersal by animals^50^ or forest gaps that may imprint clumped distributions of recruits of pioneer species^40^. For each species we assume a density *λ*_*f*c_ of randomly distributed cluster centres, which have, at each time step, a probability *p*_*f*p_ of changing location. For each of these recruits, we first randomly select one cluster centre among the cluster centres of the corresponding species and then determine the position of the recruit by sampling from the kernel. For the simulation shown in Extended Data Fig. 5a, the recruits were located at random positions within the plot.

### Parameterization of the simulation model

Extended Data Fig. 5 shows simulations of the individual-based model conducted in a 200 ha area containing approximately 83,000 trees with, initially, 80 species. There was no immigration. The model parameters were the same for all species, and all species followed exactly the same model rules. We selected *β*_*fi*_ = *β*_*ff*_ to obtain no differences in con- and heterospecific interactions and *s*_*f*_ = 1 (no background mortality), and we adjusted the parameters *β*_*ff*_ = 0.0075 and *r*_*f*_ = 0.1 to yield tree densities (415 ha^−1^) and an overall 5 yr mortality rate (10%) similar to those of trees with dbh ≥ 10 cm in the BCI plot^51^.

The Gaussian kernel used to place recruits around conspecific adults or around random cluster centres had a parameter *σ* = 10 m. There were 40 random cluster centres in total for each species that had a probability of *p*_*f*p_ = 0.3 of changing location within one census interval. The only difference between the simulation shown in Extended Data Fig. 5b and the one shown in Extended Data Fig. 5c is that in the former, we used a proportion *p*_d_ = 0.05 of recruits to be placed around randomly distributed cluster centres (that is, 95% of the recruits were placed close to their parents), but in the latter, we selected *p*_d_ = 0.95 (that is, 95% of the recruits were placed around randomly distributed cluster centres). In our simulations, on average, one of these cluster centres received four recruits per time step, which were scattered within a radius of approximately 30 m, and received approximately 13 recruits during its lifetime (at each time step it had a probability of 0.3 of changing location). In contrast, in Extended Data Fig. 5a recruits were placed at random locations within the plot.

### Reporting Summary

Further information on research design is available in the Nature Research Reporting Summary linked to this article.

## Supplementary information

Supplementary Information Supplementary Figs. 1–5, Tables 1–3 and text.

Reporting Summary

Peer Review Information

Supplementary Data Table 1 The table contains the different summary functions characterizing the spatial patterns of the 289 focal species and raw data for Figs. 2–4 and Extended Data Figs. 2–4 and 8.

Supplementary Software Source code of the simulation model written in Delphi (Pascal) that contains the procedures to repeat the results shown in Extended Data Figs. 5–7 and Supplementary Figs. 1–5 and to estimate the summary functions of spatial patterns. Rename the file to ‘NeutralModel.pas’ and use it as main unit. There is no graphical output.

## Data Availability

The data that support the findings in this manuscript (and the raw data for Figs. 2–4 and Extended Data Figs. 2–4 and 8) can be found in Supplementary Data Table 1. To generate this data, we used the raw census data of the ForestGEO network that can only be shared on request because most PIs have not made them publicly available. For data requests see https://forestgeo.si.edu/sites-all.

## Code availability

The source code of the simulation model is provided in the Supplementary Information.
