# Supplementary material for: Consequences of spatial patterns for coexistence in species-rich plant communities
Source: Nat Ecol Evol. 2021 May 3;5(7):965–73. doi: 10.1038/s41559-021-01440-0 (PMC8257505; doi:10.1038/s41559-021-01440-0)
Supplement: Supplementary file 1 — Supplementary Figs. 1–5, Tables 1–3 and text. [file 41559_2021_1440_MOESM1_ESM.pdf]

nature portfolio

https://doi.org/10.1038/s41559-021-01440-0

Supplementary information

Consequences of spatial patterns for coexistence in species-rich plant communities

In the format provided by the
authors and unedited

Supplementary Information

Consequences of spatial patterns for coexistence in species rich plant communities

Thorsten Wiegand, Xugao Wang, Kristina J. Anderson-Teixeira, Norm Bourg, Min Cao, Xiuqin Ci, Stuart J. Davies, Zhanqing Hao, Robert Howe, W. John Kress; Juyu Lian, Jie Li, Luxiang Lin, Yiching Lin, Keping Ma, William McShea, Xiangcheng Mi, Sheng-Hsin Su, I-Fang Sun, Amy Wolf, Wanhui Ye, Andreas Huth

Supplementary Table 1
Supplementary Table 2
Supplementary Table 3
Supplementary Text detailing testing of technical assumptions of the macroscale model.
Supplementary Figure 1
Supplementary Figure 2
Supplementary Figure 3
Supplementary Figure 4
Supplementary Figure 5

Supplementary Data Table (separate Excel file)
Source code of simulation model (separate ASCII text file)

Corresponding authors: thorsten.wiegand@ufz.de and wangxg@iae.ac.cn

Supplementary Table 1: Characteristics of the selected forest plots.

Plot†	WAB	CBS	Baihua	SCBI	FS	GTS	DHS	BCI	XSBN
Latitude	45.55	42.4	34.16	38.89	24.76	29.25	23.17	9.15	21.61
Longitude	-88.80	128.08	106.15	-78.15	121.56	118.12	112.51	-79.85	101.57
Elevation (m)	483-514	791-808	780-802	330	600-733	446-715	230-470	120-155	709-869
Total species richness	36	52	63	65	110	159	210	304	468
Individuals ≥10cm dbh	14021	9995	17197	8269	19261	18215	11906	21456	12263
No. species ≥10cm dbh	22	34	34	49	77	109	131	238	339
No. focal species	14	16	17	19	33	33	30	75	52
Forest type	temperate	temperate	temperate	temperate	subtropical	subtropical	subtropical	tropical	tropical
Basal area (m^2/ha)	27.7	43.1	32.3	33.3	38.2	36.9	30.1	31	40.7
Area (ha)	25.2	25	24	25.6	25	24	20	50	20
Dimensions	(300 × 840m)	(500 × 500m)	(600 × 400m)	(400 × 640m)	(500 × 500m)	(600 × 400m)	(400 × 500m	(1000 × 500m)	(400 × 500m)
Mean T (°C)	4	2.9	3.6	12.8	18.2	15.3	20.9	27	21.8
Mean P (mm)	805	700	700	944	4067	1963	1985	2551	1493

†WAB: Wabikon, CBS: Changbaishan, Baihua: Baihua, SCBI: Smithsonian Conservation Biology Institute, FS: Fushan, GTS: Gutianshan, DHS: Dinghushan, BCI: Barro Colorado Island, XSBN: Xishuangbanna.

Supplementary Table 2: Explanation of mathematical symbols

Symbol	Meaning
**(A) Population level quantities**	
$N_f(t)$, N^*_f	abundance of species *f* at time *t* and corresponding equilibrium abundance.
$J^* = \sum_f N^*_f$	total community size in equilibrium (eq. 14).
Δt	time step, in our case 5 years, the census interval of the ForestGEO plots.
r_f	average number of offspring of an individual of species *f* within the 5 year census interval.
s_f	background per capita survival rate of species *f* in absence of neighbourhood competition within the 5 year census interval.
β_{fi}	individual-level interaction coefficient, measures the negative impact an individual of species *i* has on survival of individuals of the focal species *f*.
β_{fi}/β_{ff}	relative individual-level interaction coefficient, ranges between zero and one. We use phylogenetic similarity as surrogate for β_{fi}/β_{ff}. Large similarity (i.e., $\beta_{fi}/\beta_{ff} \approx 1$ leads to strong competition whereas low similarity ($\beta_{fi}/\beta_{ff} << 1$) leads to weak competition.
α_{fi}	population-level interaction coefficient, measures in the macroscale multispecies model of equation 7 the negative impact an individual of species *i* has on survival of individuals of the focal species *f*.
A	area of the observation window.
f	focal species.
**(B) quantities for neighbourhood crowding indices**	
R	neighbourhood radius: all individuals within distance *R* of a focal individual are counted, we use R = 10m.
k	the focal individual.
n_{kff}	conspecific crowding index: number of conspecific neighbours within distance *R* of individual *k* of the focal species *f* (Fig. 1a).
n_{kfi}	pairwise crowding index: number of neighbours of species *i* within distance *R* of individual *k* of the focal species *f*.
$n_{kf\text{h}} = \sum_{f \neq i} n_{kfi}$	heterospecific crowding index: number of heterospecific neighbours within distance *R* of individuals *k* of the focal species *f*. The subscript "h" refers to all heterospecifics neighbours (Fig. 1b).
$n_{kf\beta} = \sum_{f \neq i} (\beta_{fi}/\beta_{ff}) n_{ki}$	interaction crowding index: as $n_{kf\text{h}}$, but weights neighbours of species *i* with the relative interaction coefficients β_{fi}/β_{ff} (Fig. 1b).
$\bar{n}_{kf}$, $\bar{n}_{k\text{h}}$, $\bar{n}_{k\beta}$	the mean of the intraspecific distributions of n_{kff}, $n_{kf\text{h}}$, and $n_{kf\beta}$, respectively.

b_{ff}, $b_{f\text{h}}$, $b_{f\text{ß}}$	the variance-to-mean ratio of the intraspecific distributions of n_{kff}, $n_{kf\text{h}}$, and $n_{kf\text{ß}}$, respectively.
γ_{ff}, $\gamma_{f\text{h}}$, $\gamma_{f\text{ß}}$	characterize the shape of the distributions of n_{kff}, $n_{kf\text{h}}$, and $n_{kf\text{ß}}$, respectively, with $\gamma_s = \ln(1 + b_s)/b$ with subscripts s = *ff*, *f*h, or *f*ß.
$c = \pi R^2/A$	scales population sizes from the plot scale with area A to the neighbourhood scale with area πR^2.

(C) point pattern quantities

$k_{ff} = K_{ff}(R)/\pi R^2$	the K function taken over the interval 0 to R, divided by neighbourhood area πR^2. It quantifies clustering of the focal species f relative to the null expectations of randomly distributed conspecifics. Thus, $k_{ff} > 1$: clustering, $k_{ff} \approx$ random pattern, and for $k_{ff} < 1$: regularity.
$k_{f\text{h}} = k_{f\text{h}}(R)$	the bivariate K function, taken over the interval 0 to R and divided by neighbourhood area πR^2. It quantifies mean excess (or lack) of heterospecific neighbours (h) within distance R of the typical individual of the focal species (f) relative to the null expectations of independently distributed heterospecifics. Thus, $k_{f\text{h}} > 1$: attraction, $k_{f\text{h}} \approx 1$: independence, and $k_{f\text{h}} < 1$: segregation.
$B_f = \bar{n}_{f\beta} / \bar{n}_{f\text{h}}$	the average (relative) heterospecific neighbourhood competition strength suffered by the typical individual of species f from one heterospecific individual within its neighbourhood with radius R (eqn. 12). It indicates how much the competition strength of one heterospecific neighbour differs on average from that of one conspecific neighbour.

(D) additional model variables

$K_f = -\ln(\frac{1-r_f}{s_f}) / \alpha_{ff}$	the carrying capacity of species f for the macroscale model of equation 7; its equilibrium without competitors. See Supplementary Table 3 for K_f resulting from alternative macroscale models.
$\mu_f = \frac{\alpha_{f\text{h}}}{\alpha_{ff}} \frac{J^*}{K_f}$	feasibility index, must be < 1 for all species to get a feasible community (i.e., where all species abundances are positive).

Supplementary Table 3: Results of alternative macroscale models.

The population-level interaction coefficients α_{fi} (Eqn. 6) resulting from our upscaling analysis can be inserted into different macroscale community model with linear or negative exponential fitness functions $f(W_k)$. After redefinition of the carrying capacity K_f the equilibrium (Eqn. 8), the feasibility condition (Eqn. 9) and the invasion criterion (Eqn. 18) presented in the main text apply also for different macroscale models. The table lists a few of them together with the corresponding carrying capacities. In all models (except the annual plant model) the fitness factor is given by $W_f = \alpha_{ff}N_f + \alpha_{fh} \sum_{i\neq f} \alpha_{fi}N_i$ where the fitness functions $f(W_k) = \exp(-W_f)$ or $f(W_k) = (1 - W_f)$ describe the effect of density dependence with N_i being the abundance of species i, r_f and s_f are parameters for reproduction and survival, respectively and $s_f = 1 - m_f$ where m_f is the mortality rate. In the slightly modified annual plant model of Kraft et. al[14] [we describe density dependence by $\exp(-W'_f)$ instead of $1/(1+ W'_f)$] the germination rate, seed survival rate, and per germinant fecundity of species f are g_f, s_f, and λ_f, respectively, and α_{fi} describes the per capita effect of species i on f.

Macroscale model	Carrying capacity K_f	Comment
$\frac{1}{N_f}\frac{\Delta N_f}{\Delta t} = (r_f - 1) + s_f \exp(-W_f)$	$K_f = -\ln(\frac{1-r_f}{s_f}) / \alpha_{ff}$	our model (Eqn. 7) with density dependence in survival
$\frac{1}{N_f}\frac{\Delta N_f}{\Delta t} = (s_f - 1) + r_f \exp(-W_f)$	$K_f = -\ln(\frac{1-s_f}{r_f}) / \alpha_{ff}$	model with density dependence in recruitment
$\frac{1}{N_f}\frac{\Delta N_f}{\Delta t} = (r_f + s_f)\exp(-W_f) - 1$	$K_f = -\ln(\frac{1}{r_f + s_f}) / \alpha_{ff}$	model with density dependence in survival and recruitment
$\frac{1}{N_f}\frac{\Delta N_f}{\Delta t} = (r_f - 1) + s_f(1 - W_f)$	$K_f = (\frac{r_f - m_f}{s_f}) / \alpha_{ff}$	Lotka-Volterra model with density dependence in survival
$\frac{1}{N_f}\frac{\Delta N_f}{\Delta t} = (s_f - 1) + r_f(1 - W_f)$	$K_f = (\frac{r_f - m_f}{r_f}) / \alpha_{ff}$	Lotka-Volterra model with density dependence in recruitment
$\frac{1}{N_f}\frac{\Delta N_f}{\Delta t} = (r_f + s_f)(1 - W_f) - 1$	$K_f = (\frac{r_f - m_f}{r_f + s_f}) / \alpha_{ff}$	Lotka-Volterra model
$\frac{1}{N_f}\frac{\Delta N_f}{\Delta t} = (1 - g_f)s_f + \lambda_f g_f \exp(-W'_f) - 1$	$K_f = -\ln(\frac{1}{\eta_f}) / \alpha_{ff}$	The annual plant model[14] with $\eta_f = \frac{1-(1-g_f)s_f}{\lambda_f g_f}$ and $W'_f = \alpha_{ff} g_f N_f + \alpha_{fh} \sum_{i\neq f} g_i N_i$

Supplementary Text: Testing of technical assumptions of the macroscale

The simulation model uses the relative individual-level interaction coefficients β_{fi}/β_{ff} as input parameters, but the population level interaction coefficients α_{fi}/α_{ff} and the mesoscale spatial patterns emerge from simulation of individual-level processes. The point process summary function B_f (i.e., the mean relative interaction strength of one heterospecific neighbour of an individual of species f) is an important quantity of the model that summarizes the emerging effects of the individual-level interaction coefficients β_{fi}/β_{ff} at the population-level. The analytical expression of the equilibrium (Eqn. 8) relies on the technical assumption that the values of B_f approach an equilibrium.

To check this assumption, we conducted simulations with the model where the relative individual-level interaction coefficients β_{fi}/β_{ff} were different from one. Thus, we introduced niche differences with respect to species interactions. To this end we repeated the simulations of the model where $\beta_{fi}/\beta_{ff} = 1$ (Extended Data Figure 5b, c) with two additional parameterizations of the interaction coefficients β_{fi}/β_{ff}. In Supplementary Figure 1 we used the phylogenetic dissimilarity $D^p{}_{fi}$ between the 34 focal species of the Fushan plot to derive the individual-level interaction coefficients as $\beta_{fi} = 1 - D^p{}_{fi}/1200$, and in Supplementary Figure 2 we determined the pairwise individual-level interaction coefficients β_{fi} of the 80 species based on a clumped spatial pattern of the 80 species locations within a two dimensional niche space[45] (shown as inset in Supplementary Figure 2j) with

$$\beta_{fi} = (1.1\ d_{\max} - d_{fi})/1.1\ d_{\max} \qquad 1$$

where d_{fi} was the Euclidean distance between the locations of species f and i in the niche space and $d_{\max}$ the maximal distance of all d_{fi}.

In all cases, the quantities B_f converged after a burn-in period quickly into equilibria (Supplementary Figures 1g,h and 2g,h). While the burn-in period was short when we used the phylogenetic dissimilarity of the Fushan plot to derive the individual-level interaction coefficients β_{fi}/β_{ff} (Supplementary Figure 1), the simulations with β_{fi}/β_{ff} derived from a two dimensional niche space showed more complex results (Supplementary Figure 2). To better understand these results, we apply concepts of a study by Stump[45] that classified phenomena occurring in case of nondiffuse competition in multispecies models.

Our simulation results show that the initial burn-in period is closely related to the **central niche effect** introduced in Stump[45] that states that a species has reduced average fitness if it has high niche overlap with several competitors (i.e., in our case if it is located in the centre of the two dimensional niche space). To show this we represented a species in the niche space by a circle proportionally to its abundance. In this analysis we used the two scenarios shown in Supplementary Figure 2: recruits scatter around conspecific adults (unstable dynamics) and recruits scatter around random cluster centres (stable dynamics). When doing this for different timesteps (i.e., $t = 20, 80, 320, 1280, 5120, 20480$) we find for both scenarios that species located in the centre of the niche space loose rapidly abundance and may even go extinct quickly in the unstable scenario (Supplementary Figures 3 and 4). However, the rapid re-

assembly due to the central niche effect occurred in the stable scenario only initially (Supplementary Figures 3 and 4), but afterwards the abundances approached equilibrium. Similarly, in the unstable scenario the rapid re-assembly due to the central niche effect occurred only initially, but the subsequent dynamics was driven by two additional effects introduced in Stump[45].

For the unstable scenario we observe that the **common competitor effect**[45] (i.e., if a species has high niche overlap with more common species, then its average fitness is reduced) comes into play after the burn-in phase when most "weak" species are extinct. Now the abundances of similar species (i.e., species located close to each other in the niche space) become increasingly dissimilar during time, and at the end only one species of a cluster of similar species may survive (Supplementary Figure 3). Here the destabilizing **community redistribution effect**[45] (i.e., when a species falls to low density, the relative abundance of its competitors will change, altering its ability to recover) is operating since the joined abundances of species within a cluster of similar species remains constant, but only one of them survives (Supplementary Figure 5).

These insights now inform us about the question if B_f will remain in approximation temporally constant. For the scenario of stable dynamics the values of B_f may shift only during the burn-in phase where the central niche effect leads to a rapid reassembly characterized by adjustments of species abundances. After this phase (in our case after some 1000 years), the B_f approach equilibria (Supplementary Figure 2h).

The scenario of unstable dynamics showed also during the burn-in phase a rapid reorganization of the community due to the central niche effect where species with too strong niche overlap lost rapidly abundance (Supplementary Figure 2g). Note that B_f varies in this parameterization initially over a very wide range with 10% and 90% percentiles of 0.49 and 0.74. During this phase we observe larger shifts in the values of B_f.

In the scenario of unstable dynamics B_f remains stable after the burn-in phase (Supplementary Figure 2g). This is because the common competitor effect does not impact B_f much since the "winner" species among several species with similar niches (and similar interaction coefficients β_{fi}) gains abundance on expense of the other species in the cluster (i.e., a destabilizing community redistribution effect; Supplementary Figure 5). Therefore changes in B_f, if any, are very slow after the burn-in period.

That means that the assumption of approximately constant B_f is only an issue during the burn-in phase when a community adjusts to initial conditions, for cases where rapid environmental change may force a re-assembly of the community, or if "superspecies" invade the community and force a reassembly. However, once the community has settled down in a quasi-stationary state, the assumption of a constant value of B_f is a reasonable assumption.

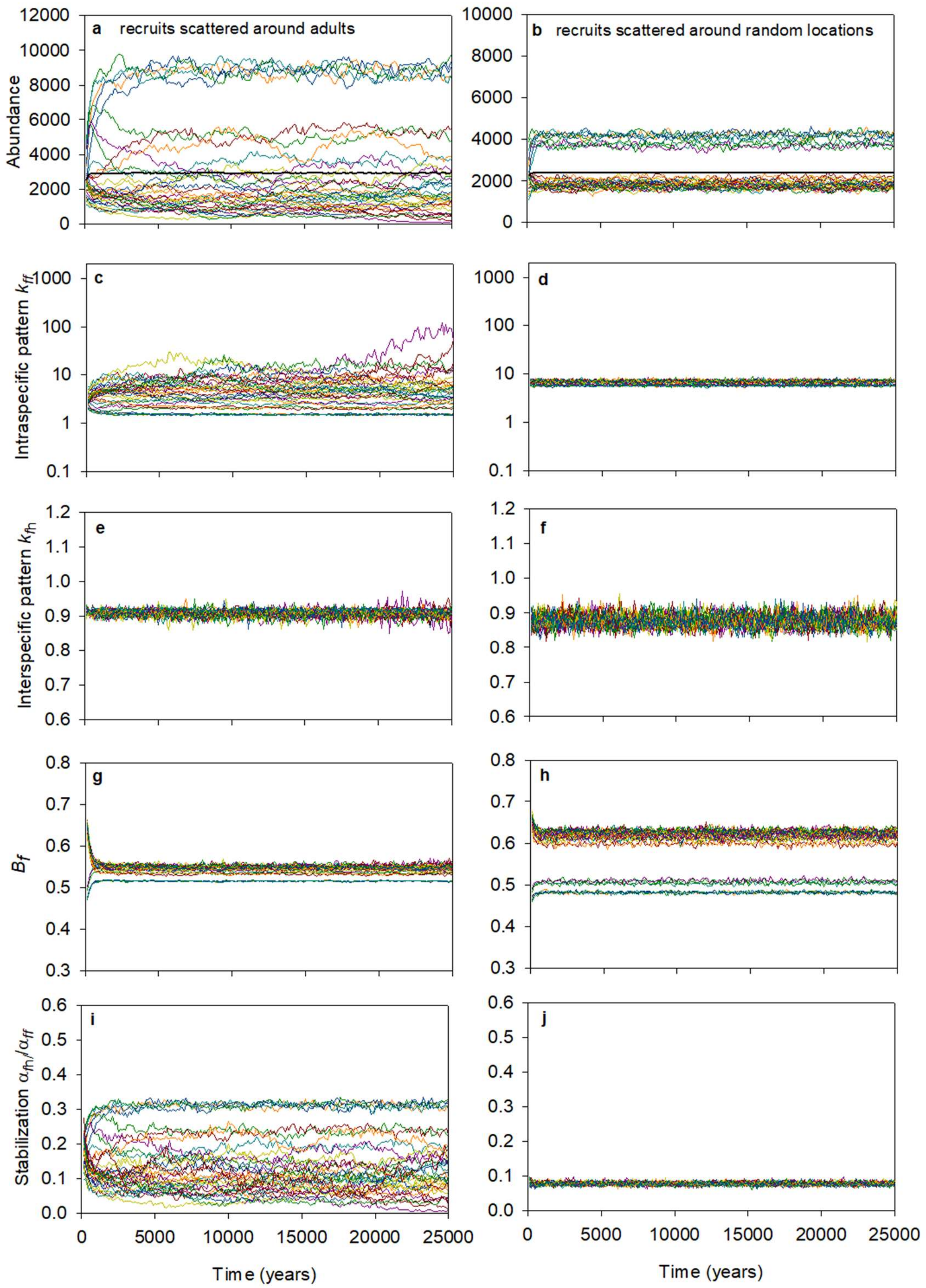

Supplementary Figure 1. Simulation experiment with individual-level interaction coefficients based on phylogenetic dissimilarity of the Fushan plot. Same as Extended Data Figure 5, but for communities with initially 34 species and complex individual level interaction coefficients based on the phylogenetic dissimilarity of the 34 focal species of the Fushan plot. All species survived.

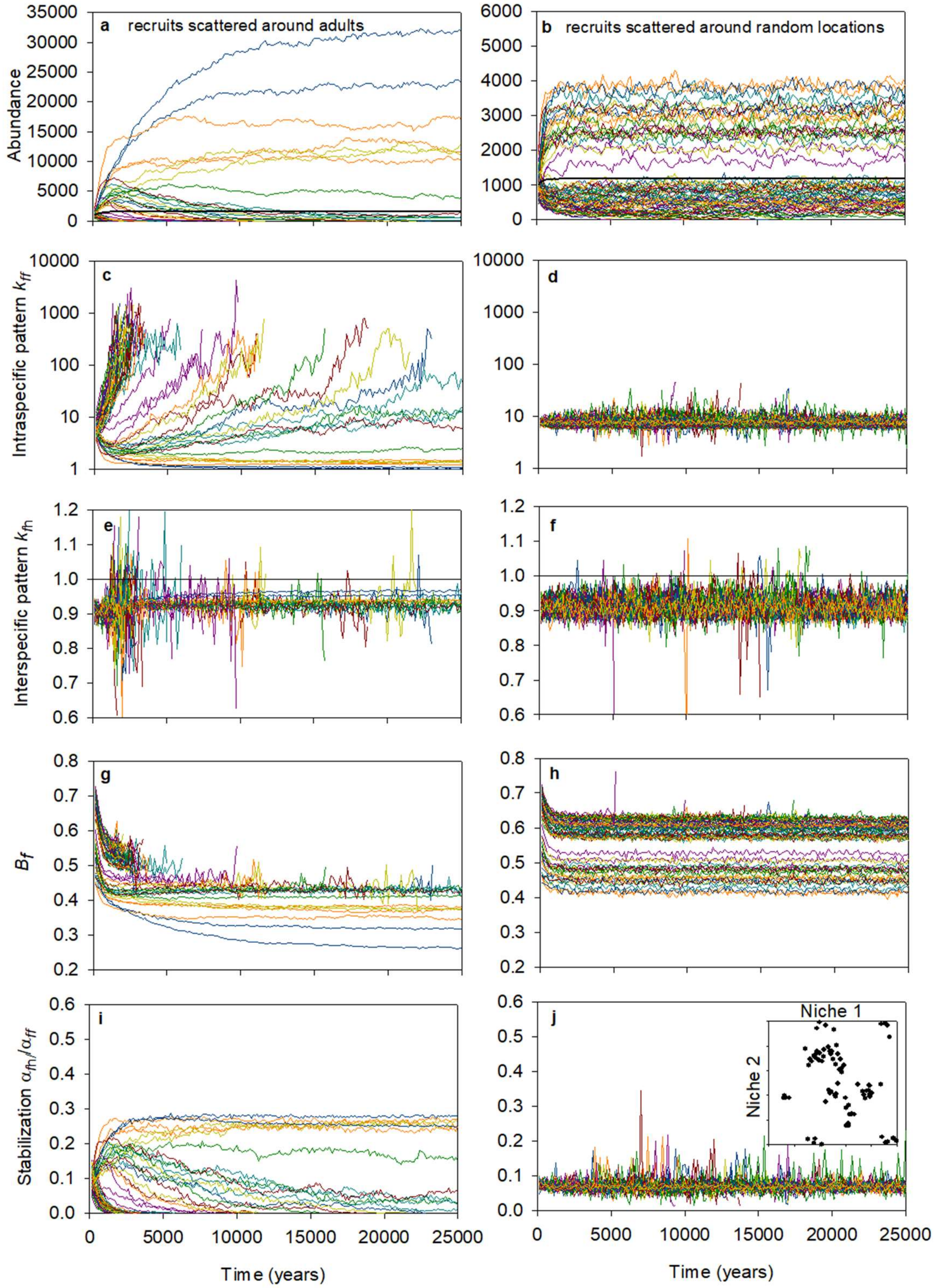

Supplementary Figure 2. Simulation experiment with individual-level interaction coefficients based on distance in a 2D trait space. Same as Extended Data Figure 5, but for communities with initially 80 species and individual-level interaction coefficients based on Euclidean distance between species locations in a two-dimensional niche space shown in the inset of panel j. 13 and 71 species survived in a and b, respectively.

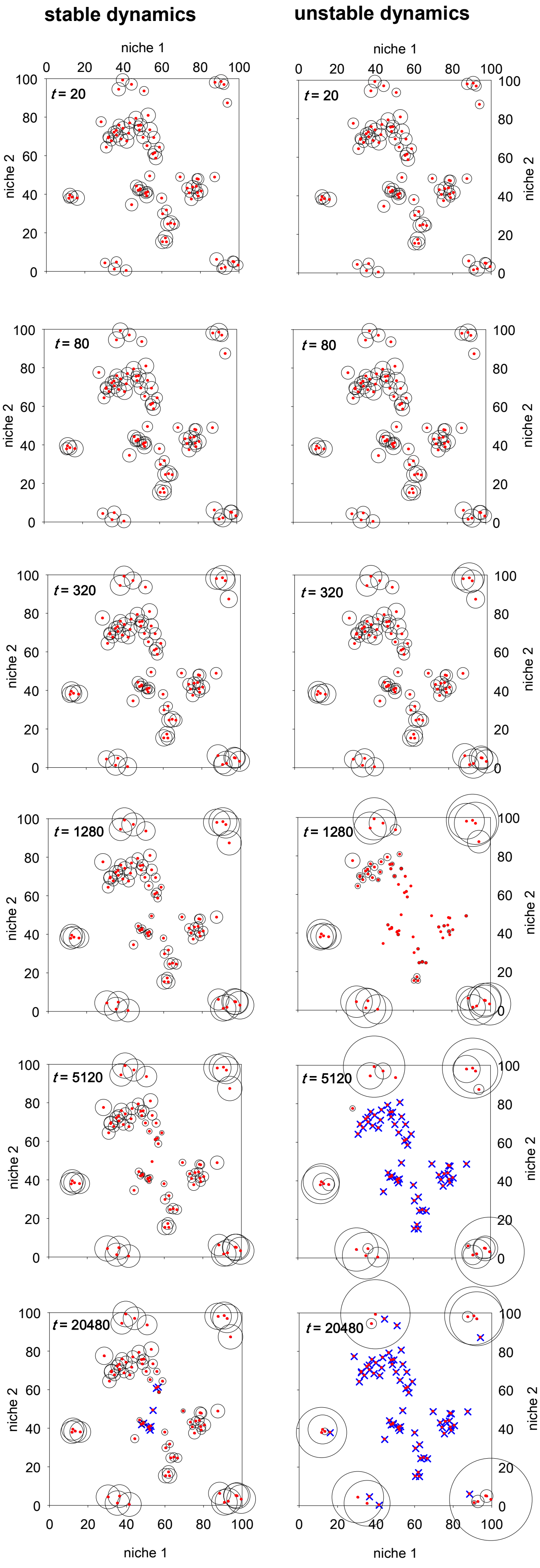

Supplementary Figure 3. Dynamics of the community, visualized in the niche space for the "stable" and the unstable scenario (shown in Supplementary Figure 2). We determined the matrix of the (relative) individual level interaction coefficients β_{fi}/β_f by representing each species as a point in two-dimensional niche space, and $\beta_{fi}/\beta_f = 1 - d_{fi}$ was proportional to the Euclidean distance d_{fi} between species f and i (as in Stump[45]). The area of the disks is proportional to abundance, red dots show the location of the 80 species, and blue crosses mark extinct species.

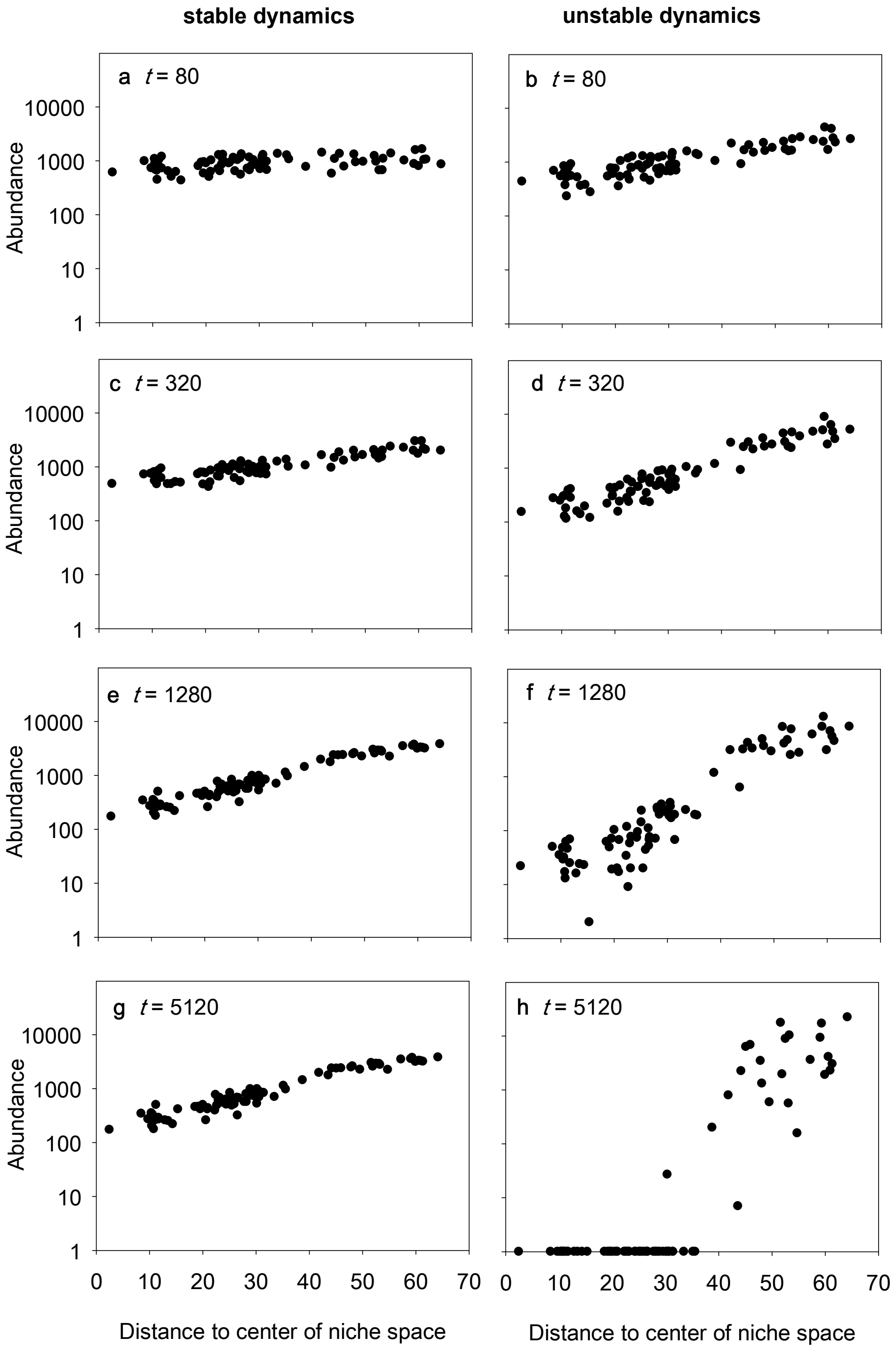

Supplementary Figure 4. Emergence of the central niche effect. We plotted for our model simulations shown in Supplementary Figure 2 abundance over distance to the centre of gravitation of the species in niche space. Indeed, the abundance increased strongly with distance from the centre. For the scenario of unstable dynamics, species with a distance below 40 went extinct very quickly (the extinct species are represented with an abundance of one).

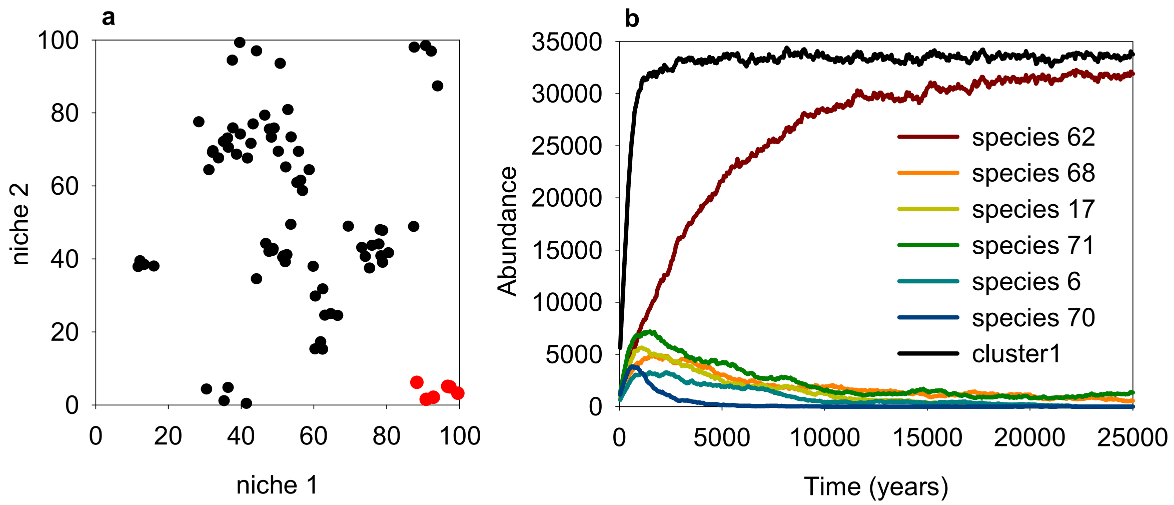

Supplementary Figure 5. Emergence of the community redistribution effect in the model simulations of unstable dynamics (shown in Supplementary Figure 2). (a) We analysed a clusters of similar species (i.e., species are located close to each other in niche space indicated by red colour), (b) time series of the abundances of individual species in the cluster and of the entire cluster. After the burn-in period, the joined abundances of species within the cluster remains constant, but the abundances of the component species change. Thus, we observe a destabilizing community redistribution effect where a species is replaced by a close competitor.
