# Supplementary material for: Consequences of spatial patterns for coexistence in species-rich plant communities
Source: Nat Ecol Evol. 2021 May 3;5(7):965–73. doi: 10.1038/s41559-021-01440-0 (PMC8257505; doi:10.1038/s41559-021-01440-0)
Supplement: Supplementary file 2 — Reporting Summary [file 41559_2021_1440_MOESM2_ESM.pdf]

nature research

Corresponding author(s): Thorsten Wiegand, Xugao Wang

Last updated by author(s): Feb 26, 2021

Reporting Summary

Nature Research wishes to improve the reproducibility of the work that we publish. This form provides structure for consistency and transparency in reporting. For further information on Nature Research policies, see our Editorial Policies and the Editorial Policy Checklist.

Statistics

For all statistical analyses, confirm that the following items are present in the figure legend, table legend, main text, or Methods section.

n/a	Confirmed	
☐	☒	The exact sample size (*n*) for each experimental group/condition, given as a discrete number and unit of measurement
☒	☐	A statement on whether measurements were taken from distinct samples or whether the same sample was measured repeatedly
☐	☒	The statistical test(s) used AND whether they are one- or two-sided<br>*Only common tests should be described solely by name; describe more complex techniques in the Methods section.*
☒	☐	A description of all covariates tested
☒	☐	A description of any assumptions or corrections, such as tests of normality and adjustment for multiple comparisons
☒	☐	A full description of the statistical parameters including central tendency (e.g. means) or other basic estimates (e.g. regression coefficient) AND variation (e.g. standard deviation) or associated estimates of uncertainty (e.g. confidence intervals)
☒	☐	For null hypothesis testing, the test statistic (e.g. *F*, *t*, *r*) with confidence intervals, effect sizes, degrees of freedom and *P* value noted<br>*Give P values as exact values whenever suitable.*
☒	☐	For Bayesian analysis, information on the choice of priors and Markov chain Monte Carlo settings
☒	☐	For hierarchical and complex designs, identification of the appropriate level for tests and full reporting of outcomes
☒	☐	Estimates of effect sizes (e.g. Cohen's *d*, Pearson's *r*), indicating how they were calculated

Our web collection on statistics for biologists contains articles on many of the points above.

Software and code

Policy information about availability of computer code

Data collection	The study did not involve field data collection. We wrote Pascal (Dephi 5.0) code for the simulation model to conduct the example simulations (results reported in Extended Figures 5, 6, 7, and Supplementary Figures S1-S5). The code of the simulation model is provided as supplementary file.
Data analysis	The code for estimating the crowding indices and other indices of spatial patterns from the ForestGeo data (results reported in Figs. 1, 2, Extended Data Figures 1, and 2) is included in the code of the simulation model. The same procedures can be used for simulated data and census data. The simple analyses for Figs. 2, 3, 4 and Extended Data Figures 2, 3, 4 and 8 are provided as Supplementary Data Table. This table contains also the raw data (including sample sizes) and estimation of the p-values shown in Extended Data Figure 8.

For manuscripts utilizing custom algorithms or software that are central to the research but not yet described in published literature, software must be made available to editors and reviewers. We strongly encourage code deposition in a community repository (e.g. GitHub). See the Nature Research guidelines for submitting code & software for further information.

Data

Policy information about availability of data

All manuscripts must include a data availability statement. This statement should provide the following information, where applicable:

- Accession codes, unique identifiers, or web links for publicly available datasets
- A list of figures that have associated raw data
- A description of any restrictions on data availability

The data that support the findings in this manuscript (and the raw data for figures 2, 3 and 4 and Extended Data Figures 2, 3, 4 and 8) can be found in the Supplementary Information as Data Table. To generate this data, we used the raw census data of the ForestGEO network that can only be shared on request because most PI's did not make them publicly available. For data request see https://forestgeo.si.edu/sites-all .

Field-specific reporting

Please select the one below that is the best fit for your research. If you are not sure, read the appropriate sections before making your selection.

☐ Life sciences ☐ Behavioural & social sciences ☒ Ecological, evolutionary & environmental sciences

For a reference copy of the document with all sections, see nature.com/documents/nr-reporting-summary-flat.pdf

Ecological, evolutionary & environmental sciences study design

All studies must disclose on these points even when the disclosure is negative.

Study description	This study develops a theoretical multiscale framework to reveal how pattern-forming processes operating at the level of individual trees translate into mesoscale spatial patterns and how they influence macroscale population and community dynamics. The approach includes analysis of mathematical and simulation models and comparison of the simulation results with data of fully-mapped forest plots of the Forest Global Earth Observatory (ForestGEO).
Research sample	We used ForestGeo (https://forestgeo.si.edu/explore-data) data sets of nine large forest dynamics plots of areas between 20 and 50 ha.
Sampling strategy	The nine forest plots included in the study have been completely censused for trees at least 10 cm in diameter, so there is no sampling within each forest.
Data collection	The study did not involve data collection. The data from model simulations was collected as described in the methods.
Timing and spatial scale	The study did not involve data collection. We used for each forest dynamics plot (size ranging between 20 and 50ha) data of one census.
Data exclusions	We used only trees with sizes >= 10cm dbh (diameter at breast height), which is an established approach in the field. This size threshold excludes most of the saplings and enables comparisons with previous spatial analyses. Tree species with a minimum of 50 individuals (dbh >= 10 cm) were used in the study to ensure that estimates of spatial patterns were reliable.
Reproducibility	The estimation of the measures of spatial patterns and the simulation model is specified exactly in the publication such that it could be reproduced. Additionally the simulation code (that includes estimation of measures of spatial patterns) is added as Supplementary File.
Randomization	Not applicable to this study.
Blinding	Blinding was not applicable to this study.

Did the study involve field work? ☐ Yes ☒ No

Reporting for specific materials, systems and methods

We require information from authors about some types of materials, experimental systems and methods used in many studies. Here, indicate whether each material, system or method listed is relevant to your study. If you are not sure if a list item applies to your research, read the appropriate section before selecting a response.

Materials & experimental systems

n/a	Involved in the study
☒	☐ Antibodies
☒	☐ Eukaryotic cell lines
☒	☐ Palaeontology and archaeology
☒	☐ Animals and other organisms
☒	☐ Human research participants
☒	☐ Clinical data
☒	☐ Dual use research of concern

Methods

n/a	Involved in the study
☒	☐ ChIP-seq
☒	☐ Flow cytometry
☒	☐ MRI-based neuroimaging
