# Supplementary material for: Consequences of spatial patterns for coexistence in species-rich plant communities
Source: Nat Ecol Evol. 2021 May 3;5(7):965–73. doi: 10.1038/s41559-021-01440-0 (PMC8257505; doi:10.1038/s41559-021-01440-0)
Supplement: Supplementary file 3 — Peer Review Information [file 41559_2021_1440_MOESM3_ESM.pdf]

naturerresearch

Peer Review Information

Journal: Nature Ecology & Evolution
Manuscript Title: Consequences of spatial patterns for coexistence in species rich plant communities
Corresponding author name(s): Thorsten Wiegand

Editorial Notes:

Reviewer Comments & Decisions:

Decision Letter, initial version:

27th May 2020

Dear Dr Wiegand,

Your Article entitled "Spatial coexistence theory shows that ecologically identical species can stably coexist" has now been seen by two reviewers, whose comments are attached. In the light of their advice, we have decided that we cannot offer to publish your manuscript in Nature Ecology & Evolution.

From the reports, you will see that while they find your work of some potential interest, the reviewers raise concerns about the advance your findings represent over earlier work and the strength of the novel conclusions that can be drawn at this stage. We feel that these criticisms are sufficiently important as to preclude publication of your work in Nature Ecology & Evolution.

I am sorry that we cannot be more positive on this occasion, but hope that you find the referees' comments helpful when preparing your paper for resubmission elsewhere.

[REDACTED]

****

Reviewer expertise:

Reviewer #1: modelling species coesxistence in tropical tree communities

Reviewer #2: mechanistic modelling of global and landscape scale plant communities

Reviewers Comments:

Reviewer #1 (Remarks to the Author):

Summary

How species coexist is a big question in ecology, and we don't have a simple, universal mechanism. The authors think we should (at least for tree communities, lines 70-74), and they think that the mechanism is intraspecific clustering (which they claim coexistence theory doesn't consider, line 75). The authors develop a fairly simple model of population dynamics, where a species' probability of mortality increases in the presence of others (where "the presence of others" is a combination of clustering and interspecific/intraspecific competition). They derive conditions for coexistence. Then, using data from 9 forest dynamics plots, the authors attempt to quantify some of the important parameters. Their data suggests that the species in 8 of these 9 plots are coexisting (with the exception being BCI). They also construct an individual based model, where they show that intraspecific clustering away from the parent can promote coexistence.

Overview

I think it paper should be rejected. Basically, the work is incorrect at times, I feel the authors are overselling their results, and many of the key ideas are not novel. I have made a list of my six biggest concerns (and two smaller concerns) below.

Concern 1: Intraspecific clustering not novel

A number of models have previously shown than intraspecific clustering can promote coexistence (Ives 1988, Chesson 2000). It is covered by coexistence theory, it is a form of fitness-density covariance. I've never heard this idea applied to plants, which is moderately novel, but I don't think it is Nature Ecology & Evolution-worthy novel. It has also been known for a really long time that most species show some degree of intraspecific clustering (Hubbell 1979).

Concern 2: The model has 2 mechanisms promoting coexistence, and they only acknowledge the second one

In every model of stable coexistence, intraspecific competition has to somehow be stronger than interspecific coexistence. Their model has two terms that can produce this effect: D (segregation/aggregation) and B (relative competitive effect). D is the novel mechanism here, and B is simply a very direct for of interspecific competition being stronger than interspecific competition (i.e. classic conspecific negative density dependence). They do not really focus on B at all, which is somewhat okay, except that it means that coexistence may not be driven by D. And, I think that B is driving some of their empirical results (explained below).

Concern 3: Multiple mechanisms can generate intraspecific clustering, and not of them all promote coexistence

For intraspecific clustering to promote coexistence, it is not enough that individuals are clustered. Instead, there needs to be something about the environment that causes individuals of a species to cluster in one place and not the other see (Chesson 2000). If conspecifics are clustered just because offspring tend to be dropped in groups, you will not get coexistence (this is basically why the authors didn't get coexistence when clustering was caused by seeds falling near their parent). Basically, if B=1 for all species (i.e. if species are coexisting because of clustering, rather than classical niche

partitioning), then what you need to get coexistence is a situation where individuals of a common species are more likely to be near more trees in total; it doesn't work if every tree is near 10 other trees, and clustering just means that many of those are conspecifics.

I bring this up because I don't think the authors distinguished between the different types of clustering. I'll be honest, I had a lot of trouble understanding how the authors calculated the k variables, so maybe I just misunderstood them (in which case the authors can explain why I am wrong). They showed that, indeed, a given individual is more likely to have a conspecific neighbor than expected by random. However, it is not clear that it is caused by a process that promotes coexistence (rather than, say, clustering caused by dispersal limitation).

Their data in fig. 1 does not convince me that I am wrong. Specifically, although they fit a Gamma distribution to the number of nearby neighbors, I'd argue that it looks a lot like a Poisson distribution too. This makes me think that the number of seeds at a spot is somewhat random. So, here is an explanation of what I mean: If we assume that a species has a global frequency of f, and has a mean of N neighbors, then we could model the number of neighbors as a Poisson distribution P(), where it has P(Nf) conspecific neighbors and P(N(1-f)) heterospecific neighbors. Here, you'd probably find some clustering, as some sites would have more neighbors than others. However, when the species becomes rare, it would still have an average of N neighbors, it's just that it would change from conspecifics to heterospecifics (which, if B was the same, wouldn't matter). You could also impose some degree of clustering (like, say, each individual had P((N-3)f + 3) conspecific neighbors and P((N-3)(1-f)) heterospecific neighbors), and you'd get the same result.

Here is what would convince me that they did find that clustering promotes coexistence (empirically): They need to show that individuals with a high frequency of conspecific neighbors have a higher total number of neighbors. Like, you'd need to have a regression line, where the x-axis is frequency of nearby conspecific neighbors (i.e. # conspecific neighbors / total # of neighbors), the y-axis is total number of neighbors, and each dot is one individual; if that slope was positive, then I'd be convinced. And, it would help a lot too if this impact was stronger for common species than rare ones.

Concern 4: Phylogenetic distance is an inappropriate proxy for beta
One of the critical parameters in this model is beta_fi / beta_ff. When fitting to the empirical distribution, they did not actually measure this parameter. Instead, they used phylogenetic distance as a proxy. This is inappropriate for two reasons. First, this is not a great proxy, e.g. (Godoy et al. 2014). Second, it makes the implicit assumption that beta_fi / beta_ff < 1 for all species, i.e. they are assuming that intraspecific competition is stronger than interspecific competition for all species (even before they get into intraspecific clustering). If you create any model with that assumption, species are likely to coexist.

I think the better assumption would have been to assume that beta_fi / beta_ff = 1 for all species pairs. In this case, you are eliminating all coexistence mechanisms other than intraspecific clustering. Or, alternatively, compare a model where beta_fi / beta_ff = 1 for all species to one with reasonable estimates for beta_fi / beta_ff, and see how much stronger the later is (if they are about the same, it means that intraspecific clustering truly has a big effect; if the later is much larger, it suggests that intraspecific clustering is tiny compared to other mechanisms).

Concern 5: Intraspecific clustering combined with conspecific negative density-dependence tends to weaken the stabilizing mechanism

Their model assumes that species are clustered (the D term), and that they experience conspecific negative density-dependence (the B term). Previous work has shown that these two things will actually counteract each other (Stump and Chesson 2015, Usinowicz 2015). The intuition is fairly simple: If species are clustered, then even rare species will tend to experience CNDD, which reduces the advantage to rarity. In fact, under some circumstances, this can lead to competitive exclusion (Murrell 2010, Miranda et al. 2015, Stump and Comita 2018). I am not sure why the model did not find this. I think that they implicitly assumed that clustering would vanish for rare species. Is there evidence for this? If anything, I think evidence would suggest that this does not happen (Condit et al., 2000).

Concern 6: Their mathematical analysis is flawed
As part of their analysis, the authors combined all of the heterospecific effects, and then treat the average as a constant (around line 369). This is not valid, especially if every species interacted with each heterospecific different (which would not be true for classic density-dependence if beta_fi/beta_ff is equal to phylogenetic distance, and fig. 2b makes it look like this is a really bad assumption WAB, Baihua, DHS, and FS). Instead, we expect the mean of k will change when the frequency of competitors change.

The frequency of competitors will basically always change if species do not interact with all heterospecifics the same. This was shown previously for classic density-dependence in (Stump 2017), and I imagine the same would be true if species clustered with some heterospecifics more than others (e.g. if there was phylogenetic signal in the k_fm). The big problem with this is that species with a lot of phylogenetic neighbors experience more interspecific competition, which can actually cause it to be excluded. There is also a bit of an impact on the stabilizing mechanism: when a species becomes rare, it tends to be replaced by its relatives, which makes bouncing back harder.

Here is how the authors can prove me wrong: They need to make a model that does not treat all heterospecific competitors the same, but instead parameterizes the beta and clustering parameters separately for each species pair. Then, run a simulation, and see if the species coexist. I might be wrong, but I think they will often find that some species are excluded (like, even if they make the inappropriate assumption that beta_fi/beta_ff is proportional to phylogenetic distance).

Minor concern 1: The title is misleading
Species are not identical if they interact with conspecifics differently than heterospecifics. At least I (and I think most ecologists) would interpret “identical” as something like in the neutral model. This model is far from it. What they show here is that species with no fitness differences and a stabilizing mechanism can coexist, which is neither novel nor surprising.

Minor concern 2: The parsimony argument does not seem reasonable
I really disagree with the premise that there is probably a simple universal mechanism that can explain biodiversity, and I can think of few community ecologists who believe it is true (I noticed they don’t have citations for that claim, lines 70-74). And I really disagree with the principal of parsimony argument – you could use the same argument to claim that by parsimony, all of physics should be explained by one force (rather than 4), all matter should be made of one type of quark (instead of 6), and so on. I don’t think there is any legitimate reason to think that there should be one singular stabilizing mechanism that can explain biodiversity.

Citations:

Chesson, P. 2000. General theory of competitive coexistence in spatially-varying environments. Theoretical Population Biology 58:211–237.
Condit, R., P. S. Ashton, P. J. Baker, S. Bunyavejchewin, S. Guantilleke, N. Guantilleke, S. P. Hubbell, R. B. Foster, A. Itoh, J. V Lafrankie, H. S. Lee, E. Losos, N. Manokaran, R. Sukumar, and T. Yamakura. 2000. Spatial Patterns in the Distribution of Tropical Tree Species. Science 288:1414–1418.
Godoy, O., N. J. B. Kraft, and J. M. Levine. 2014. Phylogenetic relatedness and the determinants of competitive outcomes. Ecology Letters 17:836–844.
Hubbell, S. P. 1979. Tree dispersion, abundance, and diversity in a tropical dry forest. Science 203:1299–1309.
Ives, A. R. 1988. Covariance, coexistence and the population dynamics of two competitors using a patchy resource. Journal of Theoretical Biology 133:345–361.
Miranda, A., L. M. Carvalho, and F. Dionisio. 2015. Lower within-community variance of negative density dependence increases forest diversity. PLOS ONE 10:e0127260.
Murrell, D. J. 2010. When does local spatial structure hinder competitive coexistence and reverse competitive hierarchies? Ecology 91:1605–1616.
Stump, S. M. 2017. Multispecies coexistence without diffuse competition; or, why phylogenetic signal and trait clustering weaken coexistence. American Naturalist 190:213–228.
Stump, S. M., and P. Chesson. 2015. Distance-responsive predation is not necessary for the Janzen-Connell hypothesis. Theoretical Population Biology 106:60–70.
Stump, S. M., and L. S. Comita. 2018. Interspecific variation in conspecific negative density dependence can make species less likely to coexist. Ecology Letters 21:1541–1551.
Usinowicz, J. 2015. Limited dispersal drives clustering and reduces coexistence by the storage effect. The American Naturalist 186:634–648.

Reviewer #2 (Remarks to the Author):

A new spatial coexistence theory is developed alongside analyses of data from large observational forest plots. The result is found that the means of spatial aggregation of babies may have important implications for the coexistence of species.

I took reading this paper several times to come around to it. I think there is a significant contribution here, but the framing of the paper sometimes strays from a scientific presentation of that contribution. In particular, it seems to me that the novel finding of this paper is the result that the aggregation of recruits, independent of parents can lead to stable species coexistence despite an assumption that all other aspects of species are equivalent.

I would appreciate if this finding were more thoroughly described in the paper and the findings were tested against the data, or if the findings were used to bring new interpretations to these rich datasets.

I think this is a very exciting new finding, but I found the language tauting the novelty of the model and the shortcomings of previous theoretical work actually distract from it. There is a lot of very good work on spatial theories of species coexistence. And it's not entirely clear to me that the model you presented here could be interpreted as "ecological equivalence." The aggregation of plants does likely require some niche preference of the seed disperser or the plant species, and thus the plants may be ecologically equivalent on the most commonly examined axes, but actually are not equivalent in the dependence of an individuals' recruitment location on other individuals.

I may be splitting hairs. But, I think, if it must be assumed that the dispersal location of the offspring of one tree depends in any way on the dispersal locations of other trees of the same species, this is not ecological equivalence. However, if the aggregation needed for stable coexistence could be achieved by aggregation of the individuals dispersed from single parents, only, and all species have such aggregation, I think it would be justified to call that ecological equivalence.

Other major comments:

The use of the number of individuals for the crowding index deserves explanation. Individuals in these forest dynamics plots range widely in sizes. Further, there are so many more small individuals than large individuals that it is likely that a high crowding index would indicate that the individual is actually just not close to a big individual.

Figure 4 is a compelling theoretical result. But, it's actually unclear that Panel B would match real data better. This deserves some explanation or rather a prediction that takes full advantage of the power of the data in the paper.

Further, the data use is very difficult to follow. I see that model parameters are calculated, but I cannot find evidence for the statement "We show that spatial patterns observed at nine temperate, subtropical, and tropical forest megaplots agree well with predictions of our theory."
Perhaps this paper is trying to do too much in too little space. The theoretical finding about aggregation does not need the data (and it's assumptions violate the data measurement (Bif=Bff, as far as I can tell). But there is an attempt to show that a tweaked version of that model without ecological equivalence accurately captures spatial patterning as well. This is difficult to follow in the manuscript's current state. What is the reader meant to take away from Figure 3? Would this figure be more appropriate to the appendix? A figure that captures the accuracy of the model at predicting the data is missing.

Minor comments:

The meaning of "feasibility" in the abstract is not clear.

Line 63: "pushes the field" This language seems inappropriate for a research article to me. It belongs in a commentary or similar.

Line 68: Typos/grammatical errors in the first sentence.

Line 102: But all the plots are not species rich?

Line 103: "It is a relief... ordered states." Needs explanation.

Figures need explanations of variables. Variable definitions at other key places of the manuscript would also greatly enhance clarity.

Line 177-178 The implications of this for those looking for niches to explain species diversity, for me, hinges on whether this can be deemed ecological equivalence un the definition I suggest. From the detail in the methods I could not yet determine this.

Simulation code would be useful and is now a fairly standard practice to provide.

****

**Although we cannot offer to publish your paper in Nature Ecology & Evolution, the work may be appropriate for another journal in the Nature Research portfolio. If you wish to explore suitable journals and transfer your manuscript to a journal of your choice, please use our manuscript transfer portal. If you transfer to Nature-branded journals or to the Communications journals, you will not have to re-supply manuscript metadata and files. This link can only be used once and remains active until used.
All Nature Research journals are editorially independent, and the decision to consider your manuscript will be taken by their own editorial staff. For more information, please see our manuscript transfer FAQ page.

** For Nature Research general information and news for authors, see http://npg.nature.com/authors.

Author Rebuttal to Initial comments

General comments

The focus of our analysis is development of an analytical theory to quantify the influence of processes occurring at the scale of individual plants (the microscale) and of the resulting emerging spatial patterns (at the mesoscale) for multispecies coexistence and dynamics at the population and community scale (the macroscale). In Table 1 we clarify the different scales of the data analysis and the models used in our manuscript. We hope that this helps to clarify some of the misunderstandings.

Specifically, the individual-based model produces the same type of individual-based data than the ForestGeo data sets, and therefore, the emerging mesoscale patterns can be directly compared (blue arrow). First, we used spatial statistics to derive from the individual-level neighborhood crowding indices measures of the emerging spatial patterns at the mesoscale (red arrows) (lines 347-377). Second, we develop methods to integrate neighborhood crowding indices (operating at the individual plant scale) into an analytical spatial model (operating at the meso- and macroscale) (lines 327-345) (green arrow). The analytical spatial model is then used to analyze feasibility and stability (magenta arrow). The analytical non-spatial model is a point of departure.

Table 1. Scheme to clarify scales of data analysis and the three models

**Scale**	**Individual-based ForestGEO data**	**Individual-based neutral simulation model** (lines 506-547)	**Analytical spatial multispecies model** (eq. 2)	**Analytical non-spatial multispecies model** (eq. 1)
**Description**	Spatial point process theory to quantify spatial patterns at the mesoscale, based on microscale data	Simulates behaviour and interactions of individuals at the microscale, patterns at the meso- and macroscale emerge	Describes multispecies dynamics based on demographic parameters s_f, r_f and $ß_{ff}$ and emerging spatial quantities D_f, B_f, C_f	Describes multispecies dynamics based on demographic parameters s_f, r_f and species-level interaction coefficients $ß_{fi}/ß_{ff}$
**Macroscale**	Abundances $N_{f,t}$	Abundances $N_{f,t}$, coexistence/extinction, ratio D_fB_f/K_f (eq. 4),	Abundances $N_{f,t}$, coexistence/extinction, ratio D_fB_f/K_f (eq. 4), invisibility (eq. M20), eigenvalues	Abundances $N_{f,t}$, coexistence/extinction, invisibility, eigenvalues
**Mesoscale** average properties of individuals	Emerging spatial quantities D_f, B_f, k_{ff} (Fig. 2) diffuse neighbourhood interactions (p. 36)	Emerging spatial quantities D_f, B_f, k_{ff}, and their dynamics (p. 37). The B_f are the emerging species level interaction coefficients	Emerging quantities D_f, B_f, k_{ff}. Parameter s_f, r_f and $ß_{ff}$ are given. The B_f are the emerging species level interaction coefficients	Demographic parameters s_f, r_f and species-level interaction coefficients $ß_{fi}/ß_{ff}$ are given
	Spatial statistics	Spatial statistics		
**Microscale** properties of the individuals	location and species identity of all individuals and distance matrix $ß_{fi}/ß_{ff}$ are given	location and species identity of all individuals, demographic parameters s_f, r_f, $ß_{ff}$ and individual-level interaction coefficients $ß_{fi}/ß_{ff}$ are given.		

Reviewer expertise:

Reviewer #1: modelling species coesxistence in tropical tree communities

Reviewer #2: mechanistic modelling of global and landscape scale plant communities

> **Response:** We want to thank the reviewers for their time and effort to point to critical issues of our study that allowed us to more clearly outline our key advances.

Reviewers Comments:

Reviewer #1 (Remarks to the Author):

Summary

How species coexist is a big question in ecology, and we don't have a simple, universal mechanism. The authors think we should (at least for tree communities, lines 70-74), and they think that the mechanism is intraspecific clustering (which they claim coexistence theory doesn't consider, line 75).

> **Response:** We admit that our wording was unclear. We do of course not claim that only one mechanism could explain species coexistence, but we claim that one fundamental factor (spatial patterns) has been neglected (lines 74-77). Our hope is that including spatial patterns into coexistence theory, when considering together with other mechanism, may allow for a simpler and more universal explanation of coexistence.

The authors develop a fairly simple model of population dynamics, where a species' probability of mortality increases in the presence of others (where "the presence of others" is a combination of clustering and interspecific/intraspecific competition). They derive conditions for coexistence. Then, using data from 9 forest dynamics plots, the authors attempt to quantify some of the important parameters. Their data suggests that the species in 8 of these 9 plots are coexisting (with the exception being BCI). They also construct an individual based model, where they show that intraspecific clustering away from the parent can promote coexistence.

> **Response:** The last statement is not fully correct. We show how competitive interactions at the individual scale (with equal strength of con- and heterospecific interactions) together with a specific way of placing recruits with respect to adults generates specific spatial patterns (stable intraspecific aggregation and interspecific segregation) that promote coexistence.
>
> Our model is purposefully simple, but when eliminating spatial patterns (i.e., $D_f = 1$, $k_{ff} = 1$) it is of similar complexity as the model of annuals used in Godoy et al (2014) that formed the core of much of the contemporary coexistence theory.

Overview

I think it paper should be rejected. Basically, the work is incorrect at times, I feel the authors are overselling their results, and many of the key ideas are not novel. I have made a list of my six biggest concerns (and two smaller concerns) below.

> **Response:** Thank you for this effort. We respond to each concern and we conducted the analyses needed to convince you.

Concern 1: Intraspecific clustering not novel

A number of models have previously shown than intraspecific clustering can promote coexistence (Ives 1988, Chesson 2000). It is covered by coexistence theory, it is a form of fitness-density covariance. I've never heard this idea applied to plants, which is moderately novel, but I don't think it is Nature Ecology & Evolution-worthy novel. It has also been known for a really long time that most species show some degree of intraspecific clustering (Hubbell 1979).

> **Response:** The hypothesis that intraspecific aggregation and interspecific segregation can promote coexistence in plants was e.g., put forward by Murrell et al. (2001: TREE 16: 529-530) and questioned by Chesson & Neuhauser (2002: TREE 17: 210-211) using a model that generated conspecific aggregation based on neighbourhood dispersal. They showed, in agreement with our results (fig. 4A), that intraspecific aggregation and interspecific segregation does not lead under such conditions to coexistence.
>
> However, as outlined by reviewer 2, a key contribution of our study is that aggregation of recruits, independent of parents can lead to stable species coexistence despite an assumption that all other aspects of species are equivalent. This condition has been overlooked in analytical and simulation studies. Note however that a study by Getzin et al. (2014: Proc. R. Soc. B 281: 20140922) showed that recruits at BCI are in most cases spatially independent distributed with respect to large conspecific trees.
>
> The fitness-density covariance is not the coexistence mechanism in our model, the coexistence mechanism rather emerges from a rare species advantage due to segregation from heterospecifics and approximately constant aggregation k_{ff}.
>
> Equation M6 shows that competition is governed in our analytical spatial model for $B_f = 1$ (i.e., individuals compete equally with con- and heterospecifics) basically by the term $k_{ff} N_f + k_{fh} \sum_{i \neq f} N_i$ where $k_{ff} > 1$ (intraspecific aggregation) and $k_{fh} < 1$ (intraspecific segregation). Thus, at the population level (but not at the individual level) a conspecific neighbour exerts stronger competition compared to a heterospecific neighbour. This creates a rare species advantage if aggregation k_{ff} remains approximately constant with respect to abundance. Note that this effect is not an assumption of our model but arises due to the neighbourhood competition and dispersal at the individual scale. However, if aggregation increases with decreasing abundance (i.e., a power law $k_{ff} = 2700/N_f^{0.85}$;

Extended data Figure 4), the rare species advantage is lost and the dynamics becomes unstable. See also concern 3 below.

Concern 2: The model has 2 mechanisms promoting coexistence, and they only acknowledge the second one

In every model of stable coexistence, intraspecific competition has to somehow be stronger than interspecific coexistence. Their model has two terms that can produce this effect: D (segregation/aggregation) and B (relative competitive effect). D is the novel mechanism here, and B is simply a very direct for of interspecific competition being stronger than interspecific competition (i.e. classic conspecific negative density dependence). They do not really focus on B at all, which is somewhat okay, except that it means that coexistence may not be driven by D. And, I think that B is driving some of their empirical results (explained below).

> **Response:** The limited space did not allow us to present more detailed analyses of the *B* term, but equation 4 shows clearly how the B_f, D_f and K_f terms interact in promoting coexistence: they must approach a stochastic equilibrium and the ratio of $D_f B_f / K_f$ must be similar among all species in the community.

Concern 3: Multiple mechanisms can generate intraspecific clustering, and not of them all promote coexistence

For intraspecific clustering to promote coexistence, it is not enough that individuals are clustered. Instead, there needs to be something about the environment that causes individuals of a species to cluster in one place and not the other see (Chesson 2000). If conspecifics are clustered just because offspring tend to be dropped in groups, you will not get coexistence (this is basically why the authors didn't get coexistence when clustering was caused by seeds falling near their parent).

> **Response:** We agree that clustering per se is not enough to promote coexistence. Nevertheless, we point to an important coexistence mechanism that has been overlooked so far. We show that three ingredients are needed for coexistence in our neutral model simulations (Fig. 4B):
>
> - heterospecific segregation,
> - conspecific aggregation and
> - independence of aggregation on abundance.
>
> For the latter we assume that recruits are dropped in groups, but without their parents being the cluster centre. The strong effect of recruitment placement on coexistence suggests that one should investigate this mechanism further. For example, Getzin et al. (2014) found that recruits (here individuals that crossing the 1cm dbh threshold) were at BCI for most species spatially independent distributed with respect to large conspecific trees (dbh > 10cm).

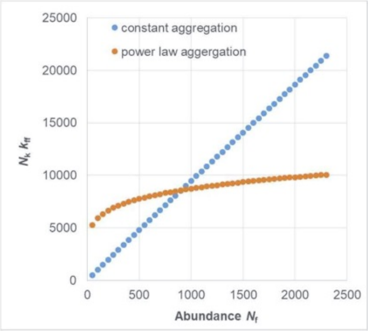

The reason for instability when clustering was caused by recruits falling near their parent (Fig. 4A) is the negative feedback between aggregation and abundance with $k_{ff}(N_f) \approx 2700/N_f^{0.83}$ that removed the rare species advantage. The graph shows the term $k_{ff} N_f$ governing conspecific competition for the case that $k_{ff} \approx 9$ (Fig. 4B in manuscript; orange line) and for the case that aggregation depends on abundance $k_{ff}(N_f) = 2700/N_f^{0.83}$ (Fig. 4A in the manuscript; blue line). The rare species advantage is very much reduced in the latter case.

Basically, if B=1 for all species (i.e. if species are coexisting because of clustering, rather than classical niche partitioning), then what you need to get coexistence is a situation where individuals of a common species are more likely to be near more trees in total; it doesn't work if every tree is near 10 other trees, and clustering just means that many of those are conspecifics.

> **Response:** This is correct and occurs in our model if $B = 1$. The mean number of neighbours of species f is given by $M_f = c\ (k_{ff} N_f + k_{fh} N_h)$ where c is a scaling constant, $k_{ff} > 1$ describes intraspecific aggregation, k_{fh} interspecific segregation, N_h is the number of heterospecifics and $J = N_f + N_h$ is the total number of individuals in the community. It results $M_f = c\ [N_f\ (k_{ff} - k_{fh}) + k_{fh}\ J]$. If aggregation k_{ff} is approximately constant with respect to abundance, individuals of a common species are more likely to be near more trees in total since the second term is the same for rare and abundant species, but the first term is larger (due to the larger value of N_f). As a consequence, rare species have an advantage and show a larger growth rate than abundant species.

I bring this up because I don't think the authors distinguished between the different types of clustering. I'll be honest, I had a lot of trouble understanding how the authors calculated the k variables, so maybe I just misunderstood them (in which case the authors can explain why I am wrong). They showed that, indeed, a given individual is more likely to have a conspecific neighbour than expected by random. However, it is not clear that it is caused by a process that promotes coexistence (rather than, say, clustering caused by dispersal limitation).

> **Response:** Yes, you misunderstood the mechanism of how aggregation/segregation/placement of recruits can generate a stabilizing mechanism. The mechanism is now explained above in response to your comments.

Their data in fig. 1 does not convince me that I am wrong. Specifically, although they fit a Gamma distribution to the number of nearby neighbors, I'd argue that it looks a lot like a Poisson distribution too. This makes me think that the number of seeds at a spot is somewhat random. So,

here is an explanation of what I mean: If we assume that a species has a global frequency of f, and has a mean of N neighbors, then we could model the number of neighbors as a Poisson distribution P(), where it has P(Nf) conspecific neighbors and P(N(1-f)) heterospecific neighbors. Here, you'd probably find some clustering, as some sites would have more neighbors than others. However, when the species becomes rare, it would still have an average of N neighbors, it's just that it would change from conspecifics to heterospecifics (which, if B was the same, wouldn't matter). You could also impose some degree of clustering (like, say, each individual had P((N-3)f + 3) conspecific neighbors and P((N-3)(1-f)) heterospecific neighbors), and you'd get the same result.

> **Response:** Extended data Figure 2e-g show that the number of nearby neighbours are not Poisson distributed, the variance to mean ration of our empirical distributions and that of the simulations are clearly different from one (i.e., the Poisson case).

Here is what would convince me that they did find that clustering promotes coexistence (empirically): They need to show that individuals with a high frequency of conspecific neighbors have a higher total number of neighbors. Like, you'd need to have a regression line, where the x-axis is frequency of nearby conspecific neighbors (i.e. # conspecific neighbors / total # of neighbors), the y-axis is total number of neighbors, and each dot is one individual; **if that slope was positive, then I'd be convinced**. And, it would help a lot too if this impact was stronger for common species than rare ones.

> **Response:** We followed this suggestion, using data from one simulated community with the parameter of Fig. 4B, but we varied the initial species abundances more widely to obtain larger variability in the number of conspecific neighbours.

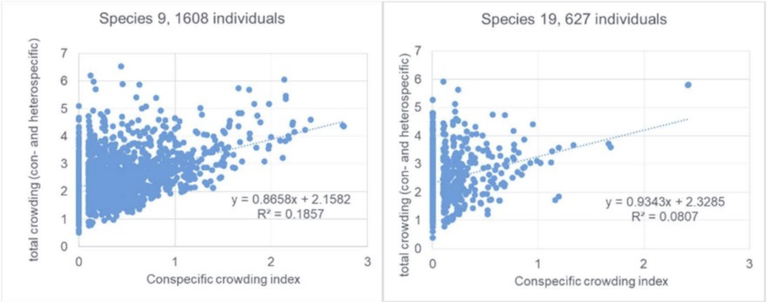

> We plotted at the x-axis the value of the conspecific crowding index of a species *f* and at the y-axis the sum of the corresponding values of the con- and heterospecific crowding indices. Indeed, the slope of the relationship is positive and the relationship is stronger for more abundant species. We show the result for the two species with the lowest and highest abundance.

Concern 4: Phylogenetic distance is an inappropriate proxy for beta

One of the critical parameters in this model is beta_fi / beta_ff. When fitting to the empirical distribution, they did not actually measure this parameter. Instead, they used phylogenetic distance as a proxy. This is inappropriate for two reasons. First, this is not a great proxy, e.g. (Godoy et al. 2014).

> **Response:** Use of phylogenetic distance as a proxy of interactions strength β_{fi}/β_{ff} among individuals is an established approach especially in high diversity communities (see e.g., Uriarte et al. 2010, Fortunel et al. 2016, both cited in the manuscript) to approximate niche differences in the absence of other data. We are happy to apply our method to better proxies of interaction strength.

Second, it makes the implicit assumption that beta_fi/beta_ff < 1 for all species, i.e. they are assuming that intraspecific competition is stronger than interspecific competition for all species (even before they get into intraspecific clustering). If you create any model with that assumption, species are likely to coexist.

> **Response:** Yes, we assume $\beta_{fi}/\beta_{ff} < 1$ to describe competitive interactions among individuals. However, it is not likely that species will coexist only if intraspecific competition is stronger than interspecific competition. Although this is true for two-species competition models, the extrapolation to multispecies models is not always valid (see e.g., Barabás et al. 2018). Stabilizing mechanism are not enough, equalizing mechanism are needed as well. This is also shown in our equation 4. The $B_f D_f / K_f$ must be similar among species to obtain positive abundances, too large variability in the B_f's (that summarize the individual-scale interaction coefficients β_{fi}/β_{ff}) will destabilize.

I think the better assumption would have been to assume that beta_fi / beta_ff = 1 for all species pairs. In this case, you are eliminating all coexistence mechanisms other than intraspecific clustering. Or, alternatively, compare a model where beta_fi / beta_ff = 1 for all species to one with reasonable estimates for beta_fi / beta_ff, and see how much stronger the later is (if they are about the same, it means that intraspecific clustering truly has a big effect; if the later is much larger, it suggests that intraspecific clustering is tiny compared to other mechanisms).

> **Response:** Your first suggestion was already presented in our manuscript: in our neutral individual-based model simulations (Fig. 4) we assumed $\beta_{fi}/\beta_{ff} = 1$ (first suggestion) to investigate the "pure" stabilizing effect of spatial patterns (Fig. 4). Note that we do not claim that only segregation/aggregation can promote coexistence, but that the stabilizing effect of segregation/aggregation is generally overlooked when using non-spatial models.
>
> Result of new analyses to implement your second suggestion are shown in our response to concern 6. The results of the new analyses with reasonable estimates for β_{fi}/β_{ff} must be compared to fig. 4 in our manuscript. Using the BCI phylogenetic data to derive β_{fi}/β_{ff}

seems to destabilize the unstable simulation by creating some very abundant species and keeping most species at lower abundances.

Concern 5: Intraspecific clustering combined with conspecific negative density-dependence tends to weaken the stabilizing mechanism

Their model assumes that species are clustered (the D term), and that they experience conspecific negative density-dependence (the B term). Previous work has shown that these two things will actually counteract each other (Stump and Chesson 2015, Usinowicz 2015). The intuition is fairly simple: If species are clustered, then even rare species will tend to experience CNDD, which reduces the advantage to rarity. In fact, under some circumstances, this can lead to competitive exclusion (Murrell 2010, Miranda et al. 2015, Stump and Comita 2018). I am not sure why the model did not find this. I think that they implicitly assumed that clustering would vanish for rare species. Is there evidence for this? If anything, I think evidence would suggest that this does not happen (Condit et al., 2000).

Response: We agree that conspecific negative density-dependence and clustering can counteract each other.

In our model simulations conspecific negative density-dependence (in our model governed by β_{ff} that entered the K_f term) was the same for all species, this is why the results presented in the manuscript did not show this effect.

However, as suggested by equations 3 and 4 in our manuscript, our model will show this effect if we introduce species differences in conspecific negative density-dependence (i.e., β_{ff}) and species differences in species clustering (i.e., the σ_f parameter). How these two mechanisms interacts in our model is an interesting open question since the issue is complicated because stronger conspecific negative density-dependence reduces intraspecific clustering (i.e., the emerging spatial patterns change as well).

We do not assume that clustering vanishes for rare species (what vanishes with decreasing abundance is the neighbourhood density of conspecifics). Note that the neighbourhood density of conspecifics within distance R is given by $O_{ff}(R) = (N_f/A)\ K_{ff}(R)/(\pi R^2)$ where $K_{ff}(R)$ is Ripley's K, $K_{ff}(R)/(\pi R^2) > 1$ describes clustering (=1 for random patterns) and N_f/A is the number of conspecifics in a plot of area A divided by area.

Concern 6: Their mathematical analysis is flawed

As part of their analysis, the authors combined all of the heterospecific effects, and then treat the average as a constant (around line 369). This is not valid, especially if every species interacted with each heterospecific different (which would not be true for classic density-dependence if beta_fi/beta_ff is equal to phylogenetic distance, and fig. 2b makes it look like this is a really bad assumption WAB, Baihua, DHS, and FS). Instead, we expect the mean of k will change when the frequency of competitors change.

The frequency of competitors will basically always change if species do not interact with all heterospecifics the same. This was shown previously for classic density-dependence in (Stump 2017), and I imagine the same would be true if species clustered with some heterospecifics more than others (e.g. if there was phylogenetic signal in the k_fm).

> **Response:** The constant average is not an assumption but a results of our analysis. The origin of the misunderstanding is that we have basically two sets of interaction coefficients: (i) interaction coefficients β_{fi}/β_{ff} at the microscale that govern the interactions among individuals and (ii) interaction coefficients B_f at the mesoscale that govern the interactions among species in the analytical multispecies models (see table 1). We developed here a method to derive the mesoscale interaction coefficients B_f from the microscale coefficients by using spatial statistics (equations M2 and M5) and we found that the emerging B_f vary indeed very little in time.
>
> The result that the resulting mesoscale interaction coefficients B_f have a simpler structure than the underlying microscale interaction coefficients β_{fi}/β_{ff} is an exciting result.
>
> To be clear, an averaging of the mesoscale interaction coefficients could be flawed for species poor communities (Stump 2017 looked at community with not more than 10 species). But see the study by Wilson et al. (2003: Ecology Letters 6: 944–952) that showed that such a "mean field" averaging provides a good approximation even for mesoscale interaction coefficients. Wilson's et al. argument is that if a community contains many species, then the interaction with any particular species has little influence on the density of another.
>
> In the meantime we also conducted simulations suggested by the reviewer (see below) that confirm that the B_f values are constant, even if the frequency of competitors changes.

The big problem with this is that species with a lot of phylogenetic neighbors experience more interspecific competition, which can actually cause it to be excluded. There is also a bit of an impact on the stabilizing mechanism: when a species becomes rare, it tends to be replaced by its relatives, which makes bouncing back harder.

> **Response:** This was our initial hypothesis in Wang et al. (2016: Ecology 97: 347–360), but spatial analysis showed that this did mostly not happen in species rich forests, but tended to happen somewhat more in species poorer forests. Additional spatial analyses showed that the main effect of stronger interspecific competition is noticed by segregation (the k_{fh} term) but not in the B_f term.

Here is how the authors can prove me wrong: They need to make a model that does not treat all heterospecific competitors the same, but instead parameterizes the beta and clustering parameters separately for each species pair. Then, run a simulation, and see if the species coexist. I might be wrong, but I think they will often find that some species are excluded (like, even if

they make the inappropriate assumption that beta_fi/beta_ff is proportional to phylogenetic distance).

Response: We conducted this analysis. We repeated the analysis of figure 4, but now used the phylogenetic dissimilarity matrix of the BCI plot to parameterize the individual-level competition coefficients β_{fi}/β_{ff} for the 75 species with more than 50 individuals in the data. We then tracked each time step for each species the values of D_f, k_{ff}, k_{fh} and B_f.

Again, clustering recruits near adults produced unstable dynamics with a power law relationship between aggregation and abundance of $k_{ff} \approx 0.85\ N_f^{-0.84}$ and 24 of the 75 species went extinct after 50,000 year (Fig. 1a below). (For comparison, in the simulation of Fig 4 in the manuscript, 28 of the 80 species went extinct after 50,000 years). Note also the wide variation of the species abundances. However, even if most species abundances varied widely in time (the coefficients of variation CV of the different species ranged between 0.05 and 0.98; on average more abundant species showed a lower CV), the population level interaction coefficients B_f varied very little in time (two orders of magnitude less) after a burn-in period, with CV's ranging between 0.001 and 0.008 (Fig. 1c, below).

Clustering away from adults produced stable coexistence over the simulated 50,000 years (Fig. 1b, below) with aggregation not depending on abundance ($k_{ff} \approx 5.6$). Again, the population level interaction coefficients B_f varied very little in time with a coefficient of variation ranging between 0.007 and 0.0014 (Fig. 1d, below). Thus, our result that the population level interaction coefficients B_f are constant in good approximation is confirmed by our new simulations.

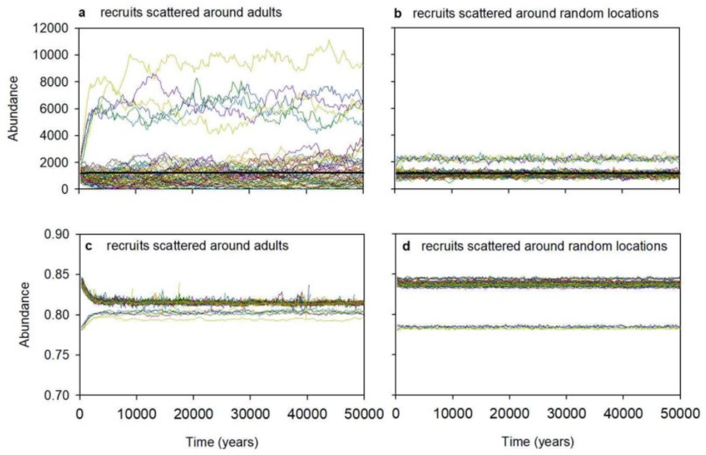

Minor concern 1: The title is misleading

Species are not identical if they interact with conspecifics differently than heterospecifics. At least I (and I think most ecologists) would interpret "identical" as something like in the neutral model. This model is far from it. What they show here is that species with no fitness differences and a stabilizing mechanism can coexist, which is neither novel nor surprising.

> **Response:** This is a misunderstanding. Our model to produce fig 4 is formulated at the individual scale (the microscale) where individuals interact in an identical way to conspecifics and heterospecifics. We dedicated substantial effort to developed novel methods (p 16 - 19) that allow us to upscale the behaviour of individual plants (that created spatial patterns) to the population and community scale (the macroscale) and to extract correction factors accounting for the effect of spatial patterns on community dynamics in an analytical model at the population/community scale.
>
> Of course, the stabilizing effect becomes obvious when looking at the spatial multispecies model as noticed by the reviewer, but we demonstrate here how competition at the individual scale and clustered placement of recruits generate in a neutral model a stabilizing mechanism. This is a novel and surprising result.

Minor concern 2: The parsimony argument does not seem reasonable

I really disagree with the premise that there is probably a simple universal mechanism that can explain biodiversity, and I can think of few community ecologists who believe it is true (I noticed they don't have citations for that claim, lines 70-74). And I really disagree with the principal of parsimony argument – you could use the same argument to claim that by parsimony, all of physics should be explained by one force (rather than 4), all matter should be made of one type of quark (instead of 6), and so on. I don't think there is any legitimate reason to think that there should be one singular stabilizing mechanism that can explain biodiversity.

> **Response:** We admit that our wording was not clear enough and caused confusion. We do of course not claim that only one mechanism could explain species coexistence, but we claim that one fundamental factor (spatial patterns) has been neglected (lines 74-77). Our hope is that including spatial patterns into coexistence theory, when considering together with other mechanism, may allow for a simpler and more universal explanation of coexistence. In both phrases following the confusing parsimony statement we clearly state that other factors are also important and operating; otherwise we would not include the beta_fi's into our model!

Citations:

Chesson, P. 2000. General theory of competitive coexistence in spatially-varying environments. Theoretical Population Biology 58:211–237.

Condit, R., P. S. Ashton, P. J. Baker, S. Bunyavejchewin, S. Guantilleke, N. Guantilleke, S. P. Hubbell, R. B. Foster, A. Itoh, J. V Lafrankie, H. S. Lee, E. Losos, N. Manokaran, R. Sukumar, and T. Yamakura. 2000. Spatial Patterns in the Distribution of Tropical Tree Species. Science 288:1414–1418.

Godoy, O., N. J. B. Kraft, and J. M. Levine. 2014. Phylogenetic relatedness and the determinants of competitive outcomes. Ecology Letters 17:836–844.

Hubbell, S. P. 1979. Tree dispersion, abundance, and diversity in a tropical dry forest. Science 203:1299–1309.

Ives, A. R. 1988. Covariance, coexistence and the population dynamics of two competitors using a patchy resource. Journal of Theoretical Biology 133:345–361.

Miranda, A., L. M. Carvalho, and F. Dionisio. 2015. Lower within-community variance of negative density dependence increases forest diversity. PLOS ONE 10:e0127260.

Murrell, D. J. 2010. When does local spatial structure hinder competitive coexistence and reverse competitive hierarchies? Ecology 91:1605–1616.

Stump, S. M. 2017. Multispecies coexistence without diffuse competition; or, why phylogenetic signal and trait clustering weaken coexistence. American Naturalist 190:213–228.

Stump, S. M., and P. Chesson. 2015. Distance-responsive predation is not necessary for the Janzen-Connell hypothesis. Theoretical Population Biology 106:60–70.

Stump, S. M., and L. S. Comita. 2018. Interspecific variation in conspecific negative density dependence can make species less likely to coexist. Ecology Letters 21:1541–1551.

Usinowicz, J. 2015. Limited dispersal drives clustering and reduces coexistence by the storage effect. The American Naturalist 186:634–648.

Reviewer #2 (Remarks to the Author):

A new spatial coexistence theory is developed alongside analyses of data from large observational forest plots. The result is found that the means of spatial aggregation of babies may have important implications for the coexistence of species.

Response: Thank you!

I took reading this paper several times to come around to it. I think there is a significant contribution here, but the framing of the paper sometimes strays from a scientific presentation of that contribution. In particular, it seems to me that the novel finding of this paper is the result that the aggregation of recruits, independent of parents can lead to stable species coexistence despite an assumption that all other aspects of species are equivalent.

Response: Yes, this is one key finding of our paper. The comments of the reviewers help to present our novel contribution more clearly.

I would appreciate if this finding were more thoroughly described in the paper and the findings were tested against the data, or if the findings were used to bring new interpretations to these rich datasets.

Response: Well, we admit that we tried to do too much in too little space and agree that we should concentrate at this finding and bring new interpretation to these data sets.

I think this is a very exciting new finding, but I found the language tauting the novelty of the model and the shortcomings of previous theoretical work actually distract from it. There is a lot of very good work on spatial theories of species coexistence.

Response: We agree. We will downplay the novelty of the model and the shortcomings of previous theoretical work to better outline our key funding.

And it's not entirely clear to me that the model you presented here could be interpreted as "ecological equivalence." The aggregation of plants does likely require some niche preference of the seed disperser or the plant species, and thus the plants may be ecologically equivalent on the most commonly examined axes, but actually are not equivalent in the dependence of an individuals' recruitment location on other individuals.

I may be splitting hairs. But, I think, if it must be assumed that the dispersal location of the offspring of one tree depends in any way on the dispersal locations of other trees of the same species, this is not ecological equivalence. However, if the aggregation needed for stable coexistence could be achieved by aggregation of the individuals dispersed from single parents,

only, and all species have such aggregation, I think it would be justified to call that ecological equivalence.

> **Response:** This is a good point. What we need for stable coexistence is a mechanisms that prevents aggregation from increasing strongly if the species becomes rare. In our simulation model we accomplish this with a Thomas process: we determine for each species a number of N_c randomly located cluster centres (N_c is a parameter and independent on abundance) and place then the resulting number of recruits per cluster (0, 1, 2, 3 or more) with the Gaussian kernel around these locations. The number of recruits per cluster follows a Poisson distribution with mean: Number recruits/number cluster centres. Yes, in the model we can place the offspring of a single parents into a single clusters to justify the ecological equivalence.
>
> However, given the apparent importance of the way recruits aggregate, a formidable task is to identify mechanism that could produce aggregation independent on parent locations. In the manuscript we suspected that canopy gaps and animal seed dispersal would be candidate processes to generate species aggregation independent of parent locations. Even if such mechanism may differ among species, the important message is that they can stabilize.

Other major comments:

The use of the number of individuals for the crowding index deserves explanation. Individuals in these forest dynamics plots range widely in sizes. Further, there are so many more small individuals than large individuals that it is likely that a high crowding index would indicate that the individual is actually just not close to a big individual.

> **Response:** This is a good point, yes tree size will matter and it is usually included into the crowding indices used in individual-based analysis of tree performance. To not overload our method, we ignored tree size in the first step.
>
> However, in the meantime we developed our model further, included tree growth and can now use tree size in the crowding index. The resulting analytical model works analogously, but instead of having aggregation of individuals we have aggregation of basal area. The stabilizing effect of our simpler model is maintained and even enhanced since growth works like a buffer (aggregation of basal area builds up slower than that of the number of neighbours).

Figure 4 is a compelling theoretical result. But, it's actually unclear that Panel B would match real data better. This deserves some explanation or rather a prediction that takes full advantage of the power of the data in the paper.

> **Response:** This is a good point. BCI has the largest number of censuses (starting with 1982/83, 1985, 1990, etc. every 5 years), but even 40 years are too short to assess this.

The prediction favouring panel B over panel A is that recruits should be independently placed with respect to large conspecific trees (shown in Getzin et al. 2014: Proc. R. Soc. B 281: 20140922) and that there is no strong power law relationship between aggregation and abundance (own new analyses). For the other plots this has to be investigated in forthcoming studies.

Another prediction was proposed by reviewer 1: individuals with a high frequency of conspecific neighbours have a higher total number of neighbours (concern 3).

Additionally, we can check the rare species advantage by plotting $k_{ff}*N_f$ of abundance N_f as in our first graph regarding concern 3.

Further, the data use is very difficult to follow. I see that model parameters are calculated, but I cannot find evidence for the statement “We show that spatial patterns observed at nine temperate, subtropical, and tropical forest megaplots agree well with predictions of our theory.”

Response: Ok, we mean (i) that the intraspecific distribution of the crowding indices (Fig. 1, Extended Data Figure 1) match Gamma distributions predicted by our model, (ii) most species show intraspecific aggregation (Fig. 2a) and interspecific segregation (Fig. 2b), and the dilution coefficients D_f (Fig. 2d) and the interaction coefficients $B_f D_f$ (Fig. 2e) are mostly below a value of 0.5 (one condition for more stable dynamics). We agree that lines 127-130 should make this clear.

Perhaps this paper is trying to do too much in too little space. The theoretical finding about aggregation does not need the data (and it’s assumptions violate the data measurement (Bif=Bff, as far as I can tell). But there is an attempt to show that a tweaked version of that model without ecological equivalence accurately captures spatial patterning as well. This is difficult to follow in the manuscript’s current state. What is the reader meant to take away from Figure 3? Would this figure be more appropriate to the appendix? A figure that captures the accuracy of the model at predicting the data is missing.

Response: This is a good point. We a tried to do too much in too little space. As suggested, it would be better to concentrate in the manuscript on the neutral model, the new coexistence mechanism and its consequences.

Minor comments:

The meaning of “feasibility” in the abstract is not clear.

Response: ok, can be easily fixed.

Line 63: “pushes the field” This language seems inappropriate for a research article to me. It belongs in a commentary or similar.

Response: ok,

Line 68: Typos/grammatical errors in the first sentence.

Response: ok, will be fixed.

Line 102: But all the plots are not species rich?

Response: they have all > 20 species, which is relatively species rich.

Line 103: “It is a relief… ordered states.” Needs explanation.

Response: ok.

Figures need explanations of variables. Variable definitions at other key places of the manuscript would also greatly enhance clarity.

Response: ok.

Line 177-178 The implications of this for those looking for niches to explain species diversity, for me, hinges on whether this can be deemed ecological equivalence un the definition I suggest. From the detail in the methods I could not yet determine this.

Response: we will clarify this important issue.

Simulation code would be useful and is now a fairly standard practice to provide.

Response: ok.

Decision Letter, first revision:

12th June 2020

Dear Dr Wiegand,

Thank you for your letter asking us to reconsider our decision on your Article entitled "Spatial coexistence theory shows that ecologically identical species can stably coexist". After careful consideration we have decided that we would be willing to consider a revised version of your manuscript.

Along with your revised manuscript, you should also re-submit a separate point-by-point response to all of the concerns raised by the referees, in each case describing what changes have been made to the manuscript or, alternatively, if no action has been taken, providing a compelling argument for why that is the case. If we feel that a substantial attempt has been made to address the referees' comments, this response will be sent back to the referees - along with the revised manuscript - so that they can judge whether their concerns have been addressed satisfactorily or otherwise.

I should stress, however, that we would be reluctant to trouble our referees again unless we thought that their comments had been addressed in full.

When revising your paper:

- ensure it complies with our format requirements for Articles as set out in our guide to authors at www.nature.com/natecolevol/authors/index.html

- state in a cover note the length of the text, methods and legends; the number of references and the number of display items.

Please ensure that all correspondence is marked with your Nature Ecology & Evolution reference number in the subject line.

Please use the following link to submit your revised manuscript:

[REDACTED]

I would appreciate it if you could tell me if you think you will be able to submit a revised manuscript, and also the likely timescale.

I look forward to hearing from you soon.

[REDACTED]

Author Rebuttal, first revision:

Reviewers Comments:

Reviewer #1 (Remarks to the Author):

Summary

How species coexist is a big question in ecology, and we don't have a simple, universal mechanism. The authors think we should (at least for tree communities, lines 70-74), and they think that the mechanism is intraspecific clustering (which they claim coexistence theory doesn't consider, line 75).

> **Response:** We removed this confusing statement. Our main point is that spatial patterns (such intraspecific clustering and interspecific segregation) may be a missing factor that can contribute to coexistence in species rich plant communities (lines 64-66).

The authors develop a fairly simple model of population dynamics, where a species' probability of mortality increases in the presence of others (where "the presence of others" is a combination of clustering and interspecific/intraspecific competition). They derive conditions for coexistence. Then, using data from 9 forest dynamics plots, the authors attempt to quantify some of the important parameters. Their data suggests that the species in 8 of these 9 plots are coexisting (with the exception being BCI). They also construct an individual based model, where they show that intraspecific clustering away from the parent can promote coexistence.

> **Response:** The new version of our manuscript clarifies that our approach tries to link different scale: we derive species-level interaction coefficients α_{fi} from individual-level processes (and interactions coefficients β_{fi}), and integrate the resulting α_{fi}'s into a simple analytical macroscale multispecies model to study their consequences for multispecies dynamics and coexistence.
>
> Although our model is purposefully simple, the microscale-interactions and the microscale placement of recruit cause complex dynamics (such as the negative feedback between clustering and abundance) that have been overlooked so far. Consider also that our macroscale model is, when eliminating spatial patterns, of similar complexity as the model of Godoy et al (2014) that formed the core of much of the contemporary coexistence theory.

2

Overview

I think it paper should be rejected. Basically, the work is incorrect at times, I feel the authors are overselling their results, and many of the key ideas are not novel. I have made a list of my six biggest concerns (and two smaller concerns) below.

Response: Thank you for this effort. We respond in the following in detail to each of your concerns, and we conducted the analyses you demand to be convinced.

Concern 1: Intraspecific clustering not novel

A number of models have previously shown than intraspecific clustering can promote coexistence (Ives 1988, Chesson 2000). It is covered by coexistence theory, it is a form of fitness-density covariance. I've never heard this idea applied to plants, which is moderately novel, but I don't think it is Nature Ecology & Evolution-worthy novel.

Response: The fitness-density covariance is not the stabilizing mechanism we describe. This is because in our model mortality is higher in locations were the density of neighbours is higher (i.e., due to crowding), and spatial dynamics does not concentrate a species into areas of high fitness (i.e., low mortality). The novel coexistence mechanism underlying Extended Data Figure 5c is explained in our response to concern 3 and in the manuscript (lines 304-310 and 465-481).

Our theoretical analysis and model simulations show clearly that intraspecific clustering alone does not necessarily promote coexistence. We show that three main ingredients are needed for stable coexistence: (i) the population level interaction coefficients must satisfy $\alpha_{fi}/\alpha_{ff} < 1$ (this is well known), (ii) clustering must not increase too much if abundance decreases (this is novel) and (iii) we present an analytical expression for the multi-species equilibrium (eq. 8) that reveals under which combination of model parameters and emerging patterns species abundances are positive (eq. 9) (this is novel).

As outlined by reviewer 2, condition (ii) is a key contribution of our study that shows that clustering of recruits, independent of parents, can lead to stable species coexistence despite an assumption that all other aspects of species are equivalent. This condition has been overlooked in previous analytical and simulation studies. Note however that a study by Getzin et al. (2014: Proc. R. Soc. B 281: 20140922) showed that recruits at BCI are in most cases spatially independent distributed with respect to large conspecific trees.

In the case of a neutral model where condition (ii) is given, we obtain stable coexistence if species show intraspecific clustering and intraspecific segregation (which yields $\alpha_{fi}/\alpha_{ff} < 1$; see Extended Data Figure 5c,f,i,o). The hypothesis that intraspecific clustering and interspecific segregation can promote coexistence in plants is not new, it was e.g., put forward by Murrell et al. (2001: TREE 16: 529-530). However, it was questioned by Chesson & Neuhauser (2002: TREE 17: 210-211)

based on a model that generated conspecific clustering based on neighbourhood dispersal. They showed, in agreement with our results (Extended Data Figure 5b), that intraspecific clustering and interspecific segregation does not lead under such conditions to coexistence.

It has also been known for a really long time that most species show some degree of intraspecific clustering (Hubbell 1979).

Response: This is true, but why should this compromise the novelty of our results?

Concern 2: The model has 2 mechanisms promoting coexistence, and they only acknowledge the second one

In every model of stable coexistence, intraspecific competition has to somehow be stronger than interspecific coexistence. Their model has two terms that can produce this effect: D (segregation/aggregation) and B (relative competitive effect). D is the novel mechanism here, and B is simply a very direct for of interspecific competition being stronger than interspecific competition (i.e. classic conspecific negative density dependence). They do not really focus on B at all, which is somewhat okay, except that it means that coexistence may not be driven by D. And, I think that B is driving some of their empirical results (explained below).

Response: Yes, the importance of the B_f term was not clear enough outlined. We now consider and acknowledge both terms (i.e., B_f and D_f) equally in their ability to make intraspecific competition stronger than interspecific competition (lines 176-184). We also replaced the product $B_f D_f$ by the ratio α_{fi}/α_{ff} to better outline the population-level balance between the strength of con- and heterospecific competition at the population level.

We also conducted additional simulations of the spatially-explicit simulation model asked for by the reviewer to feature the B_f term by using individual-level interaction coefficients β_{fi} that differed between con- and heterospecifics (Extended Data Figures 7 and 8). We obtained results similar to that of the neutral model, but the equilibrium abundances differed now among species in response to the B_f term, and the B_f term could stabilize or cause low abundance and extinctions.

Concern 3: Multiple mechanisms can generate intraspecific clustering, and not of them all promote coexistence

For intraspecific clustering to promote coexistence, it is not enough that individuals are clustered. Instead, there needs to be something about the environment that causes individuals of a species to cluster in one place and not the other see (Chesson 2000). If conspecifics are clustered just because offspring tend to be dropped in groups, you will not get coexistence

(this is basically why the authors didn't get coexistence when clustering was caused by seeds falling near their parent).

> **Response:** We agree that clustering per se is not enough to promote coexistence. However, as also outlined by reviewer 2, we obtained the novel results that the way of how recruits are placed relative to conspecific adults can decide on stability or instability, and we could precisely reveal the mechanism for the frequently observed instability of spatially-explicit neutral models: dispersal limitation produces a negative power-law relationship between clustering where even rare species will tend to experience CNDD, which reduces the advantage to rarity and leads to exclusion.
>
> We identified an important coexistence mechanism that has been overlooked so far: recruits must be placed in space in a way that intraspecific clustering (which is here precisely defined as the value of k_{ff}) remains in approximation constant (Extended Data Figure Fig. 5C). There are certainly a variety of processes able to accomplish this. Some of them may be related with the environment that causes individuals of a species to cluster in one place and not the other. However, other processes to accomplish this may be unrelated with the environments, such as contagious seed dispersal by animals that visit determinate feeding roosts, latrines, or sleep trees that may cause recruits to grow close together or forest gaps may imprint clumped distributions of recruits of pioneer species.
>
> In any case, the strong effect of recruitment placement on coexistence suggests that one should investigate this mechanism further. For example, Getzin et al. (2014) found that recruits (here individuals that crossing the 1cm dbh threshold) were at BCI for most species placed independently with respect to large conspecific trees (dbh > 10cm).

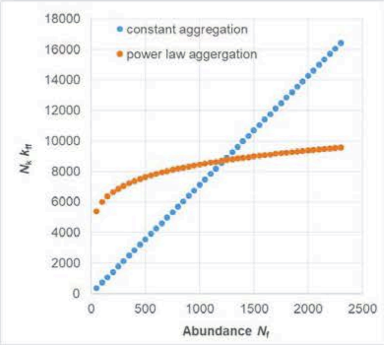

Fig. R1. The reason for instability when clustering was caused by recruits falling near their parent (Extended Data Figure Fig. 5B) is the negative feedback between clustering and abundance with $k_{ff}(N_f) \approx 3000/N_f^{0.85}$ that removed the rare species advantage (see lines 304-310). The blue line in the graph shows the term $k_{ff} N_f$ governing conspecific competition for the case that $k_{ff} \approx 7$ (Extended Data Figure Fig. 5C in manuscript) and for the case that clustering depends on abundance $k_{ff}(N_f) = k_{ff}(N_f) \approx 3000/N_f^{0.85}$ (orange line). The rare species advantage is very much reduced in the latter case.

Basically, if B=1 for all species (i.e. if species are coexisting because of clustering, rather than classical niche partitioning), then what you need to get coexistence is a situation where individuals of a common species are more likely to be near more trees in total; it doesn't work if every tree is near 10 other trees, and clustering just means that many of those are conspecifics.

Response: This is correct and occurs in our model if $B = 1$. Note that in the neutral spatially-explicit model a very particular spatial pattern emerges: intraspecific clustering and interspecific segregation means that an individual is surrounded locally on average by more conspecifics neighbours but fewer heterospecific neighbours that expected by the overall intensity of conspecifics and heterospecifics, respectively (i.e., intensity = abundance divided by area of the plot). If clustering is approximately constant with respect to abundance, an individual of a common species is therefore on average more likely to be near more trees in total (compared to an individual of a rare species) because the number of conspecifics in its neighbourhood is elevated (i.e., clustering). Because of intraspecific clustering and interspecific segregation this can occur without an influence of the environment.

Note also that our focus is on communities with many species, in this case there is no or only a very low correlation between the number of conspecific and heterospecific neighbours (see Extended Data Figure 3a). In communities with few species this will probably not be the case.

More formally, the expected number of neighbours of an individual of species f is given by

$$M_f = c\ (k_{ff}\,N_f + k_{fh}\,N_h)$$

where c is a scaling constant, $k_{ff} > 1$ describes intraspecific clustering, $k_{fh} < 1$ interspecific segregation, and N_h is the number of heterospecifics. With $J = N_f + N_h$ being the total number of individuals in the community, the expected number of neighbours of species f is given

$$M_f = c\ [N_f\ (k_{ff} - k_{fh}) + k_{fh}\,J].$$

Thus, if clustering k_{ff} is approximately constant with respect to abundance, individuals of a common species are more likely to be near more trees in total since the second term is the same for rare and abundant species, but the first term is larger for abundant species (due to the larger value of N_f). As a consequence, rare species have an advantage and show a larger growth rate than abundant species (lines 304-310), see also the invasion criterion (lines 464-481).

I bring this up because I don't think the authors distinguished between the different types of clustering. I'll be honest, I had a lot of trouble understanding how the authors calculated the k variables, so maybe I just misunderstood them (in which case the authors can explain why I am wrong). They showed that, indeed, a given individual is more likely to have a conspecific neighbour than expected by random. However, it is not clear that it is caused by a process that promotes coexistence (rather than, say, clustering caused by dispersal limitation).

Response: Thank you for this comment. The combination of spatial point process theory with population models of plant community dynamics is a novel approach and constitutes already an important results that has to be explained more carefully. We

therefore placed the main elements of the derivation of our theory into the results and discussion section, but present more technical details in the methods.

Additionally, to simplify the calculation of the k variables, we used in the revision simpler crowding indices without distance-weighting. The k variables are now directly related to Ripley's K, the most commonly known summary function of spatial point patterns, that basically determines the expected number of neighbours within distance R of an individual. Interestingly, distance-weighting does not influence our results. We hope that our revision clarifies the mechanism of how clustering / segregation / placement of recruits can generate a stabilizing mechanism.

Their data in fig. 1 does not convince me that I am wrong. Specifically, although they fit a Gamma distribution to the number of nearby neighbors, I'd argue that it looks a lot like a Poisson distribution too. This makes me think that the number of seeds at a spot is somewhat random. So, here is an explanation of what I mean: If we assume that a species has a global frequency of f, and has a mean of N neighbors, then we could model the number of neighbors as a Poisson distribution P(), where it has P(Nf) conspecific neighbors and P(N(1-f)) heterospecific neighbors. Here, you'd probably find some clustering, as some sites would have more neighbors than others. However, when the species becomes rare, it would still have an average of N neighbors, it's just that it would change from conspecifics to heterospecifics (which, if B was the same, wouldn't matter). You could also impose some degree of clustering (like, say, each individual had P((N-3)f + 3) conspecific neighbors and P((N-3)(1-f)) heterospecific neighbors), and you'd get the same result.

Response: Yes, this would happen if the distributions would be Poisson. However, the distributions are not Poison distributions. Extended Data Figure 2d-f show that the variance to mean ration of the distributions are generally different from one (i.e., the Poisson case), they are mostly larger.

Here is what would convince me that they did find that clustering promotes coexistence (empirically): They need to show that individuals with a high frequency of conspecific neighbors have a higher total number of neighbors. Like, you'd need to have a regression line, where the x-axis is frequency of nearby conspecific neighbors (i.e. # conspecific neighbors / total # of neighbors), the y-axis is total number of neighbors, and each dot is one individual; **if that slope was positive, then I'd be convinced**. And, it would help a lot too if this impact was stronger for common species than rare ones.

Response: We followed your suggestion and show also empirically that individuals with more conspecific neighbours tend to have indeed a higher total number of neighbours. Note that competition is in our model driven by the total number of neighbours (not frequency), we therefore plot the number of nearby conspecific neighbours (x-axis) and total number of neighbours (y-axis). We used for this analysis the data from one simulated community with the parameter of Extended Data Figure Fig. 5C (but we varied the initial species abundances more widely to obtain larger

variability in the number of conspecific neighbours). Indeed, individuals with more conspecific neighbours tend to have a higher total number of neighbours, and the effect was slightly stronger for species with lower abundance (the slope is 0.93 for species 20 with 567 individuals and 0.71 for species 50 with 1663 individuals)

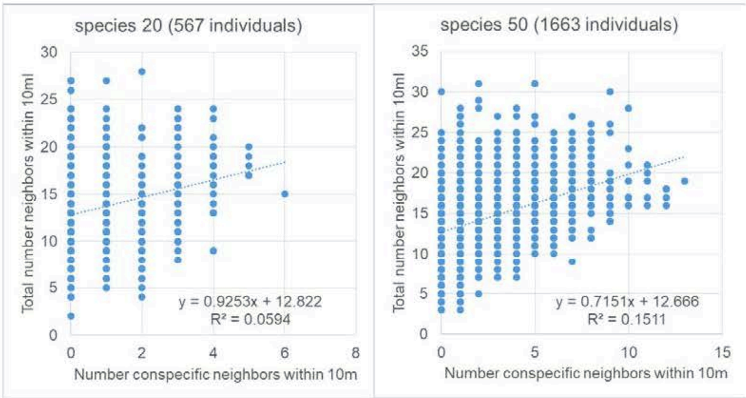

Figure R2. The x-axis is the value of the conspecific crowding index of a species *f* (i.e., number of conspecifics neighbours within 10m) and at the y-axis is the sum of the corresponding values of the con- and heterospecific crowding indices (i.e., total number of neighbours within 10m).

Concern 4: Phylogenetic distance is an inappropriate proxy for beta

One of the critical parameters in this model is beta_fi / beta_ff. When fitting to the empirical distribution, they did not actually measure this parameter. Instead, they used phylogenetic distance as a proxy. This is inappropriate for two reasons. First, this is not a great proxy, e.g. (Godoy et al. 2014).

> **Response:** Use of phylogenetic distance as a proxy of interactions strength β_{fi}/β_{ff} among individuals is an established approach especially in high diversity communities (see e.g., Uriarte et al. 2010, Fortunel et al. 2016, both cited in the manuscript) to approximate niche differences in the absence of other data. We are happy to apply our method in the future to better proxies of interaction strength.

Second, it makes the implicit assumption that beta_fi/beta_ff < 1 for all species, i.e. they are assuming that intraspecific competition is stronger than interspecific competition for all species (even before they get into intraspecific clustering). If you create any model with that assumption, species are likely to coexist.

> **Response:** Yes, we assume in our data analysis $\beta_{fi}/\beta_{ff} < 1$ to describe competitive interactions among individuals. However, it is not likely that species will coexist only if intraspecific competition is stronger than interspecific competition. Although this is

true for two-species competition models, the extrapolation to multispecies models is not always valid (see e.g., Barabás et al. 2018). For example, Extended Data Figures 5n, 7i and 8i show the population-level α_{fi}/α_{ff}'s during the unstable simulations, they are always clearly smaller than one.

I think the better assumption would have been to assume that beta_fi / beta_ff = 1 for all species pairs. In this case, you are eliminating all coexistence mechanisms other than intraspecific clustering. Or, alternatively, compare a model where beta_fi / beta_ff = 1 for all species to one with reasonable estimates for beta_fi / beta_ff, and see how much stronger the later is (if they are about the same, it means that intraspecific clustering truly has a big effect; if the later is much larger, it suggests that intraspecific clustering is tiny compared to other mechanisms).

Response: Your first suggestion was already presented in the manuscript: for the simulations of the neutral model (Extended Data Figures 5) we assumed $\beta_{fi}/\beta_{ff} = 1$ to investigate the "pure" stabilizing effect of spatial patterns. We obtain stable coexistence or unstable dynamics, depending on the way recruits are distributed in space.

We implemented your second suggestion, the results are shown in Extended Data Figures 5 vs. Extended Data Figures 7 and 8. The differences between both cases (i.e., $\beta_{fi}/\beta_{ff} = 1$ vs. $\beta_{fi}/\beta_{ff} \neq 1$) are not too large. As expected, differences among the β_{fi}/β_{ff} lead in cases were recruits are distributed independent on adults to differences in the equilibrium abundances of the species. In case of dispersal limitation differences among the β_{fi}/β_{ff} seems to lead to coexistence of a subset of the species. When we use the Fushan phylogenetic distances to define β_{fi}/β_{ff} we find that all species coexist over the 5000 simulation steps, but the abundances of some species are low and may go extinct later due to demographic stochasticity (Extended Data Figures 7a). In case of our theoretical β_{fi}/β_{ff} based on two traits, 13 species survive the simulation period (Extended Data Figures 8a). Thus, small values of B_f can stabilize otherwise unstable dynamics as predicted by equations 8 and 9. See also the graphs in response to concern 6 that show that equation 6 predicts the equilibrium abundances very well.

Concern 5: Intraspecific clustering combined with conspecific negative density-dependence tends to weaken the stabilizing mechanism

Their model assumes that species are clustered (the D term), and that they experience conspecific negative density-dependence (the B term).

Response: The D term describes intraspecific clustering and interspecific segregation and the B term can be interpreted as mean competition strength of one heterospecific relative to that of one conspecific neighbour. The strength of conspecific negative density dependence is given by the population-level interaction coefficients α_{ff} (eq. 6a), i.e., $\alpha_{ff} = c\gamma_{ff} k_{ff} \beta_{ff}$.

Previous work has shown that these two things will actually counteract each other (Stump and Chesson 2015, Usinowicz 2015). The intuition is fairly simple: If species are clustered, then even rare species will tend to experience CNDD, which reduces the advantage to rarity. In fact, under some circumstances, this can lead to competitive exclusion (Murrell 2010, Miranda et al. 2015, Stump and Comita 2018). I am not sure why the model did not find this.

> **Response:** This is correct and confirmed by our equation 6a ($\alpha_{ff} = c\gamma_{ff} k_{ff} \beta_{ff}$).
>
> Negative conspecific negative density-dependence increases with the parameter β_{ff} (the individual-level interaction strength of conspecifics) and with clustering k_{ff}. For this reason a species can go extinct if clustering is very high or if β_{ff} is very high compared to that of other species (eq. 8).

I think that they implicitly assumed that clustering would vanish for rare species. Is there evidence for this? If anything, I think evidence would suggest that this does not happen (Condit et al., 2000).

> **Response:** Thank you for this comment. No, we do not assume that clustering would vanish for rare species. Clustering must be precisely defined, we used the definition of clustering from point process theory that relates the number of neighbours within distance R to the expected number under a random distribution. There is clustering if the number of neighbours is larger than the expectation, and the ratio between actual and expected number of neighbours is our measure k_{ff} (this is the same index as used in Condit et al. 2000).
>
> With this definition the actual number of conspecific neighbours within distance R is given by $k_{ff}(N_f/A)\,\pi R^2$. (A is the area of the plot). So, an individual of a species with 1000 individuals in a 50 ha plot has on average 6.28 conspecific neighbours within R = 10 m; if abundance is 100, the mean number of conspecific neighbours within distance of 10m is 0.628, and if abundance is 10 the mean number of conspecific neighbours within distance of 10m is 0.0628. Thus, even under constant and strong intraspecific clustering (i.e., k_{ff} = 10) the expected number of conspecific neighbours vanishes if abundance becomes very low.
>
> However, if intraspecific clustering k_{ff} increases with decreasing clustering (e.g., as negative power law as found in our simulation with dispersal limitation), the mean number of conspecific neighbours does not decrease in the same pace as abundance and even rare species will experience CNDD, which reduces the advantage to rarity. This effect is clearly picked up by our model, see also the figure R1 (in response to concern 3) and Extended Data Figure 6a,b.

Concern 6: Their mathematical analysis is flawed

As part of their analysis, the authors combined all of the heterospecific effects, and then treat the average as a constant (around line 369). This is not valid, especially if every species

interacted with each heterospecific different (which would not be true for classic density-dependence if beta_fi/beta_ff is equal to phylogenetic distance, and fig. 2b makes it look like this is a really bad assumption WAB, Baihua, DHS, and FS). Instead, we expect the mean of k will change when the frequency of competitors change.

The frequency of competitors will basically always change if species do not interact with all heterospecifics the same. This was shown previously for classic density-dependence in (Stump 2017), and I imagine the same would be true if species clustered with some heterospecifics more than others (e.g. if there was phylogenetic signal in the k_fm).

Response: The quantity B_f is a key in our analysis. It results from rewriting the population mean of the crowding index $n_{kf\beta}$ that describes heterospecific competition of individual trees (eq. 1a). To avoid confusion we provide now the equation in the manuscript (eq. M2):

$$B_f = \frac{\text{mean}(n_{kf\beta})}{\text{mean}(n_{kfh})} = \frac{\sum_{i \neq f} ck_{fi} N_i(t) \frac{\beta_{fi}}{\beta_{ff}}}{\sum_{i \neq f} ck_{fi} N_i(t)} = \frac{\sum_{i \neq f} ck_{fi} N_i(t) \frac{\beta_{fi}}{\beta_{ff}}}{ck_{fh} \sum_{i \neq f} N_i(t)}$$

The equation shows that B_f is the weighted average of the relative individual-level competition strength of species i (i.e., β_{fi}/β_{ff}), where the weights are the mean number of individuals of species i in the neighbourhoods of individuals of the focal species f [i.e., $c\ k_{fi} N_i(t)$] (lines 423-431). We can expect from the weighting that the index B_f will vary relatively little in time, even if the abundances $N_i(t)$ vary in time and every species interacts with each heterospecific different (i.e., the β_{fi}/β_{ff} differ). Note that the denominator can be rewritten in terms of segregation k_{fh} to all heterospecifics and the total number of heterospecifics $\sum_{i \neq f} N_i(t)$.

We dedicated the section "Test of technical assumptions of the macroscale model" (lines 537-563) and new simulations to test this assumptions. The B_f turned is indeed temporally constant in very good approximation, except some variation due to demographic stochasticity (especially for low abundances) and during the burn-in phase of the simulations. Even for the cases with unstable dynamics, the B_f remained remarkably constant in time (Extended Data Figures 7g, 8g).

Averaging of the population-level interaction coefficients could be indeed flawed for species poor communities (Stump 2017 looked at community with not more than 10 species). But see the study by Wilson et al. (2003: Ecology Letters 6: 944–952) that showed that a "mean field" averaging provides a good approximation even for population-level interaction coefficients. Wilson's et al. argument is that if a community contains many species, then the interaction with any particular species has little influence on the density of another.

The big problem with this is that species with a lot of phylogenetic neighbors experience more interspecific competition, which can actually cause it to be excluded. There is also a bit

of an impact on the stabilizing mechanism: when a species becomes rare, it tends to be replaced by its relatives, which makes bouncing back harder.

Response: Yes, our equation 8 and new model simulations show that species with larger values of B_f compared to that of other species (which means that they compete more intensely with the heterospecifics) are likely to have low abundances. In fact, equation 8 predicts a negative linear relationship between equilibrium abundance and B_f since α_{fi} is proportionally to B_f. This is demonstrated in the left graphs of Figs. R3 and R4 that show the relationship for the last timestep of the simulation shown in Extended Data Figures 8a, b, respectively. Additionally, our analytical model (eq. 8) provides an excellent approximation of the equilibrium abundances, as demonstrated by the right graph of Figs. R3 and R4 that show the corresponding values of the simulated abundances over the abundances predicted with equation 8.

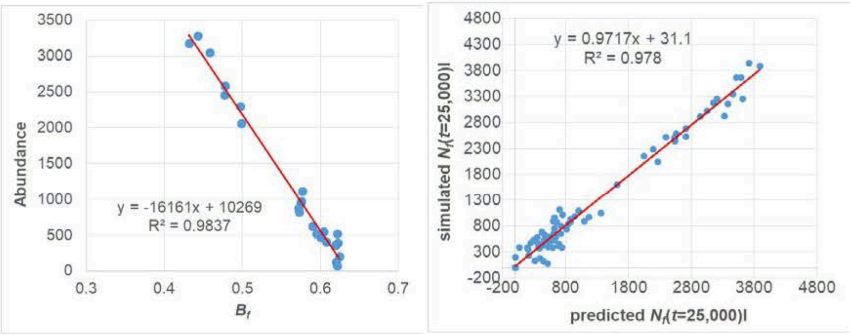

Fig. R3. Additional analyses supplementing Extended Data Figures 8b. left: Observed abundance in model simulations in the last time step versus the values of B_f. for the simulations shown in right: simulated abundances at the last time step versus the abundances predicted by equation 8.

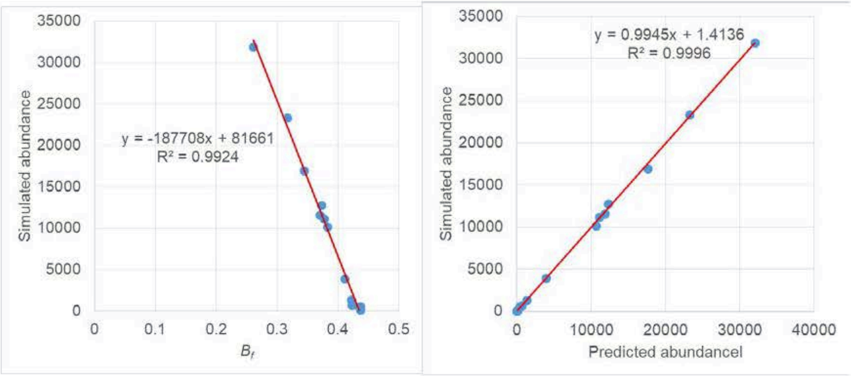

Fig. R4. Additional analyses supplementing Extended Data Figures 8a. left: Observed abundance in model simulations in the last time step versus the values of B_f. for the simulations shown in right: simulated abundances at the last time step versus the abundances predicted by equation 8.

Here is how the authors can prove me wrong: They need to make a model that does not treat all heterospecific competitors the same, but instead parameterizes the beta and clustering parameters separately for each species pair. Then, run a simulation, and see if the species coexist. I might be wrong, but I think they will often find that some species are excluded (like, even if they make the inappropriate assumption that beta_fi/beta_ff is proportional to phylogenetic distance).

> **Response:** This is a good point. We conducted this analysis. We repeated the analysis of Extended Data Figure 5, but now used the phylogenetic dissimilarity matrix of the Fushan plots and a hypothetical distance matrix based on 2 hypothetical traits (similar to Stump 2017) to parameterize the individual-level competition coefficients β_{fi}/β_{ff} for species with more than 50 individuals in the data. We then tracked each time step for each species the values of α_{fh}/α_{ff}, k_{ff}, k_{fh}, B_f, and α_{fh}/α_{ff}.
>
> Again, clustering recruits near adults produced unstable dynamics with a power law relationship between clustering and abundance of $k_{ff} \approx 1971\ N_f^{-0.77}$ and 67 of the 80 species went extinct after 25,000 year (Extended Data Figures 8) and $k_{ff} \approx 1866\ N_f^{-0.79}$ and without extinctions (Extended Data Figures 7). (For comparison, in the simulation of Extended Data Figures 5b, 7 of the 80 species went extinct after 25,000 years). Note also the wide variation of the species abundances. However, even if most species abundances varied widely in time (the coefficients of variation CV of the different species ranged between 0.035 and 0.52 (Extended Data Figures 7a); on average more abundant species showed a lower CV), the population level interaction coefficients B_f varied very little in time (two orders of magnitude less) after a burn-in period, with CV's ranging between 0.002 and 0.011. For Extended Data Figures 8 the CV of abundances varied between 0.064 and 0.95 and the CV of Bf between 0.002 and 0.11.
>
> Clustering away from adults produced stable dynamics with abundances converging into the predicted equilibrium. In some cases the equilibrium was very low and lead to extinction due to demographic stochasticity. Clustering did not depend on abundance and B_f varied very little in time. Thus, our result that the population level interaction coefficients B_f are constant in good approximation is confirmed by our new simulations.

Minor concern 1: The title is misleading

Species are not identical if they interact with conspecifics differently than heterospecifics. At least I (and I think most ecologists) would interpret "identical" as something like in the neutral model. This model is far from it. What they show here is that species with no fitness differences and a stabilizing mechanism can coexist, which is neither novel nor surprising.

> **Response:** We changed the title to better reflect the contents of our study, it is now: "Consequences of spatial patterns for coexistence in species rich plant communities". We also removed the criticized claims on identical species and call it, as proposed by the reviewer, now simply a neutral model.

Well, our simulations model in Extended Data Figure 5 is a neutral model because the model parameters of all species are the same and individual-level interactions are identical among conspecifics and heterospecifics (i.e., all $\beta_{fi}/\beta_{ff} = 1$). Of course, the stabilizing mechanism becomes obvious when looking at the macroscale spatial multispecies model as noticed by the reviewer (and equation 6 in the revision).

However, we showed here analytically (eq. 6) and with the simulation model that the emerging spatial patterns (segregation due to competition, clustering due to clustering of recruits, and the specific placement of recruits relative to conspecific adults) can produce a stabilizing mechanism where conspecific population-level competition is stronger that heterospecific competition (i.e., $\alpha_{fh}/\alpha_{ff} < 1$). Demonstrating this in a neutral model where conspecific and heterospecific individual-level interactions are identical is novel and surprising and in contrast to current knowledge.

Minor concern 2: The parsimony argument does not seem reasonable

I really disagree with the premise that there is probably a simple universal mechanism that can explain biodiversity, and I can think of few community ecologists who believe it is true (I noticed they don't have citations for that claim, lines 70-74). And I really disagree with the principal of parsimony argument – you could use the same argument to claim that by parsimony, all of physics should be explained by one force (rather than 4), all matter should be made of one type of quark (instead of 6), and so on. I don't think there is any legitimate reason to think that there should be one singular stabilizing mechanism that can explain biodiversity.

Response: Thank you for this comment. We admit that our wording was not clear enough and caused confusion. We therefore removed this statement.

Citations:

Chesson, P. 2000. General theory of competitive coexistence in spatially-varying environments. Theoretical Population Biology 58:211–237.

Condit, R., P. S. Ashton, P. J. Baker, S. Bunyavejchewin, S. Guantilleke, N. Guantilleke, S. P. Hubbell, R. B. Foster, A. Itoh, J. V Lafrankie, H. S. Lee, E. Losos, N. Manokaran, R. Sukumar, and T. Yamakura. 2000. Spatial Patterns in the Distribution of Tropical Tree Species. Science 288:1414–1418.

Godoy, O., N. J. B. Kraft, and J. M. Levine. 2014. Phylogenetic relatedness and the determinants of competitive outcomes. Ecology Letters 17:836–844.

Hubbell, S. P. 1979. Tree dispersion, abundance, and diversity in a tropical dry forest. Science 203:1299–1309.

Ives, A. R. 1988. Covariance, coexistence and the population dynamics of two competitors using a patchy resource. Journal of Theoretical Biology 133:345–361.

Miranda, A., L. M. Carvalho, and F. Dionisio. 2015. Lower within-community variance of negative density dependence increases forest diversity. PLOS ONE 10:e0127260.

Murrell, D. J. 2010. When does local spatial structure hinder competitive coexistence and reverse competitive hierarchies? Ecology 91:1605–1616.

Stump, S. M. 2017. Multispecies coexistence without diffuse competition; or, why phylogenetic signal and trait clustering weaken coexistence. American Naturalist 190:213–228.

Stump, S. M., and P. Chesson. 2015. Distance-responsive predation is not necessary for the Janzen-Connell hypothesis. Theoretical Population Biology 106:60–70.

Stump, S. M., and L. S. Comita. 2018. Interspecific variation in conspecific negative density dependence can make species less likely to coexist. Ecology Letters 21:1541–1551.

Usinowicz, J. 2015. Limited dispersal drives clustering and reduces coexistence by the storage effect. The American Naturalist 186:634–648.

Reviewer #2 (Remarks to the Author):

A new spatial coexistence theory is developed alongside analyses of data from large observational forest plots. The result is found that the means of spatial aggregation of babies may have important implications for the coexistence of species.

> **Response:** Thank you!

I took reading this paper several times to come around to it. I think there is a significant contribution here, but the framing of the paper sometimes strays from a scientific presentation of that contribution.

> **Response:** We completely changed the framing of the manuscript to better outline its significant contributions. To accomplish this we focus on our multiscale approach to understanding how pattern-forming processes operating at the level of individuals translate into the emerging spatial patterns and population and community dynamics (lines 67-98). More specifically, this involves deriving the population-level interactions coefficients α_{fi} that appear in population models from individual-level processes and individual-level interactions coefficients β_{fi}. In addition, we shortened the second part of the manuscript.

In particular, it seems to me that the novel finding of this paper is the result that the aggregation of recruits, independent of parents can lead to stable species coexistence despite an assumption that all other aspects of species are equivalent. I would appreciate if this finding were more thoroughly described in the paper and the findings were tested against the data, or if the findings were used to bring new interpretations to these rich datasets.

> **Response:** Yes, this is one key finding of our paper. We now describe this finding more thoroughly in the paper (lines 216-220, 294-303, 304-310). For example, our equilibrium equation 8 already suggests that strong clustering (which can be expected under dispersal limitation where recruitment is located close to conspecific adults) produces low abundances which may lead to extinction.
>
> Given that this is the first paper that describes this approach, we would overload the manuscript if we would test predictions of our study in detail against the rich inventory data. This task is reserved for forthcoming studies. However, we conducted additional data analyses to illustrate our findings and to provide the reader information on the typical values of the quantities that appear in our theory. For example, we show the relationship between individual- and population-level interaction coefficients for the BCI plot (Fig. 3), which show clearly that the corresponding coefficients may differ substantially and that the emerging spatial patterns have a strong potential to shift the population-level balance between con- and heterospecific competition (lines 185-193). Finally we use the data now to find out if predictions of our theory are compatible with the observations (Fig. 4, lines 253-266).

I think this is a very exciting new finding, but I found the language tauting the novelty of the model and the shortcomings of previous theoretical work actually distract from it. There is a lot of very good work on spatial theories of species coexistence.

> **Response:** We agree. We downplayed the novelty of the model and tried to acknowledge more previous theoretical work and to better outline our key funding.

And it's not entirely clear to me that the model you presented here could be interpreted as "ecological equivalence." The aggregation of plants does likely require some niche preference of the seed disperser or the plant species, and thus the plants may be ecologically equivalent on the most commonly examined axes, but actually are not equivalent in the dependence of an individuals' recruitment location on other individuals.

I may be splitting hairs. But, I think, if it must be assumed that the dispersal location of the offspring of one tree depends in any way on the dispersal locations of other trees of the same species, this is not ecological equivalence. However, if the aggregation needed for stable coexistence could be achieved by aggregation of the individuals dispersed from single parents, only, and all species have such aggregation, I think it would be justified to call that ecological equivalence.

> **Response:** This is a good point. Reviewer 1 also discussed the ecological equivalence. In the revision we added a section "Consequences of spatial patterns for coexistence in neutral models" where we now use the term "neutral" since all species in this model have the same parameters and all individual-level competition interaction coefficients are identical.
>
> What we need for stable coexistence is a mechanisms that prevents clustering (measured by Condit et al. 2000's omega which is identical to our k_{ff} of the revision) from increasing if the species becomes rare. In our simulation model we accomplish this with a type of Thomas process: we determine for each species the location of a number of N_c randomly located cluster centres (N_c is a parameter and independent on abundance), and then distribute the recruits with the Gaussian kernel around these locations, with the cluster centre being randomly selected for each recruit. This is now better explained in the methods (lines 500-517 and 526-536). Equally, we can generate the clustering needed for stable coexistence in the simulation model by clustering of the individuals dispersed from single parents.
>
> In real forests there are certainly a variety of processes able to accomplish clustering of recruits independent on conspecific adults. Some of them may be related with the environment that causes individuals of a species to cluster in one place and not the other. However, other processes to accomplish this may be unrelated with the environments, such as contagious seed dispersal by animals that visit determinate feeding roosts, latrines, or sleep trees that may cause recruits to grow close together.

Given the apparent importance of the way recruits aggregate, a formidable task is to identify mechanism that could produce clustering independent on parent locations. In the manuscript we suspected that e.g., canopy gaps and animal seed dispersal would be candidate processes to generate species clustering independent of parent locations. Even if such mechanism may differ among species, the important message is that they can stabilize.

Other major comments:

The use of the number of individuals for the crowding index deserves explanation. Individuals in these forest dynamics plots range widely in sizes. Further, there are so many more small individuals than large individuals that it is likely that a high crowding index would indicate that the individual is actually just not close to a big individual.

Response: This is a good point. Yes tree size will matter and it is usually included into the crowding indices used in individual-based analysis of tree performance. To not overload our methods, we ignored tree size in the first step and to further simplify we also removed the distance dependency (which had no qualitative influence on the results).

However, in the meantime we developed our model further and can now use tree size in the crowding index. The resulting analytical model works analogously, but instead of having clustering of individuals we have clustering of basal area. The stabilizing effect of our simpler model is maintained and even enhanced (clustering of basal area builds up slower than that of the number of neighbours).

Figure 4 is a compelling theoretical result. But, it's actually unclear that Panel B would match real data better. This deserves some explanation or rather a prediction that takes full advantage of the power of the data in the paper.

Response: This is a good point. BCI has the largest number of censuses (starting with 1982/83, 1985, 1990, etc. every 5 years), but even 40 years are too short to assess this.

The prediction favouring panel B over panel A is that recruits should be independently placed with respect to large conspecific trees (shown in Getzin et al. 2014: Proc. R. Soc. B 281: 20140922) and that there is no strong power law relationship between clustering and abundance (own new analyses). For the other plots this has to be investigated in forthcoming studies.

Another prediction was proposed by reviewer 1: individuals with a high frequency of conspecific neighbours should have a higher total number of neighbours (concern 3). Additionally, we can check the rare species advantage by plotting $k_{ff}*N_f$ over abundance N_f as in our first graph regarding concern 3. Given that our manuscript is already long, we must reserve such more detailed test for forthcoming studies.

Further, the data use is very difficult to follow. I see that model parameters are calculated, but I cannot find evidence for the statement "We show that spatial patterns observed at nine temperate, subtropical, and tropical forest megaplots agree well with predictions of our theory."

Response: Ok, we mean (i) that the intraspecific distribution of the crowding indices (Fig. 1, Extended Data Figure 1) match Gamma distributions predicted by our model, (ii) most species show intraspecific clustering (Fig. 2a) and interspecific segregation (Fig. 2b), and α_{fh}/α_{ff} (Fig. 3b) are mostly below a value of 0.5 (one condition for more stable dynamics).

We now better interpret the results of the analysis of the empirical data. We have basically 3 groups of such analyses:

1) we extract information from the data to determine the functional form of our model equations. We show that Gamma distributions fit the empirical distributions of the crowding indices (lines 119-126), the data show that the crowding indices n_{kff} (=x) and $n_{kfß}$ (=y) of individual trees are only weakly, correlated (this justifies use of two independent Gamma distributions in eq. 2, the data show that competition to heterospecifics can be simplified (which is one justification for use of the summary function B_f).

2) we use the data analysis to illustrate the typical values of the quantities appearing in our theory (lines 176-184) and to show the difference between the imposed individual-level and the emerging population-level interaction coefficients (lines 185-193).

3) we use data analysis to assess if our theory produces predictions that are compatible with the observations (lines 253-266).

Perhaps this paper is trying to do too much in too little space. The theoretical finding about aggregation does not need the data (and it's assumptions violate the data measurement (Bif=Bff, as far as I can tell). But there is an attempt to show that a tweaked version of that model without ecological equivalence accurately captures spatial patterning as well. This is difficult to follow in the manuscript. What is the reader meant to take away from Figure 3? Would this figure be more appropriate to the appendix? A figure that captures the accuracy of the model at predicting the data is missing.

Response: Thank you for this comment. We removed figure 3.

Because this is the first manuscript presenting our approach of combining spatial point process theory with analytical multispecies models we decided to put the focus of the manuscript more at the theoretical aspects, and to leave more detailed comparisons of the model to the rich data to forthcoming studies.

Minor comments:

The meaning of “feasibility” in the abstract is not clear.

Response: The abstract was completely rewritten.

Line 63: “pushes the field” This language seems inappropriate for a research article to me. It belongs in a commentary or similar.

Response: we removes such inappropriate language throughout the manuscript.

Line 68: Typos/grammatical errors in the first sentence.

Response: ok, fixed.

Line 102: But all the plots are not species rich?

Response: they have all > 20 species, which is relatively species rich.

Line 103: “It is a relief… ordered states.” Needs explanation.

Response: This phrase was removed.

Figures need explanations of variables. Variable definitions at other key places of the manuscript would also greatly enhance clarity.

Response: done

Line 177-178 The implications of this for those looking for niches to explain species diversity, for me, hinges on whether this can be deemed ecological equivalence un the definition I suggest. From the detail in the methods I could not yet determine this.

Response: We expanded the description of how clustering of recruits was created in the simulation model (lines 500-517 and 526-536). For the interested reader we provide the source code as supplementary information.

Simulation code would be useful and is now a fairly standard practice to provide.

Response: ok. We added a note that the simulation code is available as supplementary information (line 598).

Decision Letter, second revision:

29th September 2020

*Please ensure you delete the link to your author homepage in this e-mail if you wish to forward it to your co-authors.

Dear Dr Wiegand,

Your manuscript entitled "Consequences of spatial patterns for coexistence in species rich plant communities" has now been seen by two reviewers, whose comments are attached. The reviewers have raised a number of concerns which will need to be addressed before we can offer publication in Nature Ecology & Evolution. We will therefore need to see your responses to the criticisms raised and to some editorial concerns, along with a revised manuscript, before we can reach a final decision regarding publication.

While reviewer 1 from the previous round was able to review again, unfortunately reviewer 2 was not, and so we have brought in additional expertise from a third reviewer. We asked reviewer 1 to comment on reviewer 2's report, and I have pasted these comments below.

We therefore invite you to revise your manuscript taking into account all reviewer and editor comments. Please highlight all changes in the manuscript text file [OPTIONAL: in Microsoft Word format].

We are committed to providing a fair and constructive peer-review process. Do not hesitate to contact us if there are specific requests from the reviewers that you believe are technically impossible or unlikely to yield a meaningful outcome.

When revising your manuscript:

* Include a “Response to reviewers” document detailing, point-by-point, how you addressed each reviewer comment. If no action was taken to address a point, you must provide a compelling argument. This response will be sent back to the reviewers along with the revised manuscript.

* If you have not done so already please begin to revise your manuscript so that it conforms to our Article format instructions at http://www.nature.com/natecolevol/info/final-submission. Refer also to any guidelines provided in this letter.

* Include a revised version of any required reporting checklist. It will be available to referees (and, potentially, statisticians) to aid in their evaluation if the manuscript goes back for peer review. A revised checklist is essential for re-review of the paper.

Please use the link below to submit your revised manuscript and related files:

[REDACTED]

Note: This URL links to your confidential home page and associated information about manuscripts you may have submitted, or that you are reviewing for us. If you wish to forward this email to co-authors, please delete the link to your homepage.

We hope to receive your revised manuscript within four to eight weeks. If you cannot send it within this time, please let us know. We will be happy to consider your revision so long as nothing similar has been accepted for publication at Nature Ecology & Evolution or published elsewhere.

Nature Ecology & Evolution is committed to improving transparency in authorship. As part of our efforts in this direction, we are now requesting that all authors identified as 'corresponding author' on published papers create and link their Open Researcher and Contributor Identifier (ORCID) with their account on the Manuscript Tracking System (MTS), prior to acceptance. ORCID helps the scientific community achieve unambiguous attribution of all scholarly contributions. You can create and link your ORCID from the home page of the MTS by clicking on 'Modify my Springer Nature account'. For more information please visit please visit www.springernature.com/orcid.

Please do not hesitate to contact me if you have any questions or would like to discuss these revisions further.

We look forward to seeing the revised manuscript and thank you for the opportunity to review your work.

[REDACTED]

Reviewer expertise:

Reviewer #1: as before

Reviewer #2: unable to re-review

Reviewer #3: Eco-evolutionary dynamics

Reviewers' comments:

Reviewer #1 (Remarks to the Author):

I was reviewer #1 last time. The authors have significantly improved this paper. I'm really impressed about the effort that they took in fixing this. I particularly like how the wrote things in terms of \alpha and \beta, rather than B and D. I was concerned last time that in their models, that individual-level competitive effects were driving their results, and Fig. 3 has convinced me otherwise. I think that makes their results much clearer.

I do still have one really serious concern about their work (my concern #3 from last time). I think that this could be a critical flaw, as it is basically the empirical evidence for their hypothesized mechanism. I suggested what they could do to change my mind, and they did something that was similar, but I

would say not quite good enough. So, I think if they can fix it, then I'd be willing to accept this. But, if this flaw cannot be fixed, then I think this should be rejected.

Re: Comment 3, empirical evidence for clustering.

(Here I'm going to focus on the case where \beta_jj=\beta_jk for all species j and k, as this would eliminate any mechanism other than the novel one in this paper).

Their basic model relies on the assumption that as a species becomes rarer, they encounter conspecifics less, and still encounter roughly the same number of heterospecifics. This mechanism does not work if the number of heterospecifics increases (unless Janzen-Connell effects are important). I guess I'm still not convinced from their data that this is occurring.

Last time I said they could prove me wrong if they showed that there was a positive relationship between the number of nearby trees and the frequency of conspecifics. In figure R2, they showed the total number of conspecifics as the independent variable, rather than frequency. There is a really important distinction here. Say that X is the number of conspecifics, and Y is the number of heterospecifics, and let's assume they are independent. Thus, if you calculate the regression line between conspecifics (X) and total individuals (X+Y), it will be:
cov(X,X+Y)/var(X)
=[cov(X,X)+cov(X,Y)]/var(X)
=var(X)/var(X)
=1.
I don't think this happens if you correlate frequency (i.e. X/(X+Y)) with total number of individuals (X+Y) (at least I ran a few simulations, and it didn't seem to). I'm sure that if there was no effect, one would still get some positive correlations for individual species just by chance, so I wouldn't find one or two graphs like R2 to be convincing. However, if there was a general trend across most species, then I'd be convinced.

The variance to mean ratios were somewhat convincing (while they said the values are "mostly" above 1; however, it looks like 1 is in the middle 50% in many plots, and it is within the middle 90% in most plots). This is enough that I withdraw that comment. However, this still does not prove to me that when a species becomes rare, it will have fewer individuals to compete with.

Like I said before, if rarer species don't start experiencing less competition due to decreased crowding, the mechanism will not work. This seems like a really weird mechanism, one I wouldn't necessarily expect to work like they say it does. If they can show convincing evidence that this mechanism is operating, then I think that this is kind of a big deal, one deserving of a place in Nature E&E. But, if they can't, then I think what they have is basically a modeling paper, and one that is based on assumptions that seem dubious.

Re: Comment 1, the results are not novel.

I do think that what is occurring is a fitness density covariance. Intraspecific competition is the same as interspecific competition (at least in the neutral model). This means it would not be a variation-independent mechanism. The environment is homogenous, suggesting that a spatial storage effect is

not occurring, and they are basically identical, suggesting that a spatial relative nonlinearity is not occurring. Instead, certain factors cause species to become crowded (i.e. have high density), which lowers survival (i.e. have low fitness). If you randomly rearranged all individuals, the effect would go away. Thus, this is a fitness-density covariance.

That said, I see their point on the rest of the response on this one, and this isn't a deal-breaker of a concern.

Re: Comment 6, math problem

The authors assumed that B_f is a constant, when it actually depends on the densities of every species in the community. They argue that in a diverse community, any one species will have little impact on B_f. I don't think that means it can be ignored. The impact of one species on B_f will be roughly inversely proportional to the number of species in the community. However, any stabilizing mechanism will also be inversely proportional to the number of species in the community, so those little impacts can matter.

I agree that in a stable community, B_f will remain largely stable. It is during an invasion that I worry. For example, imagine a 50 species community, where species A and B both have the same disperser (thus allowing those two to be dispersed to unusual microhabitats). If A became rare and B did not change in frequency, it should be easier for A to bounce back (as there is less competition in the microhabitat). However, if A becomes rare and B becomes twice as frequent, then A will have a much harder time bouncing back.

I also don't usually sign my reviews, but since I'm going to talk about my own work, this is Simon Stump. I reran a slightly modified version of the code for Stump (2018, AmNat), this time using communities of between 19 and 28 species (mean 24, using 6 simulations, starting with a species pool of 50). The average invader growth rates were reduced by between 30% and 75% when species had species-specific interaction coefficients vs. the diffuse case (mean 50%). So, I don't think this was purely because 10 species was too species poor. I'm not sure how many you need to consider a community "diverse," and if 24 is not enough, I can try to run more, but I suspect the answer will be the same.

All of this said, their simulations are semi-convincing. If they can fix the issues with comment #3, then I don't my concerns on comment #6 this should prevent this paper from being published. I would only ask that they acknowledge this as a potential problem (and, I understand if they do not want to cite my work, I will not hold it against them if they do not).

Reviewer #1's comments on reviewer #2's initial report:

"on ecological equivalence"

I agree with the reviewer that the species were not "equivalent," and I'd also say that they are not "neutral." Neutral would imply no species differences (and hence no stabilizing mechanisms or fitness differences). But, these species are different, as their seeds disperse into different locations; I agree that it could be due to animal dispersers, but if it is, then there has to be some difference between the

seeds that causes the animals to disperse them into different areas (again, see my commentary about the model in Chesson 2000 TPB, if offspring are dropped in clusters, this won't allow coexistence unless the clusters are dropped in different areas). Really these communities are full of species that are identical other than seed dispersal. I'd worry that calling them "neutral" is misleading for the same reason as before, I'd suggest that the authors try hard to come up with a better term. "Nearly neutral" might work, as they are neutral except for this difference in dispersal (or maybe "otherwise neutral"?). Like, they need to make it clear that the species are different, it's just that inter- and intraspecific competition are the same. I'd also suggest removing "(including neutral communities)" from line 52, and not replacing it with the new term - unless the authors are willing to explicitly describe the term, then the readers won't know what they mean, and it will come across as false advertising.

"the use of the number of individuals for crowding index needs explanation"

If basal area works better, than I'm not sure whey they didn't use it here. At the very least, I'd like them to at least mention that a model using basal area gave similar results.

Reviewer #3 (Remarks to the Author):

This study examined how local competition and spatial distribution affect coexistence of tree species by using spatial point pattern analysis and coexistence theory. The authors combined spatial data of 9 forest plots, spatial statistics, and dynamic models to show that spatial clustering can promote species coexistence. Because of the importance of the topic, this study is of interest to people in this research area. However, I feel the manuscript has some weaknesses to be addressed.

Major comments:
1. What is the novelty of this study? The authors wrote that the "mean-field approximation" is not enough to understand coexistence of diverse tree species (L75), but previous studies already showed conspecific clustering promotes species coexistence by using pair approximations and numerical simulations (e.g., "According to theoretical models, conspecific spatial clustering is recognized as a critical mechanism for reducing competitive exclusion and promoting diversity." in Seidler & Plotkin 2006 PLoS Biol). I think the authors should emphasize the strength of this study (the combination of spatial data of trees, spatial statistics, and dynamical theory) more carefully in Abstract and Introduction.

2. The connections to previous studies are not clear. The authors wrote about "a stabilizing mechanism" (L51-52), but is this the term used in Chesson's coexistence theory (i.e., stabilizing vs. equalizing mechanisms)? I am wondering whether the mechanism of coexistence is "growth-density covariance" of Chesson's coexistence theory. If not, what is the difference? Is it spatial relative nonlinearity or the spatial storage effect? The authors often cited papers on food web theory (e.g., May 1972), but I think it (with several trophic levels) is different from coexistence theory (in a single trophic level).

3. Using empirical data to understand stable coexistence of species-rich communities is an interesting and important topic. For example, Ushio et al. (2018) Nature used time-series data of fish species for estimating interaction strength (and network structure) as well as stability of a Jacobian matrix. Is it possible for the authors to use spatial data for estimating network structures as well as stability of the whole community? It would be great if the authors can add discussion.

4. It will be great if the authors can upload programing codes for statistical analyses and model simulations so that readers can replicate the study.

Minor comments:
L44-46: Satisfying for whom? There are many mechanisms that promote coexistence (e.g., self-regulation, higher-order interactions, temporal & spatial storage effect & relative nonlinerity, growth-density covariance, diversity of interaction types, etc.), so it may be better to avoid the subjective expression.
L46-47: There are already many studies that considered spatial dynamics with local competition. It may be better to emphasize the methodological novelty of this study here.
L48: Timescales of what? I thought "multiscale" means multiple biological scales (individual-level vs. population-level) or multiple spatial scales based on the sentence in L46-47, but it seems that the authors are talking about multiple timescales.
L50-52: I think previous studies showed the similar conclusion. It would be great if the authors can provide a better explanation for readers to easily understand the novelty of this study.
L51-52: "Stabilizing mechanism" for "neutral communities" sounds strange. Do the "neutral communities" mean communities with no niche difference?
L60: Is Darwin (1859) appropriate here? I think its main focus is the origin of species diversity. Hutchinson (1959, 1961) may be more suitable for the maintenance of species diversity.
L62: I think references for food web dynamics (11, 12, 13, 15: May 1972, Allesina & Tang 2012, Stone 2018, Servan et al. 2018) may be irrelevant here. Those studies considered trophic interactions whereas the authors' focus here is coexistence of competitors, right?
L65-66: It may be better to point out here that there are many mechanisms proposed to explain the observed diversity. And please add the explanation why the authors think they are not satisfying.
L70: What is "the dynamic theory of communities"?
L72: Again, I think theory of food web dynamics proposed by May (1972) is not a part of modern coexistence theory.
L75: There are theoretical studies that considered pair-approximations for community dynamics (e.g., Klausmeier & Tilman 2002). Is the pair-approximation not enough?
L94-98: This is a nice summary of this study. It will be nice to revise the abstract as like this part.
L102: Where is reference 27?
L107, legend of Fig. 1, L158-159: It is confusing that the authors sometimes use n_{kfi} and sometimes n_{kfh}. In addition, the authors wrote the definition of h in L158-159. Please be consistent and provide the definition in L107.
L116: "Independent distributions p_x and p_y" sound confusing. Please add more explanations why the authors introduce them here.
L121-122: Please add justifications why the authors can use phylogenetic similarity for the relative competition strength. And how did the authors estimate beta_{ff}?
L125-126: Why did the authors mention that variance-to-mean ratios are not one here? Is it important?
Fig. 1b, c: Why did the authors use circles and arrows with different sizes and widths, respectively? It seems not informative, but confusing.
Legend of Fig. 1: competitive effect beta_{fi}/beta_{ff} → competitive effect beta_{fh}/beta_{ff}
Because the authors used n_{kfh} instead of n_{kfi} here.
Solid lines show → Blue lines show
Because both red and blue lines are solid in Fig. 1d-f.

Why did the authors choose dbh > 10cm? What will happen when we choose different values (e.g., dbh = 1cm, 5cm, or 20cm)?
L131-133: Does this mean beta_{fi} are the same for all heterospecific species and the phylogenetic similarity (L121-122) is not necessary anymore?
L133: Why important?
L142: I was confused: is this contradicting L125-126?
L163-166: Why did the authors assume that heterospecific competition is always weaker than conspecific competition? It is possible for heterospecific competition to be stronger than conspecific competition, it may cause priority effects of community assembly (e.g., Fukami 2015, Ke & Letten 2018).
Legend of Fig. 2: Why did the authors choose R = 10m? What will happen when we choose different values (e.g., R = 1m, 5m, or 20m)?
And what will happen when the authors include rare species with less than 50 individuals?
L195-200: This explanation about Eq. 6b may be moved to L174.
L205: N_i(t)] → N_i(t))]
L206-208: Is it possible to assume that the number of recruits depends on neighborhood competition. What will happen in this case?
L216: Why interesting?
L232-233: Is this because there are no alternative stable states and Allee effects (e.g., Barabas et al. 2018, Schreiber et al. 2019)?
L254: Why interesting?
L295-296: What is the negative feedback between clustering and abundance? Increasing abundance promotes clustering, and clustering reduces the growth rate? If so, why does this cause instability?
L298-299: This seems quite important. What about explaining this in Abstract and Introduction?
L318-325: I think spatial conspecific clustering itself is not a novel mechanism for species coexistence and it will be great if the authors can revise this paragraph by acknowledging previous studies more carefully.
L321-322: Chesson's framework states that species should be different enough for niche differences but similar enough for fitness differences, so this statement seems confusing.
L332: Timescales of what?
L341-345: Please check previous studies carefully: is it really overlooked so far?
L356-357: Where is the result of this statement?
L414: N_i(t)] → N_i(t))]
L620-621, 636-637: References 9 and 17 are the same (Levine et al. 2017).

*******************END*******************

Author Rebuttal, second revision:

Reviewer #1 (Remarks to the Author):

I was reviewer #1 last time. The authors have significantly improved this paper. I'm really impressed about the effort that they took in fixing this. I particularly like how the wrote things in terms of \alpha

and \beta, rather than B and D. I was concerned last time that in their models, that individual-level competitive effects were driving their results, and Fig. 3 has convinced me otherwise. I think that makes their results much clearer.

> **Response:** We are happy that the reviewer found our manuscript significantly improved! We want to thank him again for his time and effort to provide constructive criticisms that helped us to substantially improve our study.

I do still have one really serious concern about their work (my concern #3 from last time). I think that this could be a critical flaw, as it is basically the empirical evidence for their hypothesized mechanism. I suggested what they could do to change my mind, and they did something that was similar, but I would say not quite good enough. So, I think if they can fix it, then I'd be willing to accept this. But, if this flaw cannot be fixed, then I think this should be rejected.

> **Response:** We finally resolved concern #3. The reviewer is right in that the general spatial coexistence mechanism of our model is a specific form of fitness-density covariance. Below we specify the spatial coexistence mechanism of our model in terms of scale transition theory (e.g., Chesson 2012) and we show empirically that coexistence arises in our model for "a situation where individuals of a common species are more likely to be near more trees in total" (see Fig. R1a, b below), as demanded by the reviewer in his first review.
>
> We specify the coexistence mechanism of our model (lines 284-291), we provide the methods for this in a new methods section "Fitness-density covariance" (pages 22-23), and we show results in the new Extended Data Figure 7.

Re: Comment 3, empirical evidence for clustering.

(Here I'm going to focus on the case where \beta_jj=\beta_jk for all species j and k, as this would eliminate any mechanism other than the novel one in this paper).

Their basic model relies on the assumption that as a species becomes rarer, they encounter conspecifics less, and still encounter roughly the same number of heterospecifics. This mechanism does not work if the number of heterospecifics increases (unless Janzen-Connell effects are important).

> **Response:** Yes, it happens indeed in this model for the case of stable dynamics that as a species becomes rarer, they encounter conspecifics less, and still encounter roughly the same number

of heterospecifics. However, this does not hold for unstable dynamics, as we will show below (see Figs. R1c,d,e, f below).

This mechanism does not work if the number of heterospecifics increases (unless Janzen-Connell effects are important).

Response: Yes, but in our model the number of heterospecific neighbours rather slightly decreases if abundance of a species increases (see Figs. R1e, f below). Note that the environment is homogeneous in our simulations.

I guess I'm still not convinced from their data that this is occurring.

Response: To clarify concern 3 we focus, as suggested by the reviewer, on the case where \beta_ff=\beta_fi for all species f and i (i.e., spatial effects are the only potential coexistence mechanism). Indeed, the reviewer is right, our coexistence mechanism is a specific form of positive fitness-density covariance and we show that it is related to subtly spatial patterns in the neighbourhood of individuals. To confirm this we apply scale transition theory (e.g., Chesson et al. 2005; Chesson 2012) and by using data of our simulations we show empirically that coexistence arises in a situation where "individuals of a common species are more likely to be near more trees in total", as demanded by the reviewer.

In short, we estimate the expected fitness $\lambda_{k,f}$ of an individual k of a focal species f (based on equations 1 and 7 in the manuscript), we show that the expected spatial average fitness $\overline{\lambda}_f$ has a value of one, and as a consequence, species f will enjoy a positive fitness-density covariance if the average of the expected individual fitness values $\lambda_{k,f}$ is larger than one (thus, the species is mostly located in "favourable" places where $\lambda_{k,f} > 1$). We then show analytically and empirically, based on data generated by our simulations, that these favourable places are indeed those where individuals of a rare species are more likely to be near fewer trees in total.

In more detail, equation 1 in the manuscript allows us to determine the **expected fitness $\boldsymbol{\lambda_{k,f}}$ of an individual** k of a focal species f for our macroscale model (eq. 7) (i.e., the expected contribution of individual k to the population after some defined interval of time):

$$\lambda_{k,f} = f(W_k) = r_f + s_f \exp(-\beta_{ff}\underbrace{(n_{kff} + \sum_{i \neq f} n_{kfi})}_{W_k}) \qquad \text{(R1)}$$

where $W_k = n_{kff} + \sum_i n_{kfi}$ is the **fitness factor** of individual k and n_{kff} and n_{kfi} are the number of conspecific and heterospecific neighbours of individual k within distance R, respectively. The fitness factor W_k is thus the total number of neighbours of individual k. The spatial average of the fitness factor over the entire plot is

$$\overline{W_k} = cN_f(t) + c\sum_{i \neq f} N_i(t) = cJ(t) \tag{R2}$$

where the factor $c = \pi R^2/A$ scales the number $N_f(t)$ of individuals of species f in the entire plot of area A to the expected number of individuals in the neighbourhood area πR^2 with radius R, and $J(t) = \sum_i N_i(t)$ is the total number of individuals in the plot. Given that the total number of individuals converges very quickly into equilibrium J^* (Extended Figure 5 and Supplementary Figures S1 and S2) we find for the **spatial average fitness** (i.e., the expected fitness of an individual k in the mean field model):

$$\overline{\lambda_f} = f(\overline{W_k}) = 1. \tag{R3}$$

The **average individual fitness** $\tilde{\lambda}_f(t)$ of a focal species f is the average of $\lambda_{k,f}$ over all individuals k of species f:

$$\tilde{\lambda}_f(t) = \frac{N_f(t+1)}{N_f(t)} = \frac{1}{N_f(t)} \sum_{k=1}^{N_f(t)} \lambda_{k,f} = \sum_{k=1}^{N_f(t)} \lambda_{k,f} \underbrace{\frac{1}{N_f(t)}}_{\nu_{f,k}} \tag{R4a}$$

A key ingredient of scale transition theory is that

$$\tilde{\lambda}_f(t) = \overline{\lambda_f} + \mathrm{cov}(\lambda_{k,f}, \nu_{k,f}) \tag{R5a}$$

where $\mathrm{cov}(\lambda_{k,f}, \nu_{k,f})$ is the **fitness-density covariance**. Since $\overline{\lambda_f} = 1$, species f has a positive fitness-density covariance if the average of $\lambda_{k,f}$ is larger than one. Thus, the species is mostly located in "favourable" places. Equation R1 shows that the expected individual fitness $\lambda_{k,f}$ will decrease if the fitness factor $W_k = n_{kff} + \sum_i n_{kfi}$ (i.e., the total number of neighbours of individual k) increases. Thus, a positive fitness-density covariance in our model means, as outlined by the reviewer, that individuals of a common species are more likely to be near more trees in total.

We now show this empirically based on the results of our individual-based simulation model. To this end we used the data of the last simulation steps (time step 5000). We estimated for each species the mean number of conspecific, heterospecific and total neighbours within distance R

and plotted it over abundance (Figure R1). This was done for stable dynamics (Extended Data Figure 5c) and unstable dynamics (Extended Data Figure 5a).

Additionally we used equations 4 and 5 in the manuscript to estimate the expected mean number of conspecific, heterospecific and total neighbours (i.e., $\overline{n}_{ff}$, $\overline{n}_{fh}$, and $\overline{n}_{ff} + \overline{n}_{fh}$, respectively) based on the empirical (power-law) relationship between aggregation and abundance.

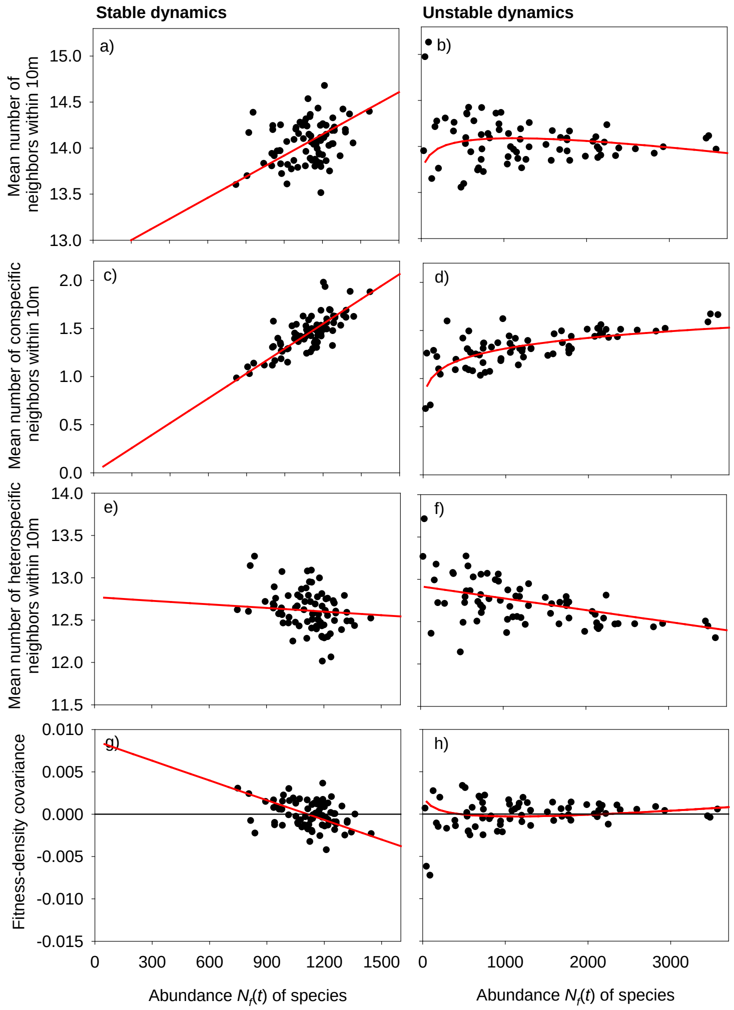

Figure R1. Mechanism underlying the fitness-density covariance in our model. Quantities involved in the estimation of the fitness-density covariance (eq. M11) for model simulations of the stable scenario (left) and the unstable scenario (right). The data were taken from time step 5000 of the simulations shown in

Extended Data Figure 5c (stable dynamics) and of Extended Data Figure 5b (unstable dynamics). Panels show the mean number $\overline{n}_{ff}+\overline{n}_{fh}$ of all neighbours (a, b), of conspecific neighbours $\overline{n}_{ff}$ (c, d), of heterospecific neighbours $\overline{n}_{fh}$ (e, f), and the resulting fitness-density covariance $\tilde{\lambda}_f-\overline{\lambda}_f$ (g, h), for all species plotted over their abundance. The red lines how the expected relationship based on equations M11 and M12. We fitted power law relationships $k_{ff}(N_f) = a\ N_{ff}^{p}$ to the data. For stable dynamics we find $k_{ff}(N_f) = 8.2$, $k_{fh}(N_f) = 0.905$ and for unstable dynamics $k_{ff}(N_f) = 3723/N_{ff}^{0.883}$ and $k_{fh}(N_f) = 0.890$.

Indeed we find for the stable simulations that individuals of more common species are more likely to have more neighbours within R = 10m (Fig. R1a), as predicted by equations 4 and 5, i.e., $\overline{n}_{ff}+\overline{n}_{fh}=\frac{\pi R^2}{A}(k_{ff}N_f(t)+k_{fh}\sum_{i\neq f}N_i(t))$ (red line in Fig. R1a). The reason for this is, as correctly noted by the reviewer, that as a species becomes rarer, they encounter conspecifics less (Fig. R1c), and still encounter roughly the same number of heterospecifics (Fig. R1e). However, there are no such trends for the data of the unstable dynamics, where the mean number of neighbours even slightly increases with decreasing abundance (red line in Fig. R1b).

Note that the number of heterospecific neighbours decreases deterministically with abundance, i.e., $\overline{n}_{fh}=\frac{\pi R^2}{A}k_{fh}\sum_{i\neq f}N_i(t)=\frac{\pi R^2}{A}k_{fh}(J(t)-N_f(t))$, since segregation is independent on abundance. Following equations R3 and R4a (or equations 6 and 7 in the manuscript) we can estimate the mean individual fitness $\tilde{\lambda}_f$ for each species and plot it over abundance N_f (Figs. R1g, h). Indeed, although noisy, we detect a positive fitness-density covariance for the stable dynamics (Figs. R1g), but not for the unstable dynamics (Figs. R1h) that agree with the analytical predictions.

We specify the coexistence mechanism of our model at lines 284-291), we provide the methods for this in a new methods section "Fitness-density covariance" (pages 22-23), and we include Figure R1 as Extended Data Figure 7.

Last time I said they could prove me wrong if they showed that there was a positive relationship between the number of nearby trees and the frequency of conspecifics. In figure R2, they showed the total number of conspecifics as the independent variable, rather than frequency.

There is a really important distinction here. Say that X is the number of conspecifics, and Y is the number of heterospecifics, and let's assume they are independent. Thus, if you calculate the regression line between conspecifics (X) and total individuals (X+Y), it will be:

cov(X,X+Y)/var(X)

=[cov(X,X)+cov(X,Y)]/var(X)

=var(X)/var(X)

=1.

I don't think this happens if you correlate frequency (i.e. X/(X+Y)) with total number of individuals (X+Y) (at least I ran a few simulations, and it didn't seem to). I'm sure that if there was no effect, one would still get some positive correlations for individual species just by chance, so I wouldn't find one or two graphs like R2 to be convincing. However, if there was a general trend across most species, then I'd be convinced.

Response: Yes, this figure did not help. It took us some time to understand that there was a misunderstanding in interpreting “frequency of conspecifics”. To show this we follow the illustration of scale transition theory presented in Chesson (2012) that describes the transition from local dynamics occurring within a location x to dynamics occurring at the landscape (i.e., the collection of all locations x). If we now replace the index *k* that identifies the individual by the index x that identifies the patch, the analogue of equation R4a (equation 4 in Chesson 2012) is:

$$\tilde{\lambda}_f(t) = \frac{N_f(t+1)}{N_f(t)} = \frac{\sum_x N_{x,f}(t+1)}{\sum_x N_{x,f}(t)} = \sum_x \lambda_{x,f} \underbrace{\frac{N_{x,f}(t)}{N_f(t)}}_{\nu_{x,f}}, \qquad \text{(R4b)}$$

given that the local dynamics at location *x* is governed by $N_{x,f}(t+1) = \lambda_{x,f} N_{x,f}(t)$. The quantity $\nu_{x,f}$ is the frequency of conspecifics at location *x*. From equation R4b it follows (equation 7 in Chesson 2012):

$$\tilde{\lambda}_f(t) = \sum_x \lambda_{x,f} \nu_{x,f} = \bar{\lambda}_f(t) + \mathrm{cov}(\lambda_{x,f}, \nu_{x,f}) \qquad \text{(R5b)}$$

Since in an analogous grid-based model the fitness factor W_x of individuals at location *x* would also increases with decreasing number of nearby individuals (i.e., $\sum_f N_{x,f}(t)$), we obtain a positive

fitness-density covariance if the number of nearby individuals is negatively correlated with the frequency $v_{x,f}$ of conspecifics.

However, as shown in equation R4b, the frequency of conspecifics is given by the number $N_{x,f}(t)$ of conspecifics at location x divided by the total number $N_f(t)$ of conspecifics in the landscape, but not by "# conspecific neighbours / total # of neighbours" as suspected by the reviewer. It should be "# conspecific neighbours / total # conspecifics in the landscape". Therefore the figure could not provide the desired result, but this is now shown in figures R1a,b based on the individual-based version of the equation (i.e., eq. R5a).

The variance to mean ratios were somewhat convincing (while they said the values are "mostly" above 1; however, it looks like 1 is in the middle 50% in many plots, and it is within the middle 90% in most plots). This is enough that I withdraw that comment. However, this still does not prove to me that when a species becomes rare, it will have fewer individuals to compete with.

Response: This is now shown in Figures R1a, b. Indeed, in the stable simulations when a species becomes rare, it will have fewer individuals to compete with (Fig. R1a). However, this was not the case for the unstable dynamics (Fig. R1b).

Like I said before, if rarer species don't start experiencing less competition due to decreased crowding, the mechanism will not work. This seems like a really weird mechanism, one I wouldn't necessarily expect to work like they say it does. If they can show convincing evidence that this mechanism is operating, then I think that this is kind of a big deal, one deserving of a place in Nature E&E. But, if they can't, then I think what they have is basically a modeling paper, and one that is based on assumptions that seem dubious.

Response: Yes, as show above (Fig. R1a,b), the coexistence mechanism is indeed that rarer species start experiencing less competition due to decreased crowding. This mechanism is entirely emerging due to spatial small-scale patters caused by two spatial mechanisms: an aggregation mechanism in the placement of recruits and local competition (and can be explained by our individual-based model equations and scale-transition theory). We therefore hope that our study will stimulate interest in our approach that combines forest inventories, spatial point process statistics, and dynamical theory.

Re: Comment 1, the results are not novel.

I do think that what is occurring is a fitness density covariance. Intraspecific competition is the same as interspecific competition (at least in the neutral model). This means it would not be a variation-independent mechanism. The environment is homogenous, suggesting that a spatial storage effect is not occurring, and they are basically identical, suggesting that a spatial relative nonlinearity is not occurring. Instead, certain factors cause species to become crowded (i.e. have high density), which lowers survival (i.e. have low fitness). If you randomly rearranged all individuals, the effect would go away. Thus, this is a fitness-density covariance.

> **Response:** Yes, you are right. We have applied scale transition theory to our case to estimate the expected fitness-density covariance of our simulations (Figs R1, g, h).
>
> We specify the coexistence mechanism of our model now at lines 284-291, we provide the methods for this in a new methods section “Fitness-density covariance” (pages 22-23), and show results in Extended Data Figure 7.

That said, I see their point on the rest of the response on this one, and this isn't a deal-breaker of a concern.

> **Response:** We are happy about this.

Re: Comment 6, math problem

The authors assumed that B_f is a constant, when it actually depends on the densities of every species in the community. They argue that in a diverse community, any one species will have little impact on B_f. I don't think that means it can be ignored. The impact of one species on B_f will be roughly inversely proportional to the number of species in the community. However, any stabilizing mechanism will also be inversely proportional to the number of species in the community, so those little impacts can matter.

I agree that in a stable community, B_f will remain largely stable. It is during an invasion that I worry. For example, imagine a 50 species community, where species A and B both have the same disperser (thus allowing those two to be dispersed to unusual microhabitats). If A became rare and B did not change in frequency, it should be easier for A to bounce back (as there is less competition in the microhabitat). However, if A becomes rare and B becomes twice as frequent, then A will have a much harder time bouncing back.

Response: This is a good point. To investigate this issue in more detail we took up an idea from your AmNat paper (Stump 2017). We already had derived a matrix of the (relative) individual level interaction coefficients β_{fi}/β_f for Extended Data Figure 8 based on a two-dimensional niche space. Our simulation results based on these interaction coefficients (Extended Data Figure 8a, b) suggest that there is an initial burn-in phase where the community quickly eliminates “weak” species and experience an increases in the abundance of “strong” species.

Looking closer to our simulation data shows that “weak” and “strong” is closely related to the **central niche effect** introduced in your paper. To show this we represented a species in the niche space plots by a circle proportionally to its abundance (Fig. R2 below and new Supplementary Figure S3). In this analysis we used two scenarios: recruits scatter around conspecific adults (unstable dynamics) and recruits scatter around random cluster centres (“stable” dynamics).

When doing this for different simulation steps (i.e., 4, 16, 64, 256, 1024, 4096) we find for both scenarios that species located in the centre of the niche space loose rapidly abundance and may even go extinct quickly in the unstable scenario (Figs. R2, R3). In both scenarios the **central niche effect** (i.e., abundance is related to the distance of the species location in niche space to the centre of the niche space; Fig. R3) is operating in the initial burn-in phase.

However, the results of the stable scenario do not indicate operation of the **common competitor effect** since we observe that species which are located close to each other in niche space (i.e., “similar species”) have similar abundance during the entire simulation (e.g., t = 4096 for “stable” dynamics). In contrast, results of the scenario of unstable dynamics indicates that similar species maintain initially similar abundances, but after the burn-in phase when most weak species go extinct, the **common competitor effect** appears (Fig. R2) since the abundances of similar species become more and more dissimilar during time. At the end only one of the cluster of similar species may survive. Here the **community redistribution effect** is operating (Figure R4).

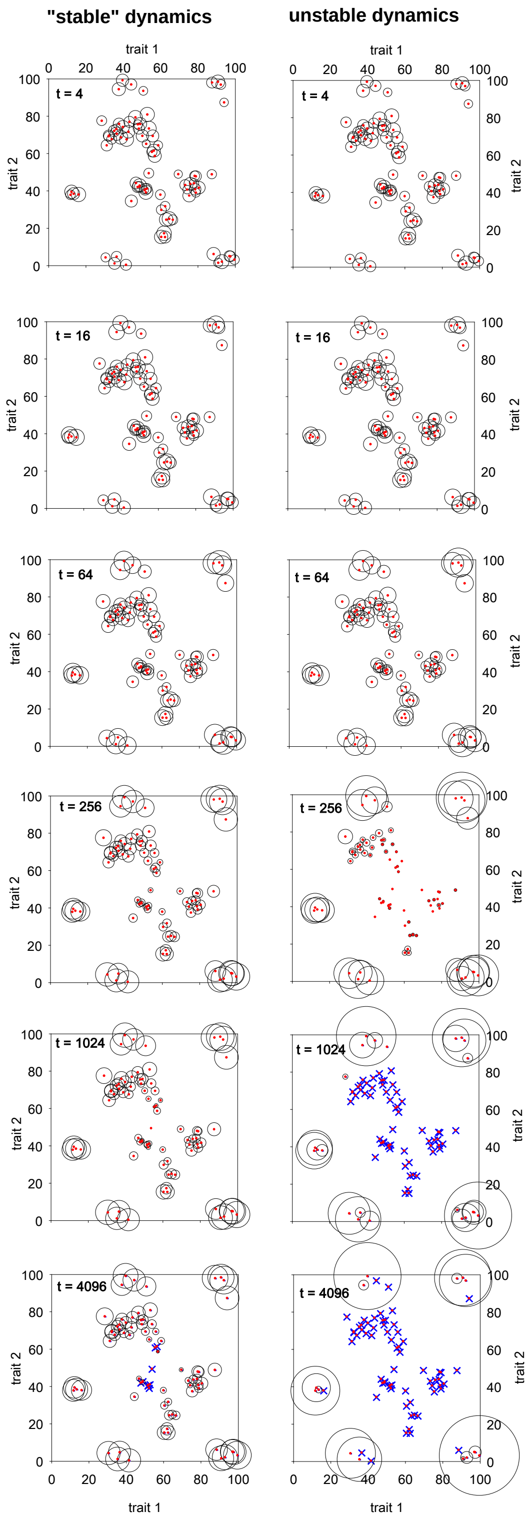

Figure R2. Dynamics of the community visualized in the niche space for the "stable" and the unstable scenario shown in Extended Data Figure 8. We determined the matrix of the (relative) individual level interaction coefficients β_{fi}/β_f by representing each species as a point in two-dimensional niche space, and $\beta_{fi}/\beta_f = 1 - d_{fi}$ was proportional to the Euclidean distance d_{fi} between species f and i (as in Stump 2017). The area of the disks is proportional to abundance, red dots show the location of the 80 species, and blue crosses mark extinct species.

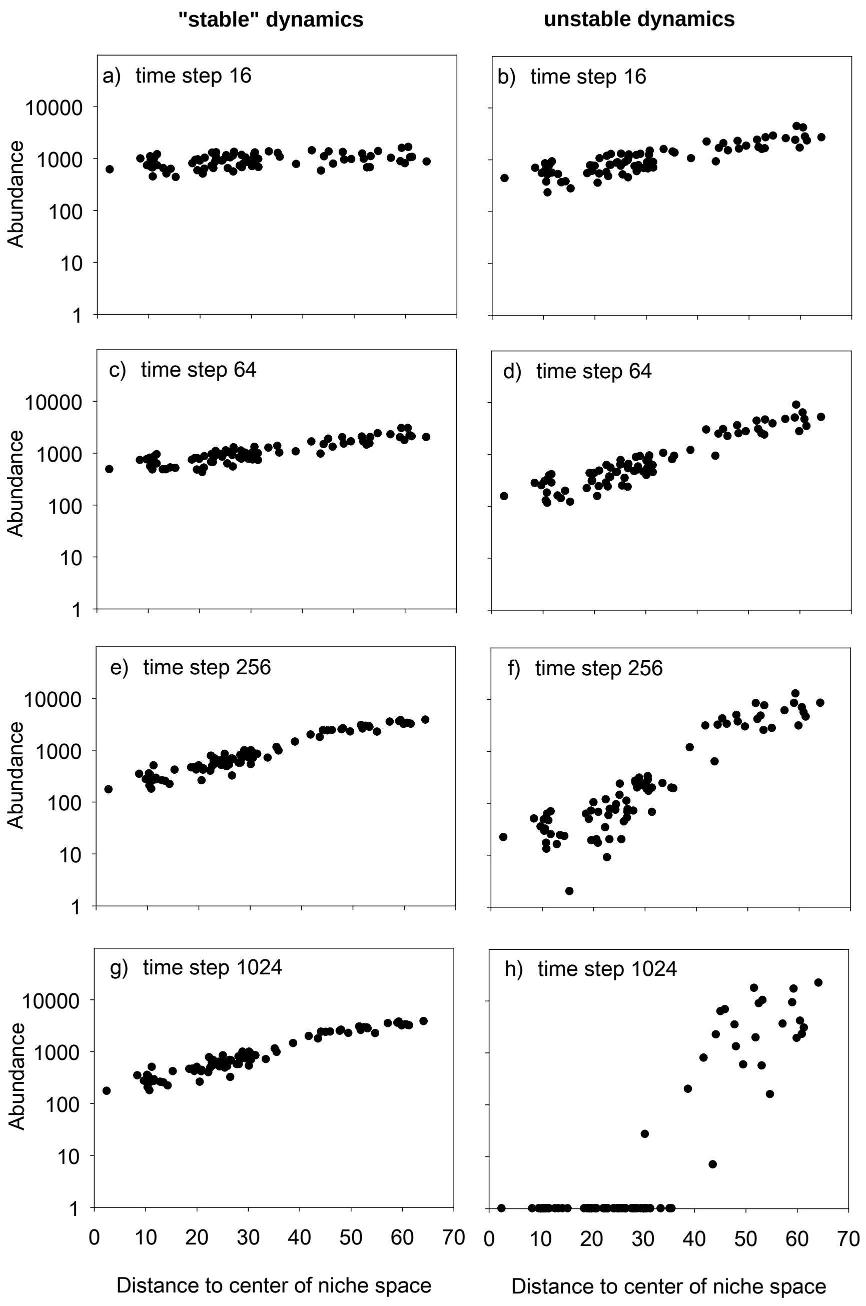

Figure R3. Emergence of the central niche effect in our model simulations shown in Fig. R2. We plotted the abundance over the distance to the centre of gravitation of the species in niche space. Indeed, the abundance increased strongly with distance from the centre. For the scenario of unstable dynamics, species with a distance below 40 went extinct very quickly (They are shown having abundance of one).

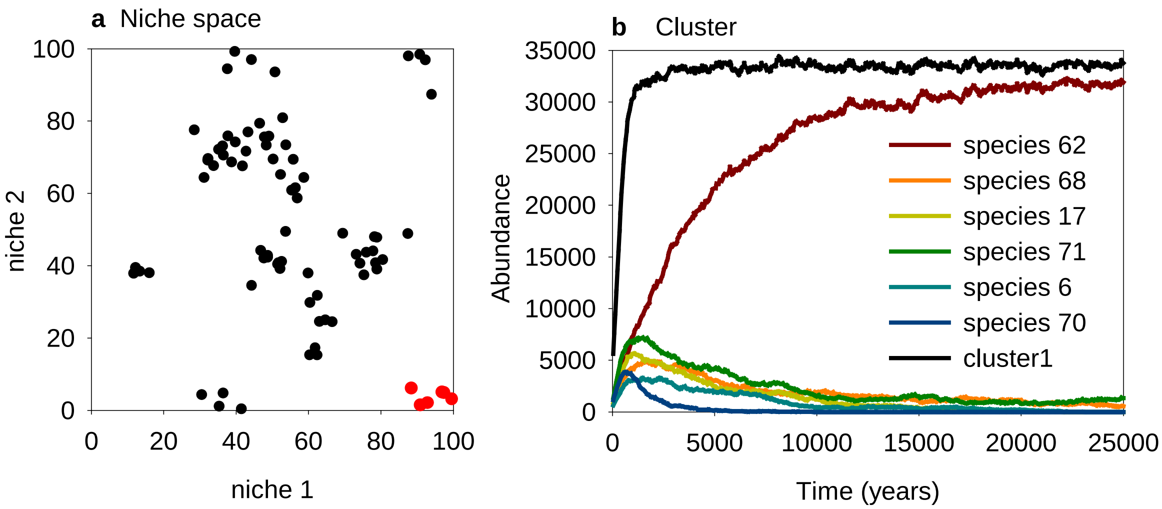

Figure R4. Emergence of the community redistribution effect in the model simulations of unstable dynamics shown in Fig. S2s. (a) We analysed a clusters of similar species (i.e., species are located close to each other in niche space indicated by red colour), (b) time series of the abundances of individual species in the cluster and of the entire cluster. After the burn-in period of some approximately 1000, the joined abundances of species within the cluster remains constant, but the abundances of the component species change. Thus, we observe a destabilizing community redistribution effect where a species is replaced by a close competitor.

These insights now inform us about the question if B_f will remain largely stable. For the "stable" simulations the values of B_f shifts only during the burn-in phase where the community adjusts to the initial condition by means of the central niche effect. Once this phase is passed (in our case some 200-300 time steps), dynamics are stable and B_f is basically constant. In contrast, the scenario of unstable dynamics shows during the burn-in phase a very quick and strong initial reorganization of the community due to the central niche effect (say up to time step 256) where weak species go extinct and strong species increase in abundance. During this period we also observe larger shifts in the values of B_f (see Extended Data Figure 8g), but afterwards B_f remains fairly constant. This is because the **common competitor effect** does not impact B_f much since one species of a cluster of species with similar niches gains abundance on expense of the other species in the cluster (i.e., a destabilizing community redistribution effect; Fig. R4b). Therefore changes in B_f are very slow after the burn-in period.

That means that the assumption of approximately constant B_f is an issue especially during the burn-in phase, for cases where rapid environmental change may force a re-assembly of the community, or if one of more “superspecies” are invading the community and force a reassembly. However, once the community has settled down in a quasi-stationary state, the assumption of a constant value of B_f is a reasonable assumption.

This is now mentioned in the manuscript (lines 408-413) after the detailed description of the summary function B_f. We moved the methods section on testing this assumption to Supplementary Text, but expanded it by analysing our simulation results using methods presented in Stump (2017). The central niche effect, the common competitor effect and the community redistribution effect are shown in the new Supplementary Figures R3-R5.

I also don't usually sign my reviews, but since I'm going to talk about my own work, this is Simon Stump. I reran a slightly modified version of the code for Stump (2018, AmNat), this time using communities of between 19 and 28 species (mean 24, using 6 simulations, starting with a species pool of 50). The average invader growth rates were reduced by between 30% and 75% when species had species-specific interaction coefficients vs. the diffuse case (mean 50%). So, I don't think this was purely because 10 species was too species poor. I'm not sure how many you need to consider a community "diverse," and if 24 is not enough, I can try to run more, but I suspect the answer will be the same.

Response: This is a good question to be answered in forthcoming studies. In any case, we showed that the three effects of nondiffuse competition (i.e., the central niche effect, the common competitor effect, and the (negative) community redistribution effect) occur also in our model if we assume non-diffuse competition. This was most clearly shown for the case where the relative individual level interaction coefficients β_{fi}/β_f were derived from the species locations in a two-dimensional niche space. However, once the community is pruned back by the central niche effect, subsequent changes of the relative abundances of species (due to the common competitor effect and the community redistribution effect) may not lead to larger changes in the values of B_f as shown in figures R2, R3 and R4.

All of this said, their simulations are semi-convincing. If they can fix the issues with comment #3, then I don't my concerns on comment #6 this should prevent this paper from being published. I would only ask that they acknowledge this as a potential problem (and, I understand if they do not want to cite my work, I will not hold it against them if they do not).

Response: We fixed the issue with comment #3 and analyse and discuss the consequences of the three effects of non-diffuse competitor found in Stump (2017) on the values of B_f in the Supplementary Text and Supplementary Figures S3-S5. In interpreting the results of Extended Data Figure 8 (now Supplementary Figure S2) we acknowledge that the central niche effect during the burn-in period, during rapid environmental change, or during invasion of “superspecies” may lead to non-constant values of B_f.

Reviewer #1's comments on reviewer #2's initial report: "on ecological equivalence"

I agree with the reviewer that the species were not "equivalent," and I'd also say that they are not "neutral." Neutral would imply no species differences (and hence no stabilizing mechanisms or fitness differences). But, these species are different, as their seeds disperse into different locations; I agree that it could be due to animal dispersers, but if it is, then there has to be some difference between the seeds that causes the animals to disperse them into different areas (again, see my commentary about the model in Chesson 2000 TPB, if offspring are dropped in clusters, this won't allow coexistence unless the clusters are dropped in different areas). Really these communities are full of species that are identical other than seed dispersal. I'd worry that calling them "neutral" is misleading for the same reason as before, I'd suggest that the authors try hard to come up with a better term. "Nearly neutral" might work, as they are neutral except for this difference in dispersal (or maybe "otherwise neutral"?). Like, they need to make it clear that the species are different, it's just that inter- and intraspecific competition are the same.).

Response: This is a good point but difficult to resolve. We tried hard to come up with a better term to replace the terms “ecological equivalence” or “neutral”.

First we notice that classic neutral models assume neutrality at the level of individuals. All individuals have the same chances of reproduction and death regardless of their species identity (i.e., all individuals have the same expected fitness). If we use the spatial information, our “neutral” model version does not fulfil this condition since the birth and death prospects of an individual are influenced by the total number of neighbours in their close surrounding (i.e., a negative density dependence).

Nevertheless, in the “stable” model version (were recruits aggregate around randomly placed cluster centres) all individuals follow exactly the same stochastic rules, do not distinguish

between con- and heterospecifics, and the placement of recruits follows for each species exactly the same stochastic rules: individuals are placed randomly, but in small groups.

Thus, this model version is **symmetric** at the species level as species labels are exchangeable and all species behave in exactly the same way and therefore belongs to the class of "**symmetric models**" (such as e.g., the model of Volkov et al. 2005: Nature and Jabot and Chave 2011). These models are nonneutral in a particular sense: species with different abundances have different demographic rates. However, any two species with exactly the same abundance are strictly equivalent demographically. In contrast to the models of Volkov et al. (2005) or Jabot and Chave (2011) that imposed the rare species advantage, it emerged in our model under certain circumstances from identical model rules at the individual level. Note however that some authors do not make a distinction between such symmetric models and neutral models.

We therefore tried to represent these subtleties adequately in the text. We avoid the term "neutral" and explain that in this symmetric model version all species have the same parameters, follow the same stochastic rules, and all individual compete identically (lines 248-251).

I'd also suggest removing "(including neutral communities)" from line 52, and not replacing it with the new term - unless the authors are willing to explicitly describe the term, then the readers won't know what they mean, and it will come across as false advertising.

Response: ok.

"the use of the number of individuals for crowding index needs explanation"
If basal area works better, than I'm not sure why they didn't use it here. At the very least, I'd like them to at least mention that a model using basal area gave similar results.

Response: Well, we decided to outline our framework with the simplest case of crowding indices that consider only number of neighbours within distance *R*. For this reason we also removed in the second version of the manuscript the distance weighting of the crowding indices that we used in the original submission.

However, as proposed we briefly mention that the analytical model can be extended by redefinition of the indices of aggregation and segregation (k_{ff} and k_{fh}) to consider also basal area and that it produces similar results (lines 316-320).

The number of neighbours within distance R is related to the widely known Ripley's K in a straight-forward way , whereas the derivation of the indices of aggregation and segregation (k_{ff} and k_{fh}) is more complex for distance weighting, but results otherwise in the identical equations (e.g., eqs. 6-11).

Using basal area requires the introduction of a growth mechanism for tree sizes in the simulation model, which would complicate the presentation of our framework considerably. This will be subject to forthcoming studies.

Reviewer #3 (Remarks to the Author):

This study examined how local competition and spatial distribution affect coexistence of tree species by using spatial point pattern analysis and coexistence theory. The authors combined spatial data of 9 forest plots, spatial statistics, and dynamic models to show that spatial clustering can promote species coexistence. Because of the importance of the topic, this study is of interest to people in this research area. However, I feel the manuscript has some weaknesses to be addressed.

Response: Thank you for your constructive comments and many helpful suggestions that helped us to improve the presentation of our study and to better embed it into existing theories.

Major comments:

1. What is the novelty of this study? The authors wrote that the "mean-field approximation" is not enough to understand coexistence of diverse tree species (L75), but previous studies already showed conspecific clustering promotes species coexistence by using pair approximations and numerical simulations (e.g., "According to theoretical models, conspecific spatial clustering is recognized as a critical mechanism for reducing competitive exclusion and promoting diversity." in Seidler & Plotkin 2006 PLoS Biol). I think the authors should emphasize the strength of this study (the combination of spatial data of trees, spatial statistics, and dynamical theory) more carefully in Abstract and Introduction.

Response: This is a good point. Indeed, the combination of spatial data of trees, spatial statistics, and dynamical theory is a strength and novelty of our study. From a technical side, we present an approach that allows to link spatial processes at the individual-level to community dynamics. This is now outlined more carefully in the Abstract (line 45-46) and Introduction (page 4, lines 68-69). An additional novelty of our study is the discovery of a mechanism that allows

conspecific aggregation and heterospecific segregation to promote coexistence in otherwise neutral models (now mentioned at lines 51-53 in the abstract).

The hypothesis that conspecific aggregation and heterospecific segregation can promotes coexistence is not new, but was rejected based on models that generated conspecific clustering based on neighbourhood dispersal. In agreement with our results, intraspecific clustering and interspecific segregation does in this case not lead to coexistence. Klausmeier & Tilman (2002) nicely summarized the current thinking about this issue:

> "Since local dispersal can cause intraspecific clumping and interspecific segregation, one might think that these explicitly spatial phenomena could stabilize otherwise neutral competitors. However, (…) the positive effects of resident clumping and interspecific segregation are completely negated by the negative effect of invader clumping".

This is now mentioned at page 13 (lines 271-276).

2. The connections to previous studies are not clear. The authors wrote about "a stabilizing mechanism" (L51-52), but is this the term used in Chesson's coexistence theory (i.e., stabilizing vs. equalizing mechanisms)? I am wondering whether the mechanism of coexistence is "growth-density covariance" of Chesson's coexistence theory. If not, what is the difference? Is it spatial relative nonlinearity or the spatial storage effect?

Response: You are right, the mechanism of coexistence in our model is a form of growth-density covariance that produces a rare species advantage. However, the underlying individual-level mechanism that produces this behaviour is novel. As suggested, we make the connections to previous studies and especially to the scale transition theory of Chesson explicit (line 284-287) and we added a methods section on the fitness-density covariance (lines 467-499). This allows us to better embed our framework into existing theory and to point to our novel contribution by revealing the individual-level mechanisms required to produce stability in our model.

The authors often cited papers on food web theory (e.g., May 1972), but I think it (with several trophic levels) is different from coexistence theory (in a single trophic level).

Response: We partly agree. Several of the cited papers on food web theory deal also with the special case of competitive communities (e.g., Saavedra et al. 2017, Stone 2018 and Servan et al. 2018). However, we see your point and removed some of these studies.

3. Using empirical data to understand stable coexistence of species-rich communities is an interesting and important topic. For example, Ushio et al. (2018) Nature used time-series data of fish species for estimating interaction strength (and network structure) as well as stability of a Jacobian matrix. Is it possible for the authors to use spatial data for estimating network structures as well as stability of the whole community? It would be great if the authors can add discussion.

> **Response:** This is a good point. Unfortunately, with our data on hand and assuming approximate equilibrium we still have one unknown factor. We need either additional information on the relative individual-level interaction coefficients $ß_{fi}/ß_{ff}$ or information on the absolute value of $ß_{ff}$ (see eqs. 8 and 6).
>
> A paragraph of the manuscript is dedicated to the issue of using empirical data to understand stable coexistence of species-rich communities (lines 228-242). You found this an "interesting" and important topic (see your major comment 3). The information to estimate equation 9 allows us also to estimate the Jacobean matrix **J** of the spatial multi-species model. We presented and estimated the Jacobean matrix **J** in the first version of the manuscript, but this was criticized by the reviewers and we removed it.
>
> We point now in the manuscript to the intriguing possibility to use advanced versions of our approach to use spatial data together with additional information that is available for estimating network structures as well as stability of the whole community (lines 320-324).
>
> For your information, the Jacobean matrix **J** of the spatial multi-species model yields
>
> $$\mathbf{J} = \underbrace{\begin{pmatrix} d_1 & 0 & \cdots & 0 \\ 0 & d_2 & \cdots & 0 \\ \vdots & \vdots & \ddots & \\ 0 & 0 & \cdots & d_S \end{pmatrix}}_{\mathbf{D}} \underbrace{\begin{pmatrix} 1 & \frac{\alpha_{1h}}{\alpha_{11}} & \cdots & \frac{\alpha_{1h}}{\alpha_{11}} \\ \frac{\alpha_{2h}}{\alpha_{22}} & 1 & \cdots & \frac{\alpha_{2h}}{\alpha_{22}} \\ \vdots & \vdots & \ddots & \\ \frac{\alpha_{Sh}}{\alpha_{SS}} & \frac{\alpha_{Sh}}{\alpha_{SS}} & \cdots & 1 \end{pmatrix}}_{\mathbf{A}}$$
>
> where **D** is the diagonal matrix with elements $d_f = -(1 - r_f) N_f^* \alpha_{ff}$ and **A** is the interaction matrix.

4. It will be great if the authors can upload programing codes for statistical analyses and model simulations so that readers can replicate the study.

Response: We uploaded the source code for our model that also included estimation of the spatial statistics as supplementary information.

Minor comments:

L44-46: Satisfying for whom? There are many mechanisms that promote coexistence (e.g., self-regulation, higher-order interactions, temporal & spatial storage effect & relative nonlinerity, growth-density covariance, diversity of interaction types, etc.), so it may be better to avoid the subjective expression.

Response: We replaced “satisfying” by “fully”.

L46-47: There are already many studies that considered spatial dynamics with local competition. It may be better to emphasize the methodological novelty of this study here.

Response: This is true. We emphasize here instead the difficulty to link individual-level processes to community dynamics.

L48: Timescales of what? I thought "multiscale" means multiple biological scales (individual-level vs. population-level) or multiple spatial scales based on the sentence in L46-47, but it seems that the authors are talking about multiple timescales.

Response: You are right, this is not understandable in the brief form here in the appendix. We actually mean both: multiple biological scales and separation of time scales where mesoscale patterns (e.g., the spatial patterns) converge very quickly into a quasi-stationary state whereas macroscale state variables (abundances) are comparably slow. The latter allow us to ignore the dynamics of the mesoscale quantities, but to derive nevertheless equations for the macroscale model dynamics. This powerful technique is called in physics adiabatic approximation. We removed this phrase fom the abstract, but it is explained in the text (lines 84-91).

L50-52: I think previous studies showed the similar conclusion. It would be great if the authors can provide a better explanation for readers to easily understand the novelty of this study.

Response: Done.

L51-52: "Stabilizing mechanism" for "neutral communities" sounds strange. Do the "neutral communities" mean communities with no niche difference?

Response: Yes, this is true. We outline now that we identified a mechanism that can produce spatial patterns that lead to a rare-species advantage and coexistence of otherwise neutral competitors (lines 51-53).

L60: Is Darwin (1859) appropriate here? I think its main focus is the origin of species diversity. Hutchinson (1959, 1961) may be more suitable for the maintenance of species diversity.

Response: Ok, we replaced the reference by Hutchinson (1961).

L62: I think references for food web dynamics (11, 12, 13, 15: May 1972, Allesina & Tang 2012, Stone 2018, Servan et al. 2018) may be irrelevant here. Those studies considered trophic interactions whereas the authors' focus here is coexistence of competitors, right?

Response: We partly agree. Yes, May 1972 is not part of modern coexistence theory, but several of the "network" papers analysed also coexistence of competitive communities. We see your point and removed here May 1972 and Allesina & Tang (2012).

L65-66: It may be better to point out here that there are many mechanisms proposed to explain the observed diversity. And please add the explanation why the authors think they are not satisfying.

Response: We removed this phrase and state that results of theoretical models indicate that stable coexistence is difficult to reach in large communities (line 61-62).

L70: What is "the dynamic theory of communities"?

Response: we replaced this term by "coexistence theories of species-rich communities".

L72: Again, I think theory of food web dynamics proposed by May (1972) is not a part of modern coexistence theory.

> **Response:** Ok, we removed this reference.

L75: There are theoretical studies that considered pair-approximations for community dynamics (e.g., Klausmeier & Tilman 2002). Is the pair-approximation not enough?

> **Response:** Well, we state at lines 68-73 that the analytical models that form the basis of most of modern coexistence theory often neglect spatial patterns. Yes, pair-approximations or moment equation models for community dynamics are one possible approach to study the impact of spatial patterns on species coexistence. We therefore mention them now more explicitly as example (line 76-79).

L94-98: This is a nice summary of this study. It will be nice to revise the abstract as like this part.

> **Response:** Thank you! We rewrote the abstract completely.

L102: Where is reference 27?

> **Response:** We corrected the numbering of the references.

L107, legend of Fig. 1, L158-159: It is confusing that the authors sometimes use n_{kfi} and sometimes n_{kfh}. In addition, the authors wrote the definition of h in L158-159. Please be consistent and provide the definition in L107.

> **Response:** Well, n_{kfi} refers to the number of individuals a specific species *i* in the neighbourhood of the individuals of the focal species *f* (e.g., in eq. 1a) whereas n_{kfh} refers to all heterospecifics. To clarify this we added the definition of n_{kfh} together with the definition of the subscript "h" to the definition of n_{kff} and n_{kfi} (lines 105-109).

L116: "Independent distributions p_x and p_y" sound confusing. Please add more explanations why the authors introduce them here.

Response: We revised this part. Actually, we do not need to refer here to the independence between the two indices because we extract this property in a later step from the observed data (line 116-117).

L121-122: Please add justifications why the authors can use phylogenetic similarity for the relative competition strength. And how did the authors estimate beta_{ff}?

Response: We added a brief justification ("This is an established approach in species rich communities[21,28] to approximate niche differences in the absence of other data") (lines 121-123).

Our spatial analysis require only data on beta_{fi}/ beta_{ff}, but not on beta_{ff} alone. However, in the simulations of the individual-based model we adjusted beta_{ff} to obtain approximately the same density of individuals as the BCI plot (this is mentioned at lines 539-543).

L125-126: Why did the authors mention that variance-to-mean ratios are not one here? Is it important?

Response: Not really, we removed this.

Fig. 1b, c: Why did the authors use circles and arrows with different sizes and widths, respectively? It seems not informative, but confusing.

Response: Ok, the different size of the circles does not contain information, we made them all the same size. However, we keep the different width of the arrow of since they symbolize different interaction strength.

Legend of Fig. 1: competitive effect beta_{fi}/beta_{ff} → competitive effect beta_{fh}/beta_{ff}

Because the authors used n_{kfh} instead of n_{kfi} here.

Response: No, it must be beta_{fi}/beta_{ff} as written because the "interaction" index $n_{kfß}$ shown in panels **c** and **f** is based on the pairwise individual-level interaction coefficients : $n_{kfß} = \sum_{i \neq f} (\beta_{fi} / \beta_{ff}) n_{kfi}$.

Solid lines show → Blue lines show

Because both red and blue lines are solid in Fig. 1d-f.

Response: This is true, we changed it accordingly.

Why did the authors choose dbh > 10cm? What will happen when we choose different values (e.g., dbh = 1cm, 5cm, or 20cm)?

Response: The 10 cm value is well established in the analysis of the CTFS-ForestGEO data sets as threshold for larger trees. We explained this in the methods (lines 353-356). The 10cm threshold excludes most of the saplings and enables comparisons with previous spatial analyses (e.g., references 20, 34, 48, 49).

L131-133: Does this mean beta_{fi} are the same for all heterospecific species and the phylogenetic similarity (L121-122) is not necessary anymore?

Response: No, the detailed values of $ß_{fi}/ß_{ff}$ are still needed, they are condensed into the quantify B_f (see equation 12), which will in general differ among species.

L133: Why important?

Response: We removed the "important"

Using empirical data to understand stable coexistence of species-rich communities is an interesting and important topic

L142: I was confused: is this contradicting L125-126?

Response: No, the 5-year mortality rates of trees > 10cm dbh observed in the forest plots is low (about 0.1 per 5 years), as is the corresponding per capita reproduction rate r_f. In this case we can approximate the negative exponential function in equation 1a by a linear function (i.e., obtaining Lotka-Volterra equations). Integration of equation with the linear approximation does

not yield the factors γ_{ff} and $\gamma_{fß}$, which means they must be one for high survival. This is explained in the methods (lines 416-418).

The nonlinearity matters only for smaller survival rates, and the magnitude of the effect is then captured by the factors γ_{ff} = ln(1+b_{ff} $ß_{ff}$)/(b_{ff} $ß_{ff}$) and $\gamma_{fß}$ = ln(1+$b_{fß}$ $ß_{ff}$)/($b_{fß}$ $ß_{ff}$) where b_{ff} and $b_{fß}$ are the variance-to-mean ratio of the gamma distribution of the crowding indices n_{kff} and $n_{kfß}$, respectively.

An alternative explanation: a high survival rate requires in the macroscale model (eq. 7) a small value of $ß_{ff}$ (say 0.0075) to match the overall tree density of BCI (i.e., some 21,000 individual/50ha). Since the variance-to-mean ratios do usually not exceed values of 4, the values of γ_{ff} and $\gamma_{fß}$ will be close to one.

L163-166: Why did the authors assume that heterospecific competition is always weaker than conspecific competition? It is possible for heterospecific competition to be stronger than conspecific competition, it may cause priority effects of community assembly (e.g., Fukami 2015, Ke & Letten 2018).

Response: You are right, this is not needed. In fact later we see that our model also govern the case of $\alpha_{fh} > \alpha_{ff}$.

Legend of Fig. 2: Why did the authors choose R = 10m? What will happen when we choose different values (e.g., R = 1m, 5m, or 20m)?

Response: This was explained in the methods, lines 406-409. Statistical analyses with individual-based neighbourhood models based on neighbourhood crowding indices showed that the performance of trees depends on their neighbours within R = 10 to 15 m. We therefore estimated all measures of spatial neighbourhood patterns with a neighbourhood radius of R = 10 m. Analyses with R = 15 or R = 20 gave very similar results. This is now mentioned in the methods (lines 381-385). Very small neighbourhoods are not recommended since then the number of neighbours will be very small.

And what will happen when the authors include rare species with less than 50 individuals?

Response: The problem is that we cannot reliably estimate the indices for aggregation and segregation for small individual numbers. We will observe large noise due to the low

abundances. This can be observed in the individual-based model simulations (e.g., Extended Data Figure 5-7 where we showed also results of low abundance species).

L195-200: This explanation about Eq. 6b may be moved to L174.

Response: This is true. We moved this explanation.

L205: N_i(t)] → N_i(t))]

Response: Thank you for noticing this type, we corrected it.

L206-208: Is it possible to assume that the number of recruits depends on neighborhood competition. What will happen in this case?

Response: Yes, our analytical approach applies to a broad range of models. In the terminology of scale-transition theory (Chesson 2012), it has three core ingredients: the "fitness factor" $W_k = n_{kff} + Bf\, n_{kfh}$ of individual k, the negative exponential fitness function $f(W_k) = \exp(-ß_{ff}\, W_k)$ (it could also be a linear function), and the gamma distributions p_x and p_y of the terms n_{kff} and n_{kfh} of the fitness factor. As long as these three ingredients are justified by the data, our analytical approach will also apply to different macroscale models and only the definition of the carrying capacity K_f will change; all other equations are maintained. Our approach should also work for different fitness functions and distributions p_x and p_y but may then lose its analytical tractability.

We now mention this (lines 200-201) and we added a Supplementary Table S3 to provide the definition of K_f for several alternative models.

L216: Why interesting?

Response: We removed this term.

L232-233: Is this because there are no alternative stable states and Allee effects (e.g., Barabas et al. 2018, Schreiber et al. 2019)?

Response: We guess so, this is now mentioned at lines 220-221.

L254: Why interesting?

Response: We keep the "interesting" here because this section outlines the possibilities of using empirical data to understand stable coexistence of species-rich communities. You found this an "interesting" and important topic (see your major comment 3).

L295-296: What is the negative feedback between clustering and abundance? Increasing abundance promotes clustering, and clustering reduces the growth rate? If so, why does this cause instability?

Response: No, decreasing abundance promotes clustering, and stronger clustering reduces the growth rate. However, we removed the term "negative feedback" and refer to an emerging negative relationship between clustering and abundance (lines 290-291).

L298-299: This seems quite important. What about explaining this in Abstract and Introduction?

Response: Thank you for this suggesting, this is now explained in the abstract (lines 51-53).

L318-325: I think spatial conspecific clustering itself is not a novel mechanism for species coexistence and it will be great if the authors can revise this paragraph by acknowledging previous studies more carefully.

Response: We revised this paragraph accordingly (lines 292-297). See our response to your major comment 1.

L321-322: Chesson's framework states that species should be different enough for niche differences but similar enough for fitness differences, so this statement seems confusing.

Response: We revised this section (lines 292-297).

L332: Timescales of what?

Response: We removed the reference to the adiabatic approximation (i.e., separation of timescale).

L341-345: Please check previous studies carefully: is it really overlooked so far?

Response: Ok, we restated this phrase (lines 312-315).

L356-357: Where is the result of this statement?

Response: We completed the phrase (line 328).

L414: N_i(t)] → N_i(t))]

Response: We corrected this typo.

L620-621, 636-637: References 9 and 17 are the same (Levine et al. 2017).

Response: We corrected this.

Decision Letter, third revision:

15th December 2020

*Please ensure you delete the link to your author homepage in this e-mail if you wish to forward it to your co-authors.

Dear Thorsten,

Your manuscript entitled "Consequences of spatial patterns for coexistence in species rich plant communities" has now been seen by the same two reviewers, whose comments are attached. The reviewers have raised a number of concerns which will need to be addressed before we can offer publication in Nature Ecology & Evolution. We will therefore need to see your responses to the criticisms raised and to some editorial concerns, along with a revised manuscript, before we can reach a final decision regarding publication.

It appears that there are just a few remaining technical issues standing in the way of publication, most importantly that raised by reviewer 1: whether covariance is generally positive, or at least more positive for high-abundance species than low-abundance species--reviewer 1 suggests a specific statistical test for this but leaves the door open for you to provide an alternative. But we will need to

see this aspect addressed before we can offer to publish the manuscript.

We therefore invite you to revise your manuscript taking into account all reviewer and editor comments. Please highlight all changes in the manuscript text file [OPTIONAL: in Microsoft Word format].

We are committed to providing a fair and constructive peer-review process. Do not hesitate to contact us if there are specific requests from the reviewers that you believe are technically impossible or unlikely to yield a meaningful outcome.

When revising your manuscript:

* Include a “Response to reviewers” document detailing, point-by-point, how you addressed each reviewer comment. If no action was taken to address a point, you must provide a compelling argument. This response will be sent back to the reviewers along with the revised manuscript.

* If you have not done so already please begin to revise your manuscript so that it conforms to our Article format instructions at http://www.nature.com/natecolevol/info/final-submission. Refer also to any guidelines provided in this letter.

* Include a revised version of any required reporting checklist. It will be available to referees (and, potentially, statisticians) to aid in their evaluation if the manuscript goes back for peer review. A revised checklist is essential for re-review of the paper.

Please use the link below to submit your revised manuscript and related files:

[REDACTED]

Note: This URL links to your confidential home page and associated information about manuscripts you may have submitted, or that you are reviewing for us. If you wish to forward this email to co-authors, please delete the link to your homepage.

We hope to receive your revised manuscript within four to eight weeks. If you cannot send it within this time, please let us know. We will be happy to consider your revision so long as nothing similar has been accepted for publication at Nature Ecology & Evolution or published elsewhere.

Nature Ecology & Evolution is committed to improving transparency in authorship. As part of our efforts in this direction, we are now requesting that all authors identified as ‘corresponding author’ on published papers create and link their Open Researcher and Contributor Identifier (ORCID) with their account on the Manuscript Tracking System (MTS), prior to acceptance. ORCID helps the scientific community achieve unambiguous attribution of all scholarly contributions. You can create and link your ORCID from the home page of the MTS by clicking on ‘Modify my Springer Nature account’. For more information please visit please visit www.springernature.com/orcid.

Please do not hesitate to contact me if you have any questions or would like to discuss these revisions further.

We look forward to seeing the revised manuscript and thank you for the opportunity to review your work.

[REDACTED]

Reviewer expertise:

as before

Reviewers' comments:

Reviewer #1 (Remarks to the Author):

This is Simon again. The authors have done an exceedingly thorough job in responding to most my last review. The math in the appendix all appears to be correct, and anything I do not mention here I feel they did an excellent job on. However, I don't think that the authors have addressed my main concern (what I called concern #3), and without it, I don't think this work should be published in Nature E&E (like, I might be willing to accept it in Oikos or a theory journal).

Let me try to be more clear this time. My understanding of this paper is that the authors are: (a) giving a somewhat new hypothesis for tree species coexistence (i.e. intraspecific clustering); and (b) claiming that CTFS plots give empirical evidence supporting this hypothesis. I believe that the theory is sound, and that intraspecific clustering could promote coexistence. What I am skeptical of is that intraspecific clustering is actually occurring (because if it is, it would mean that if a species became rare, that there would be sections of the forest that would become more empty). In other words, I think that the authors have done (a), but not (b). I think that the modeling work that they showed in their response is correct, but addresses point (a) (i.e. whether the mechanism could work) rather than (b) (i.e. whether there is evidence for the main underlying assumption of the model). I think if they can do (b), then this deserves to be published. But I don't think that a paper belongs in Nature E&E if it proposes a partially new hypothesis that seems farfetched, and does not give empirical evidence to support the main underlying assumption.

Last time I said:
"Their basic model relies on the assumption that as a species becomes rarer, they encounter conspecifics less, and still encounter roughly the same number of heterospecifics. This mechanism does not work if the number of heterospecifics increases (unless Janzen-Connell effects are important)."
I see that I misspoke here. The number of heterospecifics can increase, but it must be less than the amount that conspecific density is reduced. To be more mathematically precise, I agree that the growth rate will be
tilde{lambda} = mean(lambda) + cov(lambda_x, nu_x).
For this to act as a coexistence mechanism, that covariance must be more positive (or less negative) when a species' density declines. This will happen if, when a species becomes rare, areas of high conspecific crowding (high nu_x) had fewer total competitors (i.e. lower conspecific+heterospecific density, resulting in a low lambda_x). This could happen if seeds were dispersed to different locations, but I would like to see some empirical evidence that this is occurring (I am very skeptical that it is).

I spoke correctly when I said I wanted to see a graph of abundance vs. "frequency of conspecifics". I agree with Eq R5b of their response, the population level growth rate is mean(lambda) + cov(lambda_x, nu_x).
The term nu_x should be (density of sp. j at site x)/(mean density of sp. j). However, I felt that (# of conspecific neighbors)/(total # of neighbors) is a better statistic to show, as it is less prone to artifacts (see below).

What I wanted was for the authors to show me was that individuals of a species experienced the most competition if they were where that species is most clumped. For simplicity, let me write C=(# conspecific neighbors) and H=(# heterospecific neighbors). In the first response, the authors showed that cov(C, C+H) was positive; this is in the right spirit, but it produces a statistical artifact (i.e. since there is a C on both sides, it will almost always be positive). If they did cov(C/[total C], C+H), this would give the same artifact. If the authors instead used cov(C+H, C/(C+H)), it would remove that artifact; it will show that competition is highest is places where that species is really dominant. I would like to see that that covariance is generally positive, or at least more positive for high-abundance species than low-abundance species. I feel I have been clear about wanting to see cov(C+H, C/(C+H)) in my last two reviews.

I requested using cov(C+H, C/(C+H)) because I believe it is the best test that the authors can do with their data. A really rigorous test would involve actually manipulating the forest, and showing that when a species becomes rarer, it becomes more clustered in low-competition areas; I know that this would be infeasible. It might be possible to do something with time-series data (though, again, I think that is an unreasonable bar for this paper). What I want to see before I will give this paper an "accept" is some evidence to suggest that if a species become rare, their high-density areas will be left somewhat empty, rather than just being filled by an equal number of heterospecifics. And, I would like to see something that demonstrates it empirically, rather than in a model (as they provided in their response). If the authors do not feel that cov(C+H, C/(C+H)) is a good statistic, they can try to propose a better one, but I think that cov(C+H, C/(C+H)) is the best that they can get without dynamic data.

Reviewer #3 (Remarks to the Author):

The manuscript was greatly improved through revision. However, I still have a few comments.

L105-109: Please add a definition of s_f.
L107: Does this mean n_{kfh} = Sigma_{i≠f}n_{kfi}? If so, please add the explanation.
L121-123: Are there experimental studies that check the positive correlation between competition intensity and phylogenetic relatedness? What is the mechanism underlying the correlation? Bacteria in roots, nutrient requirement, shapes of trees (competition for light), or disease (apparent competition)?
L156: What are the two variables of the normalized K-function? R and h?
L178: (Fig. 2a, b) (ii) → (Fig. 2a, b), (ii)
L204: Please add a definition of N_f^*.
L208, Eq. 8: By comparing the denominator and numerator of Eq. 8, I was wondering whether K_f can be larger than J^*. If K_f < J^*, does this indicate the Biodiversity-Ecosystem Function (BEF) relationship?
L259: Stabilizing mechanism of Chesson's coexistence theory (i.e., negative frequency-dependence in

community dynamics)?
L279: dispersal^{40} or → dispersal^{40}, or
L338: What about the spatial and temporal relative nonlinearity and storage effect?
L428: Is it possible for both of them to be positive so that N_f^* in Eq. 8 is positive?
L452: (t)>0. → (t))>0.
L594-595, 607-608: References 9 and 15 are the same. Please remove ref. 15.
L633-635: Something is wrong after "individual" in ref. 25 (twice).
Fig. 1d-f, 4a: Black solid lines in Fig. 1d-f indicate mean values whereas a black solid line in Fig. 4a represents a median value. Is it better to use a dashed black line in Fig. 4a?
Fig. 2b: When the value of k_{fh} is smaller than 1, it means segregation (L156-157). So it might be a little bit confusing to label the Y-axis "segregation". Readers may think that larger values mean segregation (as like "aggregation" of Fig. 2a).

*******************END*******************

Author Rebuttal, third revision:

Reviewers' comments:

Reviewer #1 (Remarks to the Author):

This is Simon again. The authors have done an exceedingly thorough job in responding to most my last review. The math in the appendix all appears to be correct, and anything I do not mention here I feel they did an excellent job on.

Response: Thank you! We highly appreciate your effort to improve our work.

However, I don't think that the authors have addressed my main concern (what I called concern #3), and without it, I don't think this work should be published in Nature E&E (like, I might be willing to accept it in Oikos or a theory journal). Let me try to be more clear this time. My understanding of this paper is that the authors are: (a) giving a somewhat new hypothesis for tree species coexistence (i.e. intraspecific clustering); and (b) claiming that CTFS plots give empirical evidence supporting this hypothesis. I believe that the theory is sound, and that intraspecific clustering could promote coexistence. What I am skeptical of is that intraspecific clustering is actually occurring (because if it is, it would mean that if a species became rare, that there would be sections of the forest that would become more empty). In other words, I think that the authors have done (a), but not (b). I think that the modeling work that they showed in their

response is correct, but addresses point (a) (i.e. whether the mechanism could work) rather than (b) (i.e. whether there is evidence for the main underlying assumption of the model). I think if they can do (b), then this deserves to be published. But I don't think that a paper belongs in Nature E&E if it proposes a partially new hypothesis that seems farfetched, and does not give empirical evidence to support the main underlying assumption.

> **Response:** Yes, we made an effort to answer (a), but it was not clear to us that you wanted an application to the ForestGEO data (i.e., b) since the discussions of concern 3 centred around the theoretical case where \beta_jj=\beta_jk for all species j and k.
>
> However, we agree that it makes sense to check if the spatial structures found at the nine ForestGEO plots are compatible with our theoretical mechanism. We therefore conducted the analysis to show (b) with the data of the ForestGEO plots. To assess the strength of the effect if the mechanism is operating, we conducted the analysis also with the data of our two simulations (stable and unstable). Results are shown in the new Extended Data Figure 8.

Last time I said:

"Their basic model relies on the assumption that as a species becomes rarer, they encounter conspecifics less, and still encounter roughly the same number of heterospecifics. This mechanism does not work if the number of heterospecifics increases (unless Janzen-Connell effects are important)." I see that I misspoke here. The number of heterospecifics can increase, but it must be less than the amount that conspecific density is reduced. To be more mathematically precise, I agree that the growth rate will be tilde{lambda} = mean(lambda) + cov(lambda_x, nu_x). For this to act as a coexistence mechanism, that covariance must be more positive (or less negative) when a species' density declines. This will happen if, when a species becomes rare, areas of high conspecific crowding (high nu_x) had fewer total competitors (i.e. lower conspecific+heterospecific density, resulting in a low lambda_x). This could happen if seeds were dispersed to different locations, but I would like to see some empirical evidence that this is occurring (I am very skeptical that it is).

> **Response:** Ok, we now provide empirical evidence that, when a species becomes rare, areas of higher conspecific crowding tend to have fewer total competitors. See our response below for details.

I spoke correctly when I said I wanted to see a graph of abundance vs. "frequency of conspecifics". I agree with Eq R5b of their response, the population level growth rate is

mean(lambda) + cov(lambda_x, nu_x). The term nu_x should be (density of sp. j at site x)/(mean density of sp. j). However, I felt that (# of conspecific neighbors)/(total # of neighbors) is a better statistic to show, as it is less prone to artifacts (see below).

What I wanted was for the authors to show me was that individuals of a species experienced the most competition if they were where that species is most clumped. For simplicity, let me write C=(# conspecific neighbors) and H=(# heterospecific neighbors). In the first response, the authors showed that cov(C, C+H) was positive; this is in the right spirit, but it produces a statistical artifact (i.e. since there is a C on both sides, it will almost always be positive). If they did cov(C/[total C], C+H), this would give the same artifact. If the authors instead used cov(C+H, C/(C+H)), it would remove that artifact; it will show that competition is highest is places where that species is really dominant. I would like to see that that covariance is generally positive, or at least more positive for high-abundance species than low-abundance species. I feel I have been clear about wanting to see cov(C+H, C/(C+H)) in my last two reviews.

> **Response:** We estimated for each focal species *f* of the ForestGEO data, as well as for the stable and the unstable model simulation
>
> - the covariance between the number of conspecific neighbours (i.e., n_{kff}) and the total number of neighbours (i.e., $n_{kff} + n_{kfh}$) (Extended Data Figure 8, left column), and
> - the covariance between the fraction of conspecific neighbours (i.e., $n_{kff}/(n_{kff} + n_{kfh})$) and the total number of neighbours (i.e., $n_{kff} + n_{kfh}$) (Extended Data Figure 8, middle column),
>
> and plotted them over the abundance of the focal species. The results of this analysis show that the covariance tends to be more positive for high-abundance species than low-abundance species (lines 296-302). The corresponding methods are described in lines 515-523.
>
> In a first step we conducted the analysis for the stable and unstable model simulations (Extended Data Figure 8a,b). As expected, the stable simulation showed that the covariance cov(C+H, C/(C+H)) is generally positive and tends to be more positive for high-abundance species than low-abundance species. As expected, the unstable simulation does not show this tendency. The analysis of the model simulations also showed that the abundance-covariance relationships are relatively noisy (even though the plots size was 200 ha) and that even a weak relationship is sufficient to stabilize the dynamics (lines 300-301).

In a second step, we conducted the analysis with the data of the ForestGEO plots. We find that cov(C, C+H) is indeed generally positive, and clearly more positive for high-abundance species than low-abundance species. The only exception is the Wabikon (WAB) plot, a temperate forest with the lowest number of species among the 9 plots. The Wabikon plot experienced localized logging during the 1900s. These disturbances, in addition to pronounced variation in elevation, have contributed to spatial heterogeneity of trees in some parts of the plot.

The summary statistic cov(C+H, C/(C+H)) shows similar trends as cov(C, C+H), but as expected, they are weaker. Even though Cov(C + H, C/(C + H)) is not positive for all species, the data for all plots (except the two temperate forests WAB and CBS with the lowest species richness) showed a positive trend in the covariance with abundance. This is actually a surprising result since a multitude of processes are likely to affect the neighbourhood densities at the ForestGEO forest plots (e.g., habitat association, disturbances, systematic trends in tree sizes and densities, etc.). Thus, most of the plots show a spatial structure in the neighbourhood density of individual trees that is not in conflict with the spatial mechanism underlying the fitness-density covariance in the stable model simulation.

I requested using cov(C+H, C/(C+H)) because I believe it is the best test that the authors can do with their data. A really rigorous test would involve actually manipulating the forest, and showing that when a species becomes rarer, it becomes more clustered in low-competition areas; I know that this would be infeasible. It might be possible to do something with time-series data (though, again, I think that is an unreasonable bar for this paper). What I want to see before I will give this paper an "accept" is some evidence to suggest that if a species become rare, their high-density areas will be left somewhat empty, rather than just being filled by an equal number of heterospecifics. And, I would like to see something that demonstrates it empirically, rather than in a model (as they provided in their response). If the authors do not feel that cov(C+H, C/(C+H)) is a good statistic, they can try to propose a better one, but I think that cov(C+H, C/(C+H)) is the best that they can get without dynamic data.

Response: We now present the results of both test statistics, cov(C, C+H) and cov(C+H, C/(C+H)) in Extended Data Figure 8.

We also show the empirical results for an additional test statistic (the mean number of neighbours). All three test statistics show similar results and provide evidence for our hypothesis.

Reviewer #3 (Remarks to the Author):

The manuscript was greatly improved through revision. However, I still have a few comments.

Response: Thank you for your detailed reading and helpful comments!

L105-109: Please add a definition of s_f.

Response: Done (line 105).

L107: Does this mean n_{kfh} = Sigma_{i≠f}n_{kfi}? If so, please add the explanation.

Response: Yes, we added the explanation $n_{kfh} = \sum_{i \neq f} n_{kfi}$ (line 108).

L121-123: Are there experimental studies that check the positive correlation between competition intensity and phylogenetic relatedness? What is the mechanism underlying the correlation? Bacteria in roots, nutrient requirement, shapes of trees (competition for light), or disease (apparent competition)?

Response: The review of Cadotte et al. (2017: Ecological Monographs 87: 535–551) lists experimental studies that check for a positive correlation between competition intensity and phylogenetic relatedness, and investigate more generally the question if there is a positive relationship between phylogenetic distance and ecological differences. This review is now cited, it is reference 27.

Fortunel et al. (2016) formulated the hypothesis behind the mechanism underlying the correlation between competition intensity and phylogenetic relatedness: if functional traits are phylogenetically conserved, close relatives are predicted to compete more strongly or to share more pests than distant relatives. This is mentioned in the methods (lines 370-372).

L156: What are the two variables of the normalized K-function? R and h?

Response: We added explanations of the subscript “h” and of the neighbourhood distance *R* (line 157)

L178: (Fig. 2a, b) (ii) → (Fig. 2a, b), (ii)

Response: Coma added.

L204: Please add a definition of N_f^*.

Response: Done (line 205).

L208, Eq. 8: By comparing the denominator and numerator of Eq. 8, I was wondering whether K_f can be larger than J^*. If K_f < J^*, does this indicate the Biodiversity-Ecosystem Function (BEF) relationship?

Response: This is a good point. Cases with positive abundance N_f^* and $K_f > J^*$ are only possible if both, the denominator and numerator of equation 8 are negative. However, this case does not fulfil the invasion criterion (eq. 18). This is now mentioned directly after equation 8 (lines 210-211). Only the BCI forest showed cases where $K_f > J^*$, and in this cases the species had relatively low abundance and showed regularity (i.e., $k_{ff} < 1$) together with $\alpha_{fh}/\alpha_{ff} > 1$ and $\mu_f > 1$ (see Fig. 4c).

If denominator and numerator are both positive we have always $K_f < J^*$. This can be seen by rewriting equation (8) to

$$K_f / J^* = (N_f^*/J^*)(1 - \alpha_{fh}/\alpha_{ff}) + \alpha_{fh}/\alpha_{ff} \quad \text{(R1)}$$

Thus, in general we expect $K_f < J^*$, which indicates that a multispecies forest would host more individuals than a monoculture. Yes, in a way this is a Biodiversity-Ecosystem Function (BEF) relationship. This is now mentioned in the methods after equation (13) (lines 431-433).

L259: Stabilizing mechanism of Chesson's coexistence theory (i.e., negative frequency-dependence in community dynamics)?

Response: The stabilizing mechanism in this case is a positive fitness-density covariance. This is now mentioned with reference to the corresponding methods (lines 262-263).

L279: dispersal^{40} or → dispersal^{40}, or

Response: Corrected.

L338: What about the spatial and temporal relative nonlinearity and storage effect?

Response: Thank you for noticing this omission, we added these mechanisms (lines 349-350).

L428: Is it possible for both of them to be positive so that N_f^* in Eq. 8 is positive?

Response: You mean both are larger than 1? Yes, but in this case the invasion criterion shows that the equilibrium will be unstable. This is now mentioned directly after equation 8 (lines 210-211).

This issue is related to your question to L208, Eq. 8. The condition $\alpha_{fh}/\alpha_{ff} > 1$ causes $K_f > J^*$.

L452: (t)>0. → (t))>0.

Response: Corrected.

L594-595, 607-608: References 9 and 15 are the same. Please remove ref. 15.

Response: Corrected.

L633-635: Something is wrong after "individual" in ref. 25 (twice).

Response: Corrected.

Fig. 1d-f, 4a: Black solid lines in Fig. 1d-f indicate mean values whereas a black solid line in Fig. 4a represents a median value. Is it better to use a dashed black line in Fig. 4a?

Response: Yes, we use now a dashed line.

Fig. 2b: When the value of k_{fh} is smaller than 1, it means segregation (L156-157). So it might be a little bit confusing to label the Y-axis "segregation". Readers may think that larger values mean segregation (as like "aggregation" of Fig. 2a).

Response: This is true. We changed the labels of the Y-axis and added a note to the figure legend to make this clear.

Decision Letter, third version

9th February 2021

Dear Dr. Wiegand,

Thank you for submitting your revised manuscript "Consequences of spatial patterns for coexistence in species rich plant communities" (NATECOLEVOL-20039642D). It has now been seen again by the original reviewers and their comments are below. The reviewers find that the paper has improved in revision, and therefore we'll be happy in principle to publish it in Nature Ecology & Evolution, pending minor revisions to satisfy the reviewers' final requests and to comply with our editorial and formatting guidelines. As recommended by the reviewer, please tone down some of the language in the results section, and list the significance for points in the extended data. The reviewer also feels that use of cov(C, C+H) as a method will lead to false positives and should be dropped. If we feel that these points have been adequately addressed in revision, we will not send the paper for further review.

We are now performing detailed checks on your paper and will send you a checklist detailing our editorial and formatting requirements in about a week. Please do not upload the final materials and make any revisions until you receive this additional information from us.

Thank you again for your interest in Nature Ecology & Evolution. Please do not hesitate to contact me if you have any questions.

[REDACTED]

Reviewer #1 (Remarks to the Author):

I was really impressed by the middle column of extended data Fig. 8. The upward curving graph is exactly the empirical evidence that you would need to show if the mechanism they are proposing is working. My skepticism of earlier drafts is that I didn't think they would find it (and thus, I thought

that they were proposing a mechanism that is theoretically sound, but not based in reality). I think (see below) that they have shown it.

My one concern here is that they don't list significance for the lines in extended data Fig. 8. I'm usually not a stickler for this, but a few of them (FS, BCI, and XSBN) seem like the upward curve may be caused by a single datapoint on the high-abundance end. That said, if at least 3 of their plots find a significant positive relationship between density (or ln{density}) and the covariance (which I think they should), then I think this should be published.

I do think they should consider toning down their results slightly, since not all plots show this pattern. They use words like "ubiquitous" at the beginning, and "key role" at the end, and all of their figures seem to suggest that this pattern is strongly true for all species. That said, it could also come about from things like dispersal limitation (which, as they show, doesn't promote coexistence). Thus, I think they can only really argue that this mechanism works in 7 of the 9 plots (or fewer if their covariance results are not significant). I do think it is worth mentioning that it didn't always work (so you can't distinguish between stabilizing distribution and neutral dispersal limitation). I think it is worth asking if there is anything special about those 2 plots where this doesn't happen (like, what you said about the two plots in the response was really interesting). That said, how much they tone down their results will depend on how many forests show a significant result. If 3 of 9 are significant, it suggests that this is an uncommon but nonetheless real mechanism; if it is 7 of 9, then it quite widespread.

I hope that this is the last time I need to say this (since I've said it in my last two reviews), but using cov(C, C+H) is an incorrect method that will lead to false positives. It should not be used alongside cov(C/(C+H), C+H), because one is a good method and the other is a wrong method. Please remove graphs of cov(C, C+H) from the paper, and any mention of it. I'm not going to budge on this, so unless the editor wants to overrule me, please take this out.

And, as proof that this creates an artifact, I tried simulating this myself. I assumed that there were 20 locations, and each location had a random number of individuals of each species in it (based on a pull from a Poisson distribution). This is a null case, as it assumes that any differences in density are purely due to chance, rather than because of preferences in dispersal. When you calculate cov(C, C+H), you find that there is a really clearly positive line between the covariance and density (a false positive). When you calculate cov(C/(C+H),C+H), the positive line goes away. I have attached the figures and my Python code used to generate this. I don't know why they didn't find this in their own model, and I suspect it is because the species had densities that were unrealistically similar.

(I don't have a problem with the mean number of neighbors, I'm just not sure if it says anything)

natureresearch

Our ref: NATECOLEVOL-20039642D

16th February 2021

Dear Dr. Wiegand,

Thank you for your patience as we've prepared the guidelines for final submission of your Nature Ecology & Evolution manuscript, "Consequences of spatial patterns for coexistence in species rich plant communities" (NATECOLEVOL-20039642D). Please carefully follow the step-by-step instructions provided in the personalised checklist attached, to ensure that your revised manuscript can be swiftly handed over to our production team.

Please get in contact with us immediately if you anticipate it taking more than two weeks to submit these revised files.

When you upload your final materials, please include a point-by-point response to any remaining reviewer comments.

If you have not done so already, please alert us to any related manuscripts from your group that are under consideration or in press at other journals, or are being written up for submission to other journals (see: https://www.nature.com/nature-research/editorial-policies/plagiarism#policy-on-duplicate-publication for details).

In recognition of the time and expertise our reviewers provide to Nature Ecology & Evolution's editorial process, we would like to formally acknowledge their contribution to the external peer review of your manuscript entitled "Consequences of spatial patterns for coexistence in species rich plant communities". For those reviewers who give their assent, we will be publishing their names alongside the published article.

Nature Ecology & Evolution offers a Transparent Peer Review option for new original research manuscripts submitted after December 1st, 2019. As part of this initiative, we encourage our authors to support increased transparency into the peer review process by agreeing to have the reviewer comments, author rebuttal letters, and editorial decision letters published as a Supplementary item. When you submit your final files please clearly state in your cover letter whether or not you would like to participate in this initiative. Please note that failure to state your preference will result in delays in accepting your manuscript for publication.

Cover suggestions

As you prepare your final files we encourage you to consider whether you have any images or illustrations that may be appropriate for use on the cover of Nature Ecology & Evolution.

Covers should be both aesthetically appealing and scientifically relevant, and should be supplied at the best quality available. Due to the prominence of these images, we do not generally select images featuring faces, children, text, graphs, schematic drawings, or collages on our covers.

We accept TIFF, JPEG, PNG or PSD file formats (a layered PSD file would be ideal), and the image should be at least 300ppi resolution (preferably 600-1200 ppi), in CMYK colour mode.

If your image is selected, we may also use it on the journal website as a banner image, and may need to make artistic alterations to fit our journal style.

Please submit your suggestions, clearly labeled, along with your final files. We'll be in touch if more information is needed.

Nature Ecology & Evolution has now transitioned to a unified Rights Collection system which will allow our Author Services team to quickly and easily collect the rights and permissions required to publish your work. Approximately 10 days after your paper is formally accepted, you will receive an email in providing you with a link to complete the grant of rights. If your paper is eligible for Open Access, our Author Services team will also be in touch regarding any additional information that may be required to arrange payment for your article.

Please note that you will not receive your proofs until the publishing agreement has been received through our system.

For information regarding our different publishing models please see our Transformative Journals page. If you have any questions about costs, Open Access requirements, or our legal forms, please contact ASJournals@springernature.com.

Please use the following link for uploading these materials:
https://mts-natecolevol.nature.com/cgi-bin/main.plex?el=A3Cn6Bfh3A6wTc5J2A9ftdYZXrzoCWj3f8Z3TwrjdewZ

If you have any further questions, please feel free to contact me.

[REDACTED]

Reviewer #1:
Remarks to the Author:
I was really impressed by the middle column of extended data Fig. 8. The upward curving graph is exactly the empirical evidence that you would need to show if the mechanism they are proposing is working. My skepticism of earlier drafts is that I didn't think they would find it (and thus, I thought that they were proposing a mechanism that is theoretically sound, but not based in reality). I think (see below) that they have shown it.

My one concern here is that they don't list significance for the lines in extended data Fig. 8. I'm usually not a stickler for this, but a few of them (FS, BCI, and XSBN) seem like the upward curve may be caused by a single datapoint on the high-abundance end. That said, if at least 3 of their plots find a significant positive relationship between density (or ln{density}) and the covariance (which I think

they should), then I think this should be published.

I do think they should consider toning down their results slightly, since not all plots show this pattern. They use words like "ubiquitous" at the beginning, and "key role" at the end, and all of their figures seem to suggest that this pattern is strongly true for all species. That said, it could also come about from things like dispersal limitation (which, as they show, doesn't promote coexistence). Thus, I think they can only really argue that this mechanism works in 7 of the 9 plots (or fewer if their covariance results are not significant). I do think it is worth mentioning that it didn't always work (so you can't distinguish between stabilizing distribution and neutral dispersal limitation). I think it is worth asking if there is anything special about those 2 plots where this doesn't happen (like, what you said about the two plots in the response was really interesting). That said, how much they tone down their results will depend on how many forests show a significant result. If 3 of 9 are significant, it suggests that this is an uncommon but nonetheless real mechanism; if it is 7 of 9, then it quite widespread.

I hope that this is the last time I need to say this (since I've said it in my last two reviews), but using cov(C, C+H) is an incorrect method that will lead to false positives. It should not be used alongside cov(C/(C+H), C+H), because one is a good method and the other is a wrong method. Please remove graphs of cov(C, C+H) from the paper, and any mention of it. I'm not going to budge on this, so unless the editor wants to overrule me, please take this out.

And, as proof that this creates an artifact, I tried simulating this myself. I assumed that there were 20 locations, and each location had a random number of individuals of each species in it (based on a pull from a Poisson distribution). This is a null case, as it assumes that any differences in density are purely due to chance, rather than because of preferences in dispersal. When you calculate cov(C, C+H), you find that there is a really clearly positive line between the covariance and density (a false positive). When you calculate cov(C/(C+H),C+H), the positive line goes away. I have attached the figures and my Python code used to generate this. I don't know why they didn't find this in their own model, and I suspect it is because the species had densities that were unrealistically similar.

(I don't have a problem with the mean number of neighbors, I'm just not sure if it says anything)

natureresearch

nature portfolio

Author Checklist

**Manuscript:**	NATECOLEVOL-20039642D
**Author:**	Thorsten Wiegand
**Article type:**	Article

Title:

Consequences of spatial patterns for coexistence in species rich plant communities

Abstract:

Ecology cannot yet fully explain why so many tree species coexist in natural communities such as tropical forests. A major difficulty is to link individual-level processes to community dynamics. We propose a combination of spatial data of trees, spatial statistics, and dynamical theory to reveal the relationship between spatial patterns and population-level interaction coefficients and their consequences for multispecies dynamics and coexistence. Here we show that the emerging population-level interaction coefficients have for a broad range of circumstances a simpler structure than their individual-level counterparts, which allows for an analytical treatment of equilibrium and stability conditions. Mechanisms such as animal seed dispersal that result in clustering of recruits, but spatially independent of their parents, leads to a rare-species advantage and coexistence of otherwise neutral competitors. Linking spatial statistics with theories of community dynamics offers new avenues for explaining species coexistence and calls for rethinking community ecology through a spatial lens.

Your paper will be accompanied by the editor's summary and subject terms below. Please let us know if there are any inaccuracies.

Editor's Summary:

Tree spatial data, spatial statistics, and dynamical theory reveal the relationship between spatial patterns and population-level interaction coefficients and their consequences for multispecies dynamics and coexistence

Additional Abstract Requests:

☐ The abstract — which should be roughly 150 words long and contain no references — should serve both as a general introduction to the topic and a non-technical summary of your main results and their implications. It should contain a brief account of the background and rationale of the work, followed by a statement of the main conclusions introduced by the phrase 'Here we show' or some equivalent phrase. Because we hope that researchers in a wide range of disciplines will be interested in your work, the abstract should be as accessible as possible, explaining essential but specialized terms concisely. We encourage you to show your abstract to colleagues outside of your direct field of expertise to uncover any problematic concepts.

☐ Please ensure that all key species/genes/locations are mentioned in the abstract

Author information

Please review your complete author list to verify that it is complete and accurate. We ask that you consult with your coauthors to ensure that all names, affiliations, and titles are represented correctly. Note that if any authors are added or removed after this point then all authors will be requested to provide approval documentation that could potentially delay the production of your paper.

The following changes must be made to the author information:

☐ Please supply an email address for each corresponding author.

Article structure

We can accommodate up to 8 display items (Figures or Tables) in the main article and up to 10 Extended Data figures, which will be integrated into the full-text HTML version of your paper and will be appended to the online PDF. Each Figure, Table and Extended Data figure must fit easily within an A4 page (260 x 179 mm). Please ensure that the number and size of your Figures, Tables and Extended Data figures fulfil these requirements to avoid any delay in the acceptance of your Article.

To comply with our format and optimise the presentation of data in your Article, we suggest the following changes to the number of Figures, Tables and Extended Data:

Word limit

- [ ] No changes required

Please ensure your main manuscript file includes the following sections:

- [ ] Title
- [ ] Author list
- [ ] Affiliations
- [ ] Abstract
- [ ] Main text
- [ ] References
- [ ] Acknowledgements
- [ ] Author Contributions Statement
- [ ] Competing Interests Statement
- [ ] Figure Legends (for main text figures)
- [ ] Tables
- [ ] Methods (including Data Availability, Code Availability and Statistics subsections where relevant)

Main Text

The following changes must be made to the main text:

- [ ] Equations and symbols that will be set apart from the text must be in an editable format. Do not use embedded images for equations or symbols.
- [ ] Please remove Extended Data captions from main manuscript file

Figures, Tables & Legends

Please see the guidelines linked below for detailed instructions about how your figures should be prepared. Following these instructions will reduce the chances of delays should we need to request replacement artwork from you at a later stage.

https://www.nature.com/documents/NRJs-guide-to-preparing-final-artwork.pdf

Additional requests:

- [ ] All figures, tables and extended data figures must be cited in the main text, and numbered in the order in which they appear.
- [ ] All colour scales (for any figure) must be defined in either the figure or its associated legend.

Data & Code

Data Availability

Nature journals strongly support public availability of data and code. Please deposit the data and code used in your paper into a public data repository. If data can only be shared on request, please explain why in your Data Availability Statement, and also in the correspondence with your editor.

Please note that for some data types, deposition in a public repository is mandatory. Any restrictions on sharing of these data types must be clearly indicated in the statement and discussed with the editor. More information on our data deposition policies and available repositories can be found here:

https://www.nature.com/nature-research/editorial-policies/reporting-standards#availability-of-data

Data Availability Statements

All published manuscripts reporting original research in Nature Research journals must include a data availability statement, as a separate section before the References and under the heading "Data Availability".

The data availability statement must make the conditions of access to the "minimum dataset" that are necessary to interpret, verify and extend the research in the article, transparent to readers.

This minimum dataset may be provided through deposition in public community/discipline-specific repositories, custom proprietary repositories or general repositories like Figshare, Zenodo and Dryad. Providing large datasets in supplementary information is strongly discouraged and the preferred approach is to make data available in repositories. Scientific Data, a Nature Research journal, maintains a list of approved and recommended data repositories to support researchers seeking suitable repositories for their data (https://www.nature.com/sdata/policies/repositories).

The Data Availability Statement should also reference any source data published alongside the paper.

If DOIs are provided, we also strongly encourage including these in the Reference list (authors, title, publisher (repository name), identifier, year).

Reporting Summaries

☐ An updated life sciences reporting summary must be completed and uploaded as a supplementary information file with the revised manuscript. All points on the reporting summary must be addressed; if needed, please revise your manuscript in response to these points. This checklist is published alongside your manuscript online. Please note that this form is a dynamic "smart pdf" and must therefore be downloaded and completed in Adobe Reader, instead of opening it in a web browser. https://www.nature.com/documents/nr-reporting-summary.pdf

Additional requests related to Data and Code:

☐ If possible, please store your code on a stable external repository rather than hosting it in the paper SI since we can't ensure it will be downloaded in an executable format. Please include the code repository URL in the Code Availability Statement

☐ Please could you explain in your data availability statement the restrictions associated with ForestGEO datasets (i.e. that you don't own them, which is why access must be requested)

End Matter

☐ Please supply an "Author Contributions" section after the "Acknowledgements" section that refers to all authors. For more information on the Author Contributions statement, please refer to our authorship policy(https://www.nature.com/nature-research/editorial-policies/authorship), and to the following Nature Editorial: https://www.nature.com/articles/4581078a.

Competing Interests

Nature Research defines Competing Interest (CI) as financial and non-financial interests (including but not limited to funding, employment, stocks, shares, patents, personal or professional relationships with individuals or institutions, and unpaid membership advocacy) that could be perceived to directly undermine the objectivity, integrity, and value of a publication, or could be seen as having an influence on the judgments and actions of authors with regard to objective data presentation, analysis, and interpretation.

Please thoroughly review our policy on Competing Interests and include a detailed statement both in your final manuscript file and in our manuscript tracking system. Please ensure the statements are identical in both. Be specific about how each point stated relates to the research and list applicable author initials, and/or patent numbers.

If there are no competing interests, a negative statement must be included.

Confirmation of Funding Support

Please confirm that all relevant funding awarded to each author is described in the Acknowledgements section. List each grant number, followed by the initials of the author who received it.

Copyright and Third Party Rights

It is your responsibility to obtain the right to use any items (figures, tables, images, videos or text boxes) that are reproduced (or adapted) from material for which you do not hold copyright and to give proper attribution to the creators of that work. This includes work that has previously been published elsewhere.

If you do not hold the copyright for any item (in whole or part), included in your paper, you must complete and return a Third Party Rights Table:

http://www.nature.com/documents/thirdpartyrights-origres.doc

For more information on what constitutes ownership by a third party, please contact our Editorial Assistant at ecoevo@nature.com

Preparing your manuscript files

Unless otherwise stated please limit individual file sizes to approximately 30MB. We strongly encourage the use of repositories for large datasets or source data due to size considerations.

- ☐ To ensure maximum visibility for your work, we may tweet about your paper following publication. If you would like us to include the Twitter handles of the first author(s), corresponding author(s), lab or institution in this tweet, please provide them in your cover letter. We would also welcome your suggestions for hashtags to use when tweeting about the work.
- ☐ Please provide Extended Data figures as individual files in .tiff, .jpg or .eps format, and provide legends for each in the Inventory of Supporting Information. Please also ensure that each Extended Data figure is cited wihtin the main manuscript text.
- ☐ Please supply the main Supplementary Information file as a single PDF file.

Forms to complete

Editorial Policy Checklist

Please update and upload a final version of the Editorial Policy Checklist with your revised manuscript files. A blank Editorial Policy Checklist can be found via the link below. Note that this form is a dynamic 'smart pdf' and must be downloaded and completed in Adobe Reader.

Please update your current checklist or download from: https://www.nature.com/documents/nr-editorial-policy-checklist.zip

Inventory of Supporting Information

The inventory must be completed with details of all Supplementary Information, Extended Data and Source Data files.

Please download from: www.nature.com/documents/Inventory_of_Supporting_Information_2021.docx

You will need to upload:

- ☐ A point-by-point response to any remaining issues raised by the reviewers (if relevant)
- ☐ Editorial Policy Checklist
- ☐ Completed Third Party Rights Table (if relevant)
- ☐ Main Article File in Microsoft Word or LaTeX format
- ☐ Separate Figure files
- ☐ Separate Extended Data files (if relevant)

- ☐ Inventory of Supporting Information
- ☐ A Supplementary Information file as a separate, single PDF (if relevant)
- ☐ Supplementary Table, Video, Audio, Data, Software files (if relevant)
- ☐ Reporting Summary (if relevant)

natureresearch

Author Rebuttal, second revision:

9th February 2021

Dear Dr. Wiegand,

Thank you for submitting your revised manuscript "Consequences of spatial patterns for coexistence in species rich plant communities" (NATECOLEVOL-20039642D). It has now been seen again by the original reviewers and their comments are below. The reviewers find that the paper has improved in revision, and therefore we'll be happy in principle to publish it in Nature Ecology & Evolution, pending minor revisions to satisfy the reviewers' final requests and to comply with our editorial and formatting guidelines.

> **Reply:** We are happy that you'll be happy in principle to publish our manuscript in Nature Ecology & Evolution. It was quite some work to respond during the various revisions to the reviewer comments, but this greatly improved the manuscript. We therefore want to thank the reviewers again for their valuable inputs!

As recommended by the reviewer, please tone down some of the language in the results section, and list the significance for points in the extended data. The reviewer also feels that use of cov(C, C+H) as a method will lead to false positives and should be dropped. If we feel that these points have been adequately addressed in revision, we will not send the paper for further review.

> **Reply:** Following the recommendations, we toned down our language in the results section, we dropped the results of cov(C, C+H) in Extended Data Figure 8, and we added p-values to express the significance of the trends shown the figure.

Reviewer #1 (Remarks to the Author):

I was really impressed by the middle column of extended data Fig. 8. The upward curving graph is exactly the empirical evidence that you would need to show if the mechanism they are proposing is working. My skepticism of earlier drafts is that I didn't think they would find it (and thus, I thought that they were proposing a mechanism that is theoretically sound, but not based in reality). I think (see below) that they have shown it.

> **Reply:** We are happy to hear that! Note that we now can even explain with our theory why the 3 temperate forest plots do not show the spatial patterns required for our mechanism.

My one concern here is that they don't list significance for the lines in extended data Fig. 8. I'm usually not a stickler for this, but a few of them (FS, BCI, and XSBN) seem like the upward curve may be caused by a single datapoint on the high-abundance end. That said, if at least 3 of their plots find a significant positive relationship between density (or ln{density}) and the covariance (which I think they should), then I think this should be published.

> **Reply:** This is a good idea, thank you for proposing this! We calculated now a p-value for the null hypothesis that the slope of the relationship is not positive and included the p-value directly into the figure (lines 297-304). More details of the regression and raw data are presented in the Supplementary Data Table.
>
> The slope is significantly positive for 4 plots (the 3 sub-tropical plots FS, GTS, and DHS), and for the Baihua plot. The relationship for XSBN is just not significant ($p = 0.057$), and BCI shows a non-significant tendency ($p = 0.17$). The 3 temperate plots that do not show the positive tendency in the covariance all show larger clustering of species with lower abundances than the other plots (i.e., the slope of the power law < -0.5 but > -0.5 for the other plots; see Extended Data Figure 8), which nicely explains why they do not show the positive tendency (lines 301-304).

I do think they should consider toning down their results slightly, since not all plots show this pattern. They use words like "ubiquitous" at the beginning, and "key role" at the end, and all of their figures seem to suggest that this pattern is strongly true for all species. That said, it could also come about from things like dispersal limitation (which, as they show, doesn't promote coexistence).

> **Reply:** Ok. We checked these statements and similar ones and toned them down or where more specific (line 305). We removed the "ubiquitous" (line 64) since the spatial patterns required for the positive fitness-density covariance are frequent but not ubiquitous. We also removed the term "key role" and replaced it by "important role" (line 315).

Thus, I think they can only really argue that this mechanism works in 7 of the 9 plots (or fewer if their covariance results are not significant). I do think it is worth mentioning that it didn't always work (so you can't distinguish between stabilizing distribution and neutral dispersal limitation). I think it is worth asking if there is anything special about those 2 plots where this doesn't happen (like, what you said about the two plots in the response was really interesting). That said, how much they tone down their results will depend on how many forests show a significant result. If

3 of 9 are significant, it suggests that this is an uncommon but nonetheless real mechanism; if it is 7 of 9, then it quite widespread.

Reply: Yes, this is true. The mechanism will not always work, and our theory also predicts that it will not work if the relationship between clustering and aggregation is a strong negative power law (i.e., much higher clustering for less abundant species).

Indeed, the unstable simulation and the 3 temperate plots (i.e., WAB, CBS and SCBI) where our mechanism did clearly not work show strong negative power laws with exponents < -0.5, but all other plots show a weak or no power-law relationship. To illustrate this we added the clustering-abundance relationships to extended data figure 8.

We revised the paragraph (lines 297-304) accordingly, now mentioning also the reason for the failure of the 3 temperate plots to show this pattern.

I hope that this is the last time I need to say this (since I've said it in my last two reviews), but using cov(C, C+H) is an incorrect method that will lead to false positives. It should not be used alongside cov(C/(C+H), C+H), because one is a good method and the other is a wrong method. Please remove graphs of cov(C, C+H) from the paper, and any mention of it. I'm not going to budge on this, so unless the editor wants to overrule me, please take this out. And, as proof that this creates an artifact, I tried simulating this myself. I assumed that there were 20 locations, and each location had a random number of individuals of each species in it (based on a pull from a Poisson distribution). This is a null case, as it assumes that any differences in density are purely due to chance, rather than because of preferences in dispersal. When you calculate cov(C, C+H), you find that there is a really clearly positive line between the covariance and density (a false positive). When you calculate cov(C/(C+H),C+H), the positive line goes away. I have attached the figures and my Python code used to generate this. I don't know why they didn't find this in their own model, and I suspect it is because the species had densities that were unrealistically similar.

Reply: Ok, we removed the column with the cov(C, C+H) analysis.

(I don't have a problem with the mean number of neighbors, I'm just not sure if it says anything)

Reply: To simplify we removed also this column, you are right, it does not really add information.

natureresearch

Final Decision Letter:

1st March 2021

Dear Dr Wiegand,

We are pleased to inform you that your Article entitled "Consequences of spatial patterns for coexistence in species rich plant communities", has now been accepted for publication in Nature Ecology & Evolution.

Before your manuscript is typeset, we will edit the text to ensure it is intelligible to our wide readership and conforms to house style. We look particularly carefully at the titles of all papers to ensure that they are relatively brief and understandable.

The subeditor may send you the edited text for your approval. Once your manuscript is typeset you will receive a link to your electronic proof via email, with a request to make any corrections within 48 hours. If you have queries at any point during the production process then please contact the production team at rjsproduction@springernature.com. Once your paper has been scheduled for online publication, the Nature press office will be in touch to confirm the details.

Acceptance of your manuscript is conditional on all authors' agreement with our publication policies (see www.nature.com/authors/policies/index.html). In particular your manuscript must not be published elsewhere and there must be no announcement of the work to any media outlet until the publication date (the day on which it is uploaded onto our web site).

Nature Ecology & Evolution is a Transformative journal and offers an immediate open access option through payment of an article-processing charge (APC) for papers submitted after 1 January, 2021 . In the event that authors choose to publish under the subscription model, Nature Research allows authors to self-archive the accepted manuscript (the version post-peer review, but prior to copy-editing and typesetting) on their own personal website and/or in an institutional or funder repository where it can be made publicly accessible 6 months after first publication, in accordance with our self-archiving policy. Please review our self-archving policy for more information.

Several funders require deposition the accepted manuscript (AM) to PubMed Central or Europe PubMed Central. To enable compliance with these requirements, Nature Research therefore offers a free manuscript deposition service for original research papers supported by a number of PMC/EPMC participating funders. If you do not choose to publish immediate open access, we can deposit the accepted manuscript in PMC/Europe PMC on your behalf, if you authorise us to do so.

In approximately 10 business days you will receive an email with a link to choose the appropriate publishing options for your paper and our Author Services team will be in touch regarding any additional information that may be required.

You will not receive your proofs until the publishing agreement has been received through our system.

If you have any questions about our publishing options, costs, Open Access requirements, or our legal forms, please contact ASJournals@springernature.com

An online order form for reprints of your paper is available at https://www.nature.com/reprints/author-reprints.html. All co-authors, authors' institutions and authors' funding agencies can order reprints using the form appropriate to their geographical region.

We welcome the submission of potential cover material (including a short caption of around 40 words) related to your manuscript; suggestions should be sent to Nature Ecology & Evolution as electronic files (the image should be 300 dpi at 210 x 297 mm in either TIFF or JPEG format). Please note that such pictures should be selected more for their aesthetic appeal than for their scientific content, and that colour images work better than black and white or grayscale images. Please do not try to design a cover with the Nature Ecology & Evolution logo etc., and please do not submit composites of images related to your work. I am sure you will understand that we cannot make any promise as to whether any of your suggestions might be selected for the cover of the journal.

You can now use a single sign-on for all your accounts, view the status of all your manuscript submissions and reviews, access usage statistics for your published articles and download a record of your refereeing activity for the Nature journals.

To assist our authors in disseminating their research to the broader community, our SharedIt initiative provides you with a unique shareable link that will allow anyone (with or without a subscription) to read the published article. Recipients of the link with a subscription will also be able to download and print the PDF.

You can generate the link yourself when you receive your article DOI by entering it here: <a href="http://authors.springernature.com/share">http://authors.springernature.com/share<a>.

Yours sincerely,

[REDACTED]

P.S. Click on the following link if you would like to recommend Nature Ecology & Evolution to your librarian http://www.nature.com/subscriptions/recommend.html#forms

** Visit the Springer Nature Editorial and Publishing website at www.springernature.com/editorial-and-publishing-jobs for more information about our career opportunities. If you have any questions please click here.**
